# RNA Polyadenylation Sites on the Genomes of Microorganisms, Animals, and Plants

**DOI:** 10.1371/journal.pone.0079511

**Published:** 2013-11-18

**Authors:** Xiu-Qing Li, Donglei Du

**Affiliations:** 1 Molecular Genetics Laboratory, Potato Research Centre, Agriculture and Agri-Food Canada, Fredericton, New Brunswick, Canada; 2 Quantitative Methods Research Group, Faculty of Business Administration, University of New Brunswick, Fredericton, New Brunswick, Canada; Nazarbayev University, Kazakhstan

## Abstract

Pre–messenger RNA (mRNA) 3′-end cleavage and subsequent polyadenylation strongly regulate gene expression. In comparison with the upstream or downstream motifs, relatively little is known about the feature differences of polyadenylation [poly(A)] sites among major kingdoms. We suspect that the precise poly(A) sites are very selective, and we therefore mapped mRNA poly(A) sites on complete and nearly complete genomes using mRNA sequences available in the National Center for Biotechnology Information (NCBI) Nucleotide database. In this paper, we describe the mRNA nucleotide [i.e., the poly(A) tail attachment position] that is directly in attachment with the poly(A) tail and the pre-mRNA nucleotide [i.e., the poly(A) tail starting position] that corresponds to the first adenosine of the poly(A) tail in the 29 most-mapped species (2 fungi, 2 protists, 18 animals, and 7 plants). The most representative pre-mRNA dinucleotides covering these two positions were UA, CA, and GA in 17, 10, and 2 of the species, respectively. The pre-mRNA nucleotide at the poly(A) tail starting position was typically an adenosine [i.e., A-type poly(A) sites], sometimes a uridine, and occasionally a cytidine or guanosine. The order was U>C>G at the attachment position but A>>U>C≥G at the starting position. However, in comparison with the mRNA nucleotide composition (base composition), the poly(A) tail attachment position selected C over U in plants and both C and G over U in animals, in both A-type and non-A-type poly(A) sites. Animals, dicot plants, and monocot plants had clear differences in C/G ratios at the poly(A) tail attachment position of the non-A-type poly(A) sites. This study of poly(A) site evolution indicated that the two positions within poly(A) sites had distinct nucleotide compositions and were different among kingdoms.

## Introduction

One of the central mechanisms in gene regulation is messenger RNA (mRNA) polyadenylation, that is, polyadenylation [poly(A)] tailing at the 3′ end [1]–[3], which strongly affects mRNA export, stability, and functionality and is critical for the development of living organisms [4]–[6]. An essential step in the maturation of all mRNAs, 3′ processing is a tightly coupled two-step reaction: endonucleolytic cleavage at the poly(A) site (i.e., the cleavage site), followed by direct addition of a poly(A) tail [7]–[9]. There are only a few exceptions: nontemplated addition of nucleotides to the 3′ end in some *Arabidopsis* mRNAs [10] and human mRNAs [11], including some ribosomal RNAs (rRNAs) [12]; and lack of polyadenylation after cleavage in histone mRNAs in some metazoan species [7], [8], [13]. The RNA polymerase II complex is involved with pre-mRNA processing, and the nascent RNA most often remains associated with the chromosomal locus being transcribed until processing is complete [14]. Cleavage factor is also a key regulator of 3′-untranslated region (3′UTR) length [15]. The cleavage sites occur at a UA or CA dinucleotide in the mRNA of seven yeast alcohol dehydrogenase genes [16] and favourably at CA or UA in expressed sequence tags (ESTs) of *Vitis vinifera* [17]. When a simian virus 40 (SV40) viral nucleotide fragment carrying the AAUAAA polyadenylation signal motif was processed in vitro in human cell extract, CA at the cleavage site was enriched [18], suggesting that a CA dinucleotide at the poly(A) site is preferred for human mRNA cleavage. However, mutational analysis of the poly(A) site of SV40 found no evidence for the involvement of a CA dinucleotide motif in cleavage site selection in HeLa spinner cells [19]. Nevertheless, the phenomenon of CA dinucleotide enrichment at the cleavage site is supported by pooled poly(A) site data from five mammals [20]. Considerable differences in base composition were observed between poly(A) sites and a few bases away from the sites in human mRNAs [21]. Polyadenylation sites tend to be less sensitive to deoxyribonuclease I, according to bioinformatic analysis of human DNA functional elements [22]. However, the differences in nucleotide frequency at poly(A) sites among subkingdoms such as non-mammal animals, dicot plants, and monocot plants are still unclear. Furthermore, little information is available about whether these poly(A) site base differences among subkingdoms are simple reflections of the mRNA base composition differences among subkingdoms or are indeed a positive or negative selection.

Research has greatly enriched our knowledge on polyadenylation signals upstream or downstream of the poly(A) site. The cleavage and polyadenylation specificity factor and the cleavage stimulation factor likely interact with the upstream AAUAAA hexamer [often considered the poly(A) signal] and downstream U/GU-rich element in the poly(A) site region [23], [24]. Many human and mouse mRNAs that have AAUAAA or a variant motif harbour multiple cleavage sites, and therefore the cleavage process of polyadenylation is considered to be largely imprecise [25]. Some of the latest software packages for poly(A) site prediction are based mainly on the upstream motif AAUAAA or similar motifs, with assistance from various less-conserved downstream motifs [24], [26], [27]. The machine-learning approach can improve poly(A) motif prediction [28]. Yeast RNAs containing regulatory elements, likely noncoding RNAs regulating gene expression, were found to also be polyadenylated [21]. In *Trichomonas vaginalis*, a parasitic protozoan, the UAAA tetranucleotide has a role equivalent to that of the metazoan consensus AAUAAA in the mRNA polyadenylation signal [29]. Even though many mRNAs have alternative polyadenylation cleavage sites as a mechanism in gene expression regulation [20], [25], [30]–[32], approximately 78% of mRNAs use canonical A[A/U]UAAA polyadenylation signals in purified mouse embryonic skin stem cells and their daughter lineages [30]. In an analysis of polyadenylation signal motifs in six eukaryotic species, the use and conservation of the canonical AAUAAA element varied widely and were especially weak in plants and yeast, a finding that leads to the hypothesis that overall polyadenylation efficiency is a function of all elements and that no single element is universally required for processing [33]. This rich knowledge on mRNA poly(A) signal motifs has stimulated the need for further research to determine whether the poly(A) sites themselves play any important role in the determination of poly(A) sites and whether the sites are simply arranged by the polyadenylation signal motifs. Large-scale comparative data analysis of poly(A) sites among different groups of mammal mRNAs (rich in AAUAAA) and plant mRNAs (poor in AAUAAA) may provide a clue as to whether poly(A) sites are determined mainly by AAUAAA and similar motifs.

Sets of ESTs are used to study poly(A) site motifs by EST clustering [17], [34]–[38]. Although very useful for studying poly(A) sites, the EST approach is not designed for comparisons among species and kingdoms. The reason is that most EST libraries are tissue-specific or growth condition–specific and therefore contain an over-representation of the set of genes expressed in that tissue or treatment condition. Furthermore, EST sequences are generated from a single sequencing run without verification, and EST sequence quality is not comparable to the quality of the transcript sequences in the National Center for Biotechnology Information (NCBI) mRNA database. Libraries of ESTs can have contamination from internal priming and polyadenylated rRNAs, because human rRNA can sometimes be polyadenylated [12] and because not all the EST sets submitted to NCBI have had the rRNA ESTs pre-eliminated. In contrast, the mRNA sequences in the NCBI Nucleotide database (www.ncbi.nlm.nih.gov) have usually been verified by repeated sequencing from both the 5′ and 3′ ends of complementary DNA (cDNA) clones, and therefore artificial poly(A) sites resulting from internal priming can be largely eliminated.

We hypothesized that the precise location of a poly(A) site is not determined purely or randomly by the upstream or downstream motifs; the right nucleotide features at poly(A) sites are also needed during the determination or fine-tuning of the site locations. These poly(A) site features must also vary during evolution; in other words, they likely have general patterns that differ among large kingdoms such as plants and animals. Characterization of nucleotide composition selection and the precise poly(A) sites in many species across kingdoms should provide very valuable knowledge with respect to understanding the process and mechanisms of mRNA polyadenylation, regulating gene expression, studying gene termination, and improving the accuracy of poly(A) site prediction. We also hypothesized that certain selections of poly(A) sites are predominant in certain species or kingdoms, because they are evolutionarily related. One of the best approaches for verifying our hypotheses is to map polyadenylated mRNA sequences to their corresponding genomes in many species across kingdoms. This approach makes it possible to examine the evolutionary differences among species and to study both the nucleotide attachment position and the poly(A) tail starting position at the cleavage site.

The objective of this study was to compare the nucleotide compositions of poly(A) cleavage sites across species and main kingdoms. We screened most mRNA in the NCBI Nucleotide database, identified the poly(A) tailed mRNA, eliminated all duplicated sequences [according to the 100–base region upstream of the poly(A) site], and mapped these unique sequences to their corresponding species genomes (**Table S1** for chromosome and genome ID list). Since we applied zero tolerance to mismatch during mapping, we eliminated the transcripts that had non-templated synthesis of non-adenosine nucleotides prior to polyadenylation.

To facilitate the description of the poly(A) site, we call the mRNA nucleotide that is directly in attachment with the poly(A) tail “**the poly(A) tail attachment position of the poly(A) site**” and call the pre-mRNA nucleotide that corresponds to the first adenosine of the poly(A) tail “**the poly(A) tail starting position of the poly(A) site**”. We also compared the two groups of poly(A) sites: **A-type poly(A) sites**, which have a pre-mRNA adenosine at the poly(A) tail starting position, and **non-A-type poly(A) sites**, which do not have an adenosine at the pre-mRNA poly(A) tail starting position. For the A-type poly(A) site, the poly(A) tail attachment position and the starting position correspond likely to the 5′ nucleotide and the 3′ nucleotide covering the potential cleavage site (bond), respectively. For the non-A-type poly(A) site, the poly(A) tail attachment position and the starting position correspond exactly to the 5′ nucleotide and the 3′ nucleotide covering the cleavage site (bond), respectively. We present the nucleotide composition features of all these positions or groups of poly(A) sites in the eukaryote kingdoms.

## Results

### Analyzed Sequences and Mapped Poly(A) Sites

In total, 2 fungi, 2 protozoan protists, 18 animal species, and 7 plant species were chosen for detailed analysis because their genomes are either complete or nearly complete and because they have relatively more poly(A) sites mapped to their genomes than do other species in the same kingdoms (**Table 1**). In total, 1,615,332 mRNA sequences of these 29 species from the NCBI mRNA database were analyzed (**Table 1**). These sequences were searched against poly(A) mRNA criteria, including having 12 A’s continuously at the 3′ end and having no N’s in the 100 bases upstream of and the 100 bases downstream of the poly(A) tail starting position [i.e., no N’s in the 201–nucleotide genomic segment per poly(A) site]. In total, 304,087 mRNA sequences met the criteria for poly(A) tailed mRNA. We eliminated the duplicated mRNA according to the 100 bases upstream of the pre-mRNA nucleotide replaced by the poly(A) tail, and we obtained 210,474 unique sequences. This mRNA region represents mainly the 3′UTR. In order to avoid any ambiguity in counting the nucleotide types at the poly(A) site, we set the mRNA–genome alignment/mapping to zero tolerance for mismatches. Some poly(A) tailed mRNAs could not be mapped, because they may have been different alleles from the ones on the reference genome even though they may or may not have been from the same individual, or they may have been from different genotypes of the species. After they had been aligned against their corresponding genomes, 97,285 unique mRNA sequences [for the 100 bases upstream of the poly(A) site] were mapped unambiguously (**Table 1**).

**Table 1:** Species analyzed, polyadenylation [poly(A)] messenger (mRNA) identified, and poly(A) sites mapped.

Species	Common nameor description	mRNA screened (n)^a^	Unique GI^b^ poly(A)mRNA (n)	Unique sequences ofpoly(A) mRNA (n)^c^	Unique sites mapped (n)^d^	Total sites mapped (n)	Sites/uniquesequences (n)^e^
**Fungi**							
*Neurospora crassa*	Fungus	10,016	25	25	19	38	2.0
*Schizosaccharomyces pombe*	Fungus	5,518	56	54	26	26	1.0
**Protozoa (human parasites)**							
*Plasmodium falciparum*	Malaria parasite	9,306	65	63	42	42	1.0
*Trypanosoma cruzi*	Trypanosomiasis parasite	20,194	136	131	52	1,523	29.3
**Non-mammalian animals**							
*Apis mellifera*	Honey bee	129,998	1,333	894	187	187	1.0
*Caenorhabditis elegans*	Nematode	26,279	550	490	389	392	1.0
*Danio rerio*	Zebrafish	54,094	26,689	16,112	7,246	10,662	1.5
*Drosophila melanogaster*	Fruit fly	26,626	1,555	1,341	954	966	1.0
*Gallus gallus*	Chicken	27,311	4,795	2,610	788	803	1.0
*Taeniopygia guttata*	Zebra finch	19,108	4,287	3,026	808	836	1.0
**Mammals–non-primates**							
*Bos taurus*	Cattle	39,822	18,250	9,992	2,679	2,719	1.0
*Canis lupus familiaris*	Dog	35,672	613	344	108	125	1.2
*Equus caballus*	Horse	20,302	304	178	97	101	1.0
*Mus musculus*	Mouse	118,303	36,900	25,645	8,709	12,474	1.4
*Oryctolagus cuniculus*	Rabbit	20,924	482	309	224	316	1.4
*Rattus norvegicus*	Rat	77,124	40,798	24,857	14,139	34,791	2.5
*Sus scrofa*	Pig	47,730	21,222	16,933	8,114	12,634	1.6
**Mammals–Primates**							
*Callithrix jacchus*	New World monkey	24,437	140	106	79	118	1.5
*Homo sapiens*	Human	210,773	67,909	44,519	30,499	39,591	1.3
*Macaca mulatta*	Rhesus monkey	34,034	581	464	380	1,152	3.0
*Pan troglodytes*	Chimpanzee	58,231	842	488	436	849	1.9
*Pongo abelii*	Orangutan	33,876	6,714	3,827	1,965	2,036	1.0
**Plants**							
*Arabidopsis thaliana*	Arabidopsis	88,337	9,368	8,987	4,431	4,505	1.0
*Medicago truncatula*	A diploid alfalfa	49,799	1,409	1,404	536	833	1.6
*Oryza sativa (japonica)*	Rice	26,177	1,004	975	693	715	1.0
*Populus trichocarpa*	Poplar	44,262	3,813	3,741	1,371	1,393	1.0
*Solanum tuberosum*	Potato	5,056	2,915	2,463	139	139	1.0
*Sorghum bicolor*	Sorghum	187,622	3,246	2,788	1,685	1,719	1.0
*Zea mays*	Maize	164,401	48,086	37,708	10,490	21,265	2.0
Total		1,615,332	304,087	210,474	97,285	152,950	

a Downloaded from the NCBI Nucleotide database (http://www.ncbi.nlm.nih.gov/) with searching keywords “species name [organism] and mRNA [title]”. The downloaded sequences were mainly verified mRNA sequences, but some expressed sequence tags (ESTs) were also included if they had been submitted to GenBank under mRNA rather than ESTs. For *S. bicolor*, however, in order to have a sufficient number of monocot plant species analyzed, the mRNA database transcripts were supplemented with EST transcripts to ensure a large number of poly(A) sites mapped in the species. Further research is needed to test whether this supplement altered the nucleotide type frequencies of mapped poly(A) sites in *S. bicolor*.

b GI: NCBI sequence identification number.

c Must have met three criteria: 1) the mRNA sequence upstream of the poly(A) tail must have at least 100 bases; 2) the mRNA has a poly(A) tail at the 3′ end; and 3) the pure poly(A) tail must have at least 12 A’s.

d The mRNA–genome mapping was set to zero tolerance for mismatches.

e No information was available on which site is more functional than another if a unique mRNA sequence is mapped to more than one location on the genome. The species average for the number of sites per unique mRNA in the higher eukaryote group (animals and plants) was 1.36 if all the species were included, and was 1.26 when rhesus monkey and chimpanzee were excluded.

Most of the sequences were mapped to single-copy genes, and some of the sequences were mapped to more than one location on the genome. The unique mRNA sequences were therefore mapped to 152,950 sites in total (**Table 1**). We counted these sites indiscriminately because there is no information about which site is functionally more important than any other and because the genomes we used were complete or nearly complete. The trypanosomiasis parasite (*Trypanosoma cruzi*) and rhesus monkey (*Macaca mulatta*) were exceptional: each *T. cruzi* mRNA sequence mapped on average to 29 locations, and each rhesus monkey mRNA sequence mapped to three locations (**Table 1**). It is unclear whether these multiple locations were due to the quality of the assembled genome (in that it was highly enriched with certain repetitive genes) or to the mRNA sets used, but it is known that the rhesus monkey and chimpanzee (*Pan troglodytes*) mRNA databases contained mainly entries computed using EST sequences. In rhesus monkey, the most-repeated genes were zinc finger protein 91–like protein and the olfactory receptor 1F12–like proteins. In the mapped chimpanzee genomic locations, the most-repeated gene was a gene encoding a mitochondrial acyl-CoA dehydrogenase (mRNA NM_001110816.1). The mapped genome locations in rhesus monkey were also rich in multiple adenosines immediately after poly(A) sites. Chimpanzee had this issue to a certain degree as well. Although further research is required to find out whether this particular richness in multiple A’s at poly(A) sites in these two species is due to their biology or due to EST-based computation, the mRNA datasets for these species likely had more internal priming and more ESTs than did the other species. Therefore, we excluded these two species from the calculations of the comparison among animals and plants. When all the animal and plant species were counted, the average number of mapped sites for each mRNA was 1.36. When rhesus monkey and chimpanzee were excluded, the average number of sites for each animal or plant mRNA that was mapped became 1.26.

### Dinucleotide Covering the Pre-mRNA Cleavage Site

The most representative dinucleotide that covers both the poly(A) tail attachment position and the tail starting position of the cleavage site is UA (or TA for DNA) in 15 species, CA in 10 species, and interestingly, GA in two species (*T. cruzi* and zebrafish [*Danio rerio*]) (**Table 2**). On average, the most representative dinucleotide at the poly(A) site was UA in plants (38%), UA in non-mammal animals (36%), and CA in mammals (37%, or 34% if *M. mulatta* and *P. troglodytes* were excluded) (**Table 2**). The extremely high frequency of CA (79%) at the poly(A) site in *M. mulatta* was due to multiple-copy genes. When all the mapped gene copies by the same unique mRNA [representing a cluster in which all mRNAs have the same 100 bases upstream of the poly(A) tail starting position] were counted as 1, the CA frequency at poly(A) sites became much smaller (45%), but CA was still the most frequent in *M. mulatta*. The high CA frequency at poly(A) sites in that species was due in part to the contribution of the high-copy-number genes (the zinc finger protein 91–like protein and the olfactory receptor 1F12–like proteins). The high UA frequency at poly(A) sites in chimpanzee was due in part to a highly repeated acyl-CoA dehydrogenase. In *T. cruzi*, 90% of the mRNA poly(A) sites used GA. In maize (*Zea mays*), UA was used in only 26% of the sites, even though it was the most representative dinucleotide (**Table 2**). The CC and CU dinucleotides were each at 10% in maize, although they were very low in other species (overall means of 1% and 2%, respectively) (data not shown). In the diploid alfalfa species *Medicago truncatula*, the UA dinucleotide alone accounted for 60%, which was much higher than the sum of all other dinucleotide types (**Table 2**). In rabbit (*Oryctolagus cuniculus*), UA, CA, and GA were used at quite similar frequencies (31%, 25%, and 30%, respectively) in the poly(A) sites, with GA as the second most frequently used (**Table 2**). Within the 25 animal and plant species, five animals (*Bos taurus*, *Equus caballus*, *D. rerio*, *Homo sapiens*, and *Mus musculus*) and three plants (*Sorghum bicolor*, *Arabidopsis thaliana*, and *Z. mays*) showed differences of only 0% to 5% between UA and CA dinucleotide frequencies at the poly(A) sites (**Table 2**). This large-scale analysis provided an overview of species-level and kingdom-level selections on mRNA poly(A) site types. Clearly, each species or species group had its own selection on the dinucleotide at the poly(A) sites, and the UA or CA dinucleotide was not always the most abundant.

**Table 2:** UA, CA, and GA dinucleotides at the polyadenylation [poly(A)] tail sites^a^.

Species	UA (%)	CA (%)	GA (%)	Sum (UA+CA+GA) (%)
**Fungi and protozoa**				
*Neurospora crassa*	5	**37**	32	74
*Plasmodium falciparum*	**55**	26	19	100
*Schizosaccharomyces pombe*	**69**	19	12	100
*Trypanosoma cruzi*	6	4	**90** b	99
**Non-mammalian animals**				
*Apis mellifera*	**53**	16	22	91
*Caenorhabditis elegans*	**42**	19	17	78
*Danio rerio*	29	25	**35**	89
*Drosophila melanogaster*	**42**	32	23	97
*Gallus gallus*	25	**34**	24	82
*Taeniopygia guttata*	23	**30**	26	79
Non-mammal average	**36**	26	25	86
**Mammals**				
*Bos taurus*	28	**30**	25	83
*Callithrix jacchus*	**44**	31	18	93
*Canis lupus familiaris*	**41**	30	15	86
*Equus caballus*	**38**	36	16	89
*Homo sapiens*	**35**	30	20	84
*Macaca mulatta*	11	**79** c	7	98
*Mus musculus*	**33**	28	25	87
*Oryctolagus cuniculus*	**31**	25	30	86
*Pan troglodytes*	**59**	28	10	97
*Pongo abelii*	27	**33**	23	83
*Rattus norvegicus*	23	**48**	17	88
*Sus scrofa*	22	**44**	22	89
Mammal average	33	**37**	19	89
Mammals without *M. mulatta* and *P. troglodytes*	32	34	21	87
**Plants**				
*Arabidopsis thaliana*	31	**31** d	13	76
*Medicago truncatula*	**60**	22	11	93
*Oryza sativa (japonica)*	**43**	30	14	87
*Populus trichocarpa*	27	**33**	13	73
*Solanum tuberosum*	**40**	24	22	87
*Sorghum bicolor*	**38**	33	15	86
*Zea mays*	**26**	21	12	59
Plant average	**38**	28	14	80
Overall mean	**35**	30	22	87

a Each of the upstream 100–base messenger (mRNA) sequences (or 3′-untranslated regions) directly adjacent to the poly(A) tail starting position is unique, but all the mapped genomic sites (1.36 sites on average per unique animal or plant mRNA) from a single unique mRNA were counted. The bold numbers mean the frequency of the most frequent dinucleotide at the mapped poly(A) sites.

b If all the multiple copies mapped by a unique mRNA were counted as one unique poly(A) site, the dinucleotide GA was still the most frequent (38.46%) in *T. cruzi*.

c If all the multiple copies mapped by a unique mRNA were counted as one unique poly(A) site, the dinucleotide CA was still the most frequent (45%) in *M. mulatta*.

d CA: 31.48%; UA: 31.10%.

### Pre-mRNA Nucleotide at the Poly(A) Tail Starting Position

The genomic or pre-mRNA nucleotide at the poly(A) starting position was usually an adenosine [i.e., A-type poly(A) site] in all 29 species (**Table 3**), with that nucleotide reaching approximately 87% in the overall mapped poly(A) sites (**Table 3**). The observed A-type poly(A) site percentage was significantly higher (*P*<0.000,0001) than the percentage expected for the random model in the alignment mapping in every species (**Table 3**). Clearly, poly(A) tailing selects for adenosine at the poly(A) tail starting position of the poly(A) site. The top species that had 93% or more A-type poly(A) sites included two human protozoan parasites (*Plasmodium falciparum* and *T. cruzi*), four animals (*Drosophila melanogaster*, *Callithrix jacchus*, *M. mulatta*, and *P. troglodytes*), and one plant species (*M. truncatula*) (**Table 3**). A total of three plants–maize, poplar (*Populus trichocarpa*), and *Arabidopsis*–had low adenosine frequency (74%) at the pre-mRNA poly(A) tail starting position (**Table 3**). The next most common poly(A) site was uridine, which reached only 7% on average (**Table 3**). This large-scale study quantitatively confirmed the dominance of A-type poly(A) sites for mRNA in all the examined species of the eukaryote kingdoms.

**Table 3:** Pre–messenger RNA nucleotide replaced by the polyadenylation [poly(A)] tail in different species^a^.

Species	Mapped sites (n)	Observed A%^b^	Observed U%	Observed C%	Observed G%	RNA A content(%)^c^	Random modelsite A%^d^
**Fungi and parasite protists**							
*Neurospora crassa*	38	**74** **	11	16	0	28	48
*Plasmodium falciparum*	42	**100** **	0	0	0	42	72
*Schizosaccharomyces pombe*	26	**100** **	0	0	0	32	55
*Trypanosoma cruzi*	1,523	**99** **	1	0	0	10	17
Mean	407	**93** **	3	4	0	28	48
**Non-mammalian animals**							
*Apis mellifera*	187	**91** **	5	2	2	38	65
*Caenorhabditis elegans*	392	**78** **	14	1	6	30	51
*Danio rerio*	10,662	**89** **	6	3	3	32	55
*Drosophila melanogaster*	966	**97** **	1	1	1	37	64
*Gallus gallus*	803	**82** **	10	4	4	31	53
*Taeniopygia guttata*	836	**79** **	11	4	6	30	52
Mean	2,308	**86** **	8	2	4	33	57
**Mammals**							
*Bos taurus*	2,719	**83** **	8	4	4	31	52
*Callithrix jacchus*	118	**93** **	3	1	3	30	52
*Canis lupus familiaris*	125	**86** **	7	2	6	30	51
*Equus caballus*	101	**89** **	5	2	4	30	50
*Homo sapiens*	39,591	**84** **	7	5	3	30	51
*Macaca mulatta*	1,152	**98** **	1	1	1	24	41
*Mus musculus*	12,474	**87** **	7	4	3	33	56
*Oryctolagus cuniculus*	316	**86** **	6	5	3	30	51
*Pan troglodytes*	849	**97** **	2	0	0	26	45
*Pongo abelii*	2,036	**83** **	7	5	5	31	52
*Rattus norvegicus*	34,791	**88** **	7	3	2	35	59
*Sus scrofa*	12,634	**89** **	5	4	3	30	50
Mean	8,909	**89** **	5	3	3	30	51
**Plants**							
*Arabidopsis thaliana*	4,505	**76** **	14	6	5	30	50
*Medicago truncatula*	833	**93** **	3	1	3	30	51
*Oryza sativa (japonica)*	715	**87** **	6	4	3	27	46
*Populus trichocarpa*	1,393	**73** **	17	4	6	27	45
*Solanum tuberosum*	139	**87** **	6	4	2	27	46
*Sorghum bicolor*	1,719	**86** **	8	4	3	26	44
*Zea mays*	21,265	**59** **	19	15	7	26	45
Mean	4,367	**80** **	10	5	4	27	47
**Overall mean**	**5,274**	**87** **	**7**	**4**	**3**	**30**	**51**

a Each of the upstream 100–base messenger RNA (mRNA) sequences directly adjacent to the poly(A) tail starting position is unique, but all the mapped genomic sites (1.36 sites on average per unique animal or plant mRNA) from a single unique mRNA were counted.

b The observed percentage of pre-mRNA adenosine replaced by the poly(A) tail is the genomic adenosine frequency at the site corresponding to the first adenosine of mRNA poly(A) tails. The statistical significance marked on the poly(A) site adenosine frequency was based on the chi-square test using the observed values (number of observed A and number of observed non-A) against the random model theoretical values (number of theoretical A and number of theoretical non-A).

** significance at *P*<0.01 in *N. crassa*, at *P*<0.001 in *P. falciparum* and *S. pombe*, and at *P*<0.000,000,0001 in all other species.

c The average A nucleotide content in the 100 nucleotides upstream of the poly(A) site of mRNA. The poly(A) site percentage is not correlated with the mRNA adenosine content (*r*<0.09).

d The theoretical adenosine poly(A) site frequency in the alignment from the random model. If the A nucleotide percentage in mRNA is *p*, the adenosine poly(A) site from the alignment will be *p*+*p*(1−*p*) = *p*(2−*p*), where (1−*p*) is the non-A nucleotide content (See **File S1**). The observed poly(A) site adenosine frequency is clearly not random; it is significantly higher (*P* = 0) than its random model value.

Note that the nucleotide at the mRNA poly(A) site is usually an A and occasionally a U. Poly(A) sites with C or G are rare.

**Figure 1:**
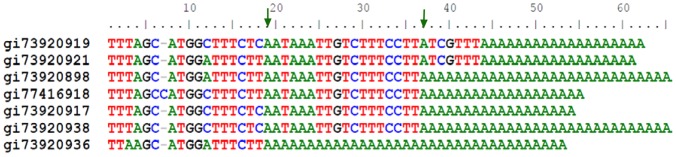
The alignment of 3′-end sequences of potato Kunitz-type protease inhibitor messenger RNAs. Note that each polyadenylation [poly(A)] tail likely starts from an adenosine (as indicated by the arrows).

The adenosine preference is illustrated in **Figure 1**, in which highly similar mRNA sequences of potato Kunitz-type protease inhibitors are aligned. Because of their similarity, these inhibitors are likely to have the same or a similar DNA template. The poly(A) site of the middle four transcripts (starting from gi:73920898) is likely an A (corresponding to position 37). Similarly, the last transcript (gi:73920936) has a poly(A) site from an A corresponding to position 19.

### Nucleotide Composition at the Poly(A) Tail Attachment Position of Non-A-Type Poly(A) Sites

We analyzed the nucleotide composition at the poly(A) tail attachment position of the group of poly(A) sites whose starting positions are not adenosines [i.e., non-A-type poly(A) sites]. For example, the poly(A) tail starting position of the poly(A) tail in human H4H mRNA (gi:15012054) is a “g” at the site GCUgAAAACC [the small “g” is cleaved off and corresponds to the first A of the poly(A) tail]. The overall frequency of the attachment nucleotide in this non-A-type poly(A) site group followed the order of U, C, and G (39.6%, 34.2%, and 26.1%, respectively) after the sum of non-A-type poly(A) sites had been normalized to 100% (**Table S2**). Of the 25 animal and plant species, 13 had higher frequency of U than of C, one had equal frequencies of U and C, and 11 had lower frequency of U than of C at the attachment positions (**Table S2**). In most animal species, C and G frequencies at the attachment positions were approximately equal (**Table S2**). At this attachment nucleotide, G is much less frequent in plants than in animals (**Table S2**).

### Comparison with mRNA Nucleotide Composition

To verify whether the nucleotide composition (base composition) at the poly(A) starting position is a simple reflection of the nucleotide composition of the mRNA region, we compared the nucleotide compositions between the poly(A) starting positions and the 100–nucleotide 3′UTR sequences. We found clear variation for the mRNA nucleotide composition among the kingdoms: on average, the adenosine content was 28% in fungi and protozoa, 33% in non-mammal animals, 30% in mammals, and 27% in plants (**Table 3**). Plants had lower adenosine content than animals did in this mRNA region. There was no significant correlation (*r* = 0.09) between the mRNA adenosine content and the adenosine percentage at the cleavage nucleotide replaced by the poly(A) tail (**Table 3**). These results demonstrate that poly(A) site selection is not a simple, random reflection of the genomic nucleotide composition.

### Internal Priming

To verify whether the observed adenosine predominance at the pre-mRNA poly(A) tail starting position is falsely inflated from internal priming, we analyzed the percentage of the mapped mRNA sequences that had poly(A) stretches in the mapped genomic/pre-mRNA poly(A) site region in each species. Many mammalian genes (11.5% on average, mainly from rhesus monkey, chimpanzee, and pig [*Sus scrofa*]) had 12 or more adenosines at the mapped candidate poly(A) sites, whereas only 0.3% of plant genes had such multiple adenosines in the same region (**Table S3**). The estimated contribution of internal priming in general was very low (**Table S3**) because of the nature of the mRNA database (resequencing verification), and the poly(A) tail was much longer than the internal multiple-A sequence. The overall average for adenosine frequencies at the poly(A) tail starting position was 86% after the false tails caused by internal priming had been taken off. In plants at least, internal priming did not contribute significantly to the adenosine frequency at the poly(A) site (**Table S3**). When the estimated internal contribution was totally eliminated, a process that included removal of all the mRNA poly(A) sites that had 12 A’s on the genome, the adenosine frequency at the poly(A) site was still 80% on average (**Table S3**), which again demonstrated the predominance of adenosine at the poly(A) sites.

### Comparative Study of C/G Ratios

**Figure 2:**
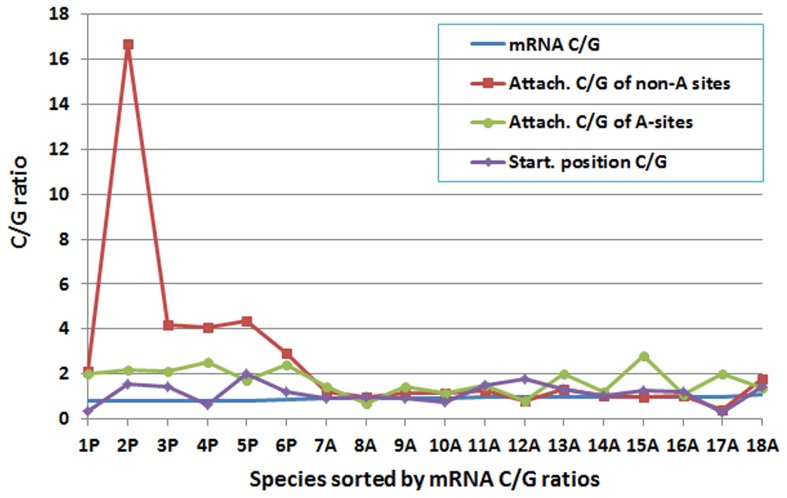
The C/G ratios (sorted from smallest (#1) to largest (#18) in messenger RNA (mRNA) sequences, the polyadenylation [poly(A)] tail attachment position of A-type poly(A) sites, the poly(A) tail attachment position of non-A-type poly(A) sites, and the poly(A) tail starting position. P: plant; A: animal. Species order from 1 to 18 is *Medicago truncatula* (1P), sorghum (2P), rice (3P), poplar (4P), maize (5P), *Arabidopsis thaliana* (6P), chicken (7A), zebrafish (8A), orangutan (9A), zebra finch (10A), human (11A), rabbit (12A), pig (13A), cattle (14A), rat (15A), mouse (16A), dog (17A), and fruit fly (18A). Note that a) plants and animals are clearly separated by the mRNA C/G ratios; b) plants strongly selected C over G at the poly(A) tail attachment position when the tail starting position was not an A; c) plants still favourably selected C over G when the tail starting position was an A; d) plants usually (in four of six species) favoured C over G to a certain degree at the poly(A) tail starting position; and e) animals did not demonstrate this preference for C over G at either the poly(A) tail attachment position or the starting position, with the exception of rat (species 15A), which showed a certain preference for C over G at the poly(A) tail attachment positions when the starting position was an A.

To carry out a comparative study of mRNA nucleotide composition and nucleotide composition at the poly(A) sites, we analyzed the mRNA nucleotide composition for the 99–nucleotide segment directly upstream from the poly(A) tail attachment position in 12 animal species and six plant species whose genomes are complete or nearly complete (**Figures 2** **and** **3**). The C/G ratios in the mRNA sequences, the poly(A) tail attachment position of A-type poly(A) sites, the poly(A) tail attachment position of non-A-type poly(A) sites, and the poly(A) tail starting position are presented in **Figure 2**. In the non-A-type poly(A) sites, the nucleotide composition at the poly(A) tail attachment position demonstrated a strong selection of C over G in plants. Plants still favourably selected C over G at the poly(A) tail attachment position when the tail starting position was an A. Of the six plant species, four favoured C over G to a certain degree at the poly(A) tail starting position as well (**Figure 2**). Animals did not demonstrate a clear preference for C over G at either the poly(A) tail attachment position or the starting position, with the exception of chimpanzee (species 7A) and rat (*Rattus norvegicus*; species 16A), which showed a certain preference for C over G at the poly(A) tail attachment positions when the starting position was an adenosine.

**Figure 3:**
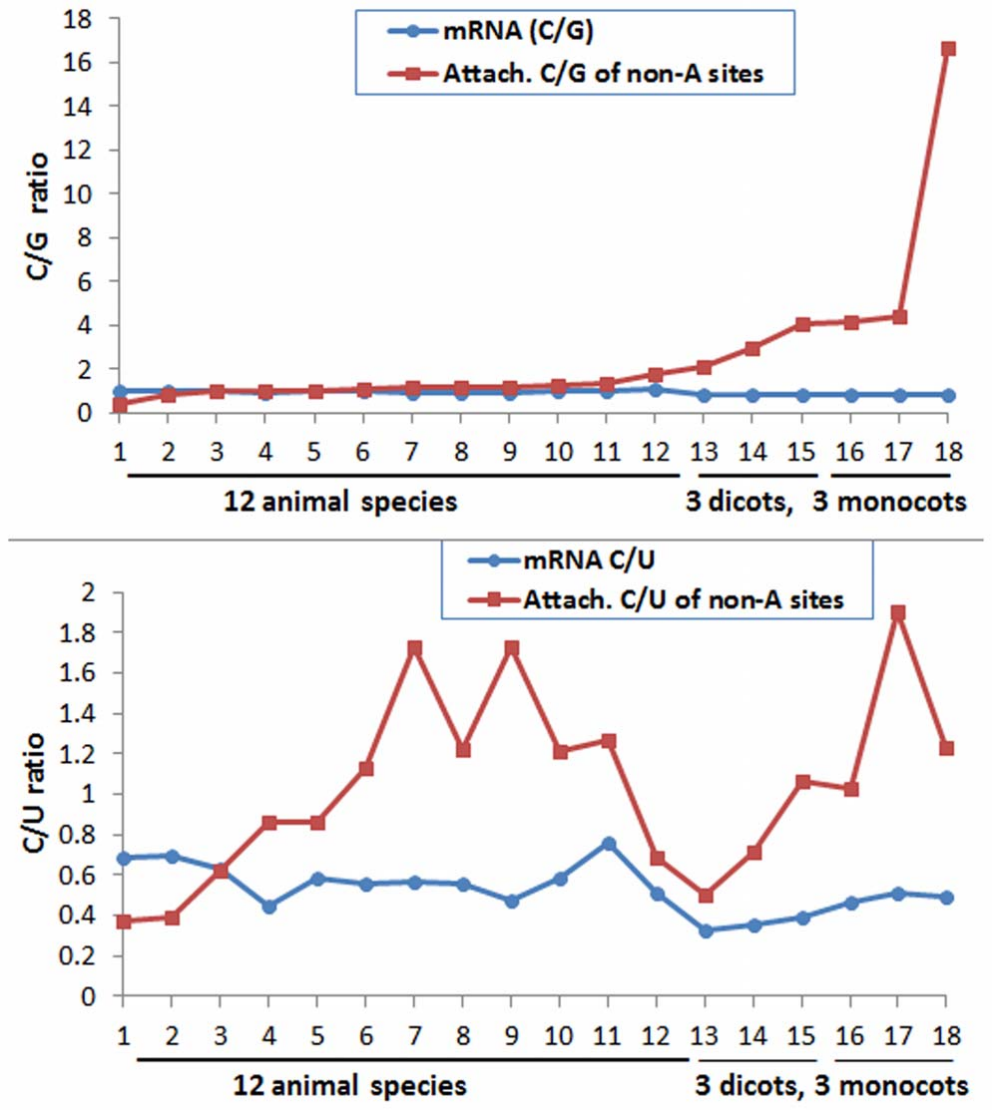
The C/G and C/U ratios at the polyadenylation [poly(A)] tail attachment position of non-A-type poly(A) transcripts. The 18 species, namely 12 animals, 3 dicot plants, and 3 monocot plants (3 cereals: rice, maize, and sorghum), were sorted from smallest (1) to largest (18) by the C/G ratios at the poly(A) tail attachment position of non-A-type poly(A) sites. The order of animal species from 1 to 12 is dog, rabbit, rat, zebrafish, mouse, cattle, zebra finch, orangutan, chicken, human, pig, and fruit fly. The three dicot plants are, in order, *Medicago truncatula*, *Arabidopsis thaliana*, and poplar. The three monocot plants are, in order, rice, maize, and sorghum. **A:** Comparison between the poly(A) tail attachment position C/G ratio and the messenger RNA (mRNA) C/G ratio. The mRNA C/G ratio is from the 99–nucleotide upstream region starting from, but not included, the poly(A) tail attachment position. There was an overall negative correlation between the poly(A) tail attachment nucleotide C/G ratio and the mRNA C/G ratio (*r* = −0.53, *P*<0.05). Note that in animals, the poly(A) tail attachment position C/G ratio (1.05 on average) on non-A-type poly(A) sites was only slightly (1.08 times) greater than the mRNA C/G ratio (0.97 on average). In plants, however, the poly(A) tail attachment nucleotide C/G ratio (5.73 on average) was about sevenfold higher than the mRNA C/G ratio (0.83 on average), suggesting that plants strongly selected C over G as the poly(A) tail attachment nucleotide. **B:** Comparison between the poly(A) tail attachment position C/U ratio of non-A-type poly(A) sites and the messenger RNA (mRNA) C/U ratio. The 18 species were sorted from smallest (#1) to largest (#18) by the C/G ratios at the poly(A) tail attachment position of non-A-type poly(A) sites, as in the top panel. Note that the C/U ratio of the poly(A) tail attachment position of non-A-type poly(A) sites was greater than the messenger RNA C/U ratio in most species and the results suggest a selection of C over U at the poly(A) tail attachment position.

Interestingly, the C/G ratio for the attachment position of the non-A-type poly(A) sites could be used to clearly separate the 18 species into three groups, as follows: animal species (the smallest C/G ratios), dicotyledonous plants (medium C/G ratios), and monocotyledonous cereal plants (the largest C/G ratios) (**Figure 3**). There was an overall negative correlation between the nucleotide C/G ratio at the poly(A) tail attachment position and the mRNA C/G ratio (*P* = −0.53). In animals, the C/G ratio at the poly(A) tail attachment position (1.05 on average) was only slightly (1.08 times) greater than the mRNA C/G ratio (0.97 on average). In plants, however, the nucleotide C/G ratio at the poly(A) tail attachment position (5.73 on average) was about sevenfold higher than the mRNA C/G ratio (0.83 on average), suggesting that plants strongly selected C over G as the poly(A) tail attachment nucleotide.

### Comparative Study of C/U Ratios

**Figure 4:**
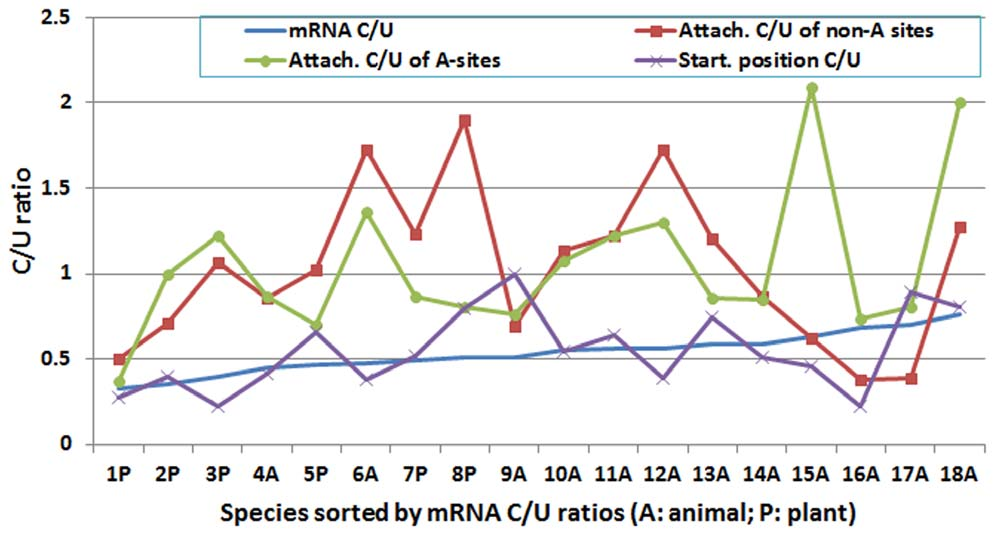
The C/U ratios in messenger RNA (mRNA) sequences, the polyadenylation [poly(A)] tail attachment position of A-type poly(A) sites, the poly(A) tail attachment position of non-A-type poly(A) sites, and the poly(A) tail starting position. The species were sorted from smallest (1) to largest (18) by their mRNA C/U ratios. The order of species from 1 to 18 is *Medicago truncatula* (1P), *Arabidopsis thaliana* (2P), poplar (3P), zebrafish (4A), rice (5P), chicken (6A), sorghum (7P), maize (8P), fruit fly (9A), cattle (10A), orangutan (11A), zebra finch (12A), human (13A), mouse (14A), rat (15A), dog (16A), rabbit (17A), and pig (18A). Note that a) there was no correlation between the C/U ratio at the poly(A) site and the mRNA C/U ratio; b) the C/U ratios at the poly(A) attachment position were usually much higher than the mRNA C/U ratios, a finding that means that C was positively selected over U to a certain degree at the poly(A) tail attachment position; and c) the poly(A) starting position did not have this preference for C over U.

There was no correlation between the C/U ratio at the poly(A) site [regardless of the poly(A) tail attachment position or the starting position] and the mRNA C/U ratio (**Figure 4**). The C/U ratios were usually higher at the poly(A) attachment positions than the mRNA C/U ratios were in plants and animals (except in dog [*Canis lupus familiaris*], rabbit, and chimpanzee), which means that C was positively selected over U to a certain degree at the poly(A) tail attachment positions in both A-type (**Figure 4**) and non-A-type poly(A) sites (**Figures 3** **and** **4**). The poly(A) starting position did not have this preference for C over U (**Figure 4**). Rat was particularly exceptional in comparison with other species in terms of the C/U ratio at the poly(A) sites. Among the 34,791 poly(A) sites mapped in rat, the C/U ratio at the poly(A) tail attachment position did not show any special preference for C over U when the poly(A) tail starting position was not an A (non-A type), but C selection was 3.3 times higher than U selection at the same attachment position in A-type poly(A) sites (**Figure 4**).

### Comparative Study of G/U Ratios

**Figure 5:**
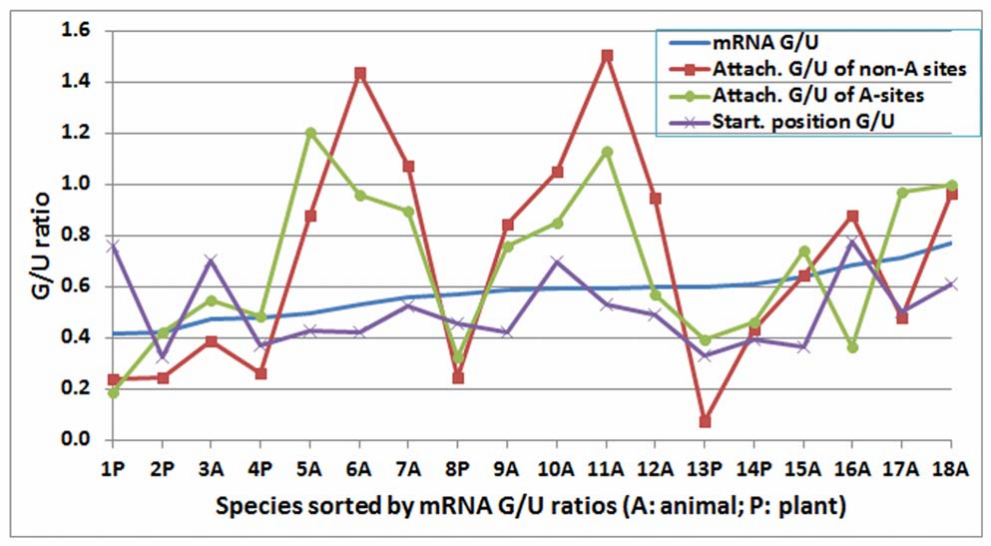
The G/U ratios in messenger RNA (mRNA) sequences, the polyadenylation [poly(A)] tail attachment position of A-type poly(A) sites, the poly(A) tail attachment position of non-A-type poly(A) sites, and the poly(A) tail starting position. The species were sorted from smallest (#1) to largest (#18) by their mRNA G/U ratios. The order of species from 1 to 18 is *Medicago truncatula* (1P), *Arabidopsis thaliana* (2P), fruit fly (3A), poplar (4P), zebrafish (5P), chicken (6A), cattle (7A), rice (8P), mouse (9A), orangutan (10A), zebra finch (11A), human (12A), sorghum (13P), maize (14P), rat (15A), dog (16A), rabbit (17A), and pig (18A). Note that the G/U ratios for the poly(A) tail starting positions were generally lower than the mRNA G/U ratios, a finding that means that G was less favoured than U at the poly(A) tail starting position. Note also that the G/U ratio at the poly(A) tail attachment position did not correlate with the mRNA G/U ratio, but eight species highly favoured G over U at the poly(A) attachment position, regardless of whether the poly(A) tail starting position was an adenosine. The correlation between the poly(A) tail attachment position of non-A-type poly(A) sites (“Attach. G/U of non-A sites”) and the poly(A) tail attachment position of A-type poly(A) sites (“Attach. G/U of A sites”) was significant (*r* = 0.74, *P*<0.05).

The G/U ratios in the poly(A) tail starting position were generally lower than the mRNA G/U ratios in 15 of 18 animal and plant species, a finding that means that at the poly(A) tail starting position, G was less favoured than U (**Figure 5**). Only *M. truncatula* and fruit fly (*D. melanogaster*) showed G/U ratios at the poly(A) tail starting position that were higher than their mRNA G/U ratios. Again, there was no correlation in terms of G/U ratios between mRNA and the poly(A) tail starting position.

The G/U ratios in the mRNA sequences, the poly(A) tail attachment position of A-type poly(A) sites, the poly(A) tail attachment position of non-A-type poly(A) sites, and the poly(A) tail starting position are presented in **Figure 5**. The G/U ratio at the poly(A) tail attachment position did not correlate with the mRNA G/U ratio, but eight species highly favoured G over U at the poly(A) attachment position, regardless of whether the poly(A) tail starting position was an adenosine. For the poly(A) sites that were not an adenosine at the poly(A) tail starting position, all the plants had a positive selection of U over G, whereas most animals favoured G over U at the poly(A) tail attachment position (**Figure 5**). The nucleotide compositions at the poly(A) tail attachment position showed a significant correlation between the A-type and non-A-type poly(A) site transcript groups (*r* = 0.74, *P*<0.05), a finding that means that there is at least one unknown factor, other than a GA or UA dinucleotide, influencing nucleotide selection at the poly(A) attachment position.

## Discussion

This study focused on mRNA polyadenylation, which is executed by the nuclear cleavage and polyadenylation machinery [39], [40]. However, it is known that rRNA and small nucleolar RNA (snoRNA) polyadenylation requires exosome-associated components [2], and adenylation usually stimulates mRNA degradation in bacteria [2], [41]. We could not conduct a similar analysis of the polyadenylation sites of these non-mRNA transcripts, because NCBI GenBank had very few polyadenylated bacterial RNA and plant/animal rRNA and snoRNA. Further research is required to verify whether these non-mRNAs also have poly(A) site selection similar to that of mRNA.

We found that the most representative dinucleotide at the poly(A) sites could be UA, CA, or GA, depending on the species. Although the most-frequent dinucleotide at the poly(A) sites was CA in mammals, as previously reported [18], [20], with all the mammal species pooled together (**Table 2**), we found that UA was actually the most frequent in approximately half of the mammal species if each species was analyzed individually (**Table 2**). The mRNA poly(A) sites in most plant species were found to clearly prefer UA (**Table 2**), but the CC and CU dinucleotides were also frequently used in maize. The GA dinucleotide was the most abundant at the poly(A) sites in the protozoan species *T. cruzi* and in zebrafish (**Table 2**). This information is novel because it is likely the first time that GA was found to be the most favourable poly(A) site in some species and that UA was found to be preferred in seven of eight plant species.

The need for large-scale analysis is also demonstrated by the gene-order study. We analyzed 747 sequenced species and 2,061 genomes/chromosomes and detected clear differences in gene direction among kingdoms [42]. There are clearly evolutionary changes in gene directional orders. All the archaeans, bacteria, and protozoa analyzed have genes characterized mainly by same-direction neighbours, with up to 391 genes in tandem in the protozoan *Leishmania infantum*; in contrast, fungi and photosynthetic protists have genes characterized mainly by opposite-direction neighbours [42]. The large-scale analysis of gene orders clearly indicated the risk involved in automatically extending the conclusions from a small set of genes to the genome or to other species or kingdoms in general without actual study. Similarly, for the mRNA poly(A) sites, even though considerable knowledge has been obtained mainly from several model species such as SV40, yeast, and human, actual analyses are still important if we want to know about poly(A) site selection in each species and kingdom. In this study, clear differences among kingdoms and subkingdoms were detected for features at mRNA poly(A) sites.

For most species in the present study, the contribution of internal priming [hybridization to internal poly(A) stretches by oligo (dT) in cDNA synthesis] to A-type poly(A) site frequencies was also likely very low, even though internal priming was one of the challenges in previous studies [38], [43]. Internal priming can account for about 12% in EST poly(A) tails [43]. In our study, internal poly(A) stretches with 12 A’s could be found in proportions ranging from approximately 0% of mRNAs in potato (*Solanum tuberosum*) to approximately 81% of mRNAs in the rhesus monkey (**Table S3**). The exact contribution of internal priming to the percentage of mapped A-type poly(A) sites is unknown, but the actual alteration of the estimated adenosine frequency at the poly(A) tail starting position should be much smaller than the percentages of these internal poly(A) stretches. This is for the following reasons: a) in many species such as plants, only 0.3% of mRNA transcripts have an internal multiple-adenosine sequence in the mapped region, whereas the A-type (i.e., adenosine) poly(A) site in the plant mRNA population was 80%; b) most transcripts with the A stretches have an adenosine at the poly(A) site, and therefore the internal priming at an internal adenosine does not change the counted adenosine percentage; c) the chance for internal priming is much smaller than the chance for priming at the true poly(A) tail, because the poly(A) tail can be longer than 250 nucleotides [44], which is many times longer than the internal adenosine stretches; and d) the mRNA sequences that we used were from the NCBI Nucleotide (not EST) database, in which most mRNA entries (despite having some ESTs) had been verified by repeated sequencing and by authors’ experimental support for the 3′ end region if they include a poly(A) tail in the submission to GenBank.

Poly(A) site selection is not random, as shown by the clear differences among species, the high similarity of site-type frequencies among relatively close species, and the general difference between animals and plants. It is known at least that different alleles of RNA processing genes that cleave different RNA regions can be maintained in plant populations under appropriate selection pressures [45]. The diversity in the nucleotide predominance at poly(A) sites in the eukaryote kingdoms might be also due to the specific selection pressures. Experimental evolution and mutation-induction approaches may be useful for the identification of genes that influence the nucleotide frequencies at poly(A) sites.

The predominance of adenosine at the poly(A) tail starting position is likely biologically important for many genes. In a T1 ribonuclease assay of SV40 mRNA in human cell extract, conversion of the A at the site to either U or C shifted the poly(A) site to the adjacent adenosine downstream [18]. Thus, the nucleotide on the 3′ end of mRNAs has an important influence on polyadenylation, and although an adenosine at the site “is not essential, cleavage might still require an adenosine near that position” [18]. The agreement between the SV40 mRNA T1 mapping results and the mRNA–genome bioinformatics mapping for the 29 species in the present study strongly suggests that the predominance of adenosine at the pre-mRNA nucleotide replaced by the poly(A) tail is biologically important for mRNA maturation. The present study demonstrated the predominance of adenosine and quantified the frequencies of different nucleotides at the pre-mRNA poly(A) tail starting position in 29 species covering all the eukaryote kingdoms.

For the non-A-type poly(A) sites, the poly(A) tail attachment nucleotide and the poly(A) tail starting position nucleotide at the poly(A) site could be precisely and accurately determined in the pre-mRNA and genome. For example, the poly(A) site nucleotide replaced by the poly(A) tail was a “g” in AUU*g*CUCAA of the *A. thaliana* histone H2B mRNA (gi:1617012) and was a “c” in CAC*c*UAUUU of the *H. sapiens* histone H3H mRNA (gi:33873655). In most species, the nucleotide frequency order was U>C≥G at both the poly(A) tail starting position (**Table 3** **)** and U>C>G at the poly(A) tail attachment position (**Table S2,** and **Figures 2** **,** **3** **, and** **4**).

However, even though the mapping of mRNA on the genome sequence is the most accurate approach to date [24], it is still difficult to know which adenosine is the precise location of the poly(A) site when the site is mapped to a multiple-adenosine sequence, regardless of whether the method used is bioinformatics analysis or laboratory conversion of mRNA to cDNA using oligo (dT). In the present case, this bioinformatics study was intended mainly to provide a relative frequency of adenosine at the poly(A) site for the purpose of comparison among species. Further research is required to locate the poly(A) site more precisely for the aligned adenosine poly(A) sites.

The knowledge about poly(A) site type evolution obtained from this large-scale survey of many species and kingdoms could potentially be used to improve poly(A) site prediction software. One such software package for plant poly(A) site prediction was developed from *Arabidopsis* and rice (*Oryza sativa*) poly(A) site data [46], [47]. The findings from the present study regarding the species/kingdoms at the mRNA processing site may be useful as new parameters, in addition to the upstream and downstream motifs, for verifying and improving the accuracy of poly(A) site prediction.

The comparative study (**Figures 2** **,** **3** **,** **4** **, and** **5**) revealed new knowledge that was clearly more than simple UA richness and CA richness at the poly(A) sites. The present study discovered that the A-type and non-A-type poly(A) sites had clear differences in nucleotide composition selection at both the poly(A) tail attachment position and the poly(A) tail starting position (**Figures 2** **,** **4** **, and** **5**). This discovery was achieved through comparing the poly(A) site nucleotide ratios (e.g., C/G, C/U, G/U, etc.) with the same nucleotide ratios of the poly(A) site region of the mRNA sequences.

For the attachment position of non-A-type poly(A) sites, C was strongly preferred over G in plants but not in animals (**Figure 2**), and U was greatly preferred over G in plants, but the opposite was the case in most animals (**Figure 5**). Even though U was more frequent than C at the poly(A) tail attachment position in terms of actual numbers and frequencies (**Table S2**), C was clearly more preferred over U in all plants and most animals if normalized by the C/U ratio of the mRNA (**Figure 4**). Even though C was proportionally over-represented at the poly(A) tail attachment position in comparison with the mRNA nucleotide composition, U was still more frequent overall (**Table 2**). This may have been because U was much more frequent than C in the mRNA. The preference for C over U could not overturn the ratio at the attachment position. Given that both A-type and non-A-type poly(A) sites selected C over U for the poly(A) tail attachment position (in comparison with the mRNA C/U ratios), the finding is much more advanced than the simple existing knowledge that the poly(A) site is usually at UA (or TA for DNA) or CA, because there was no UA or CA at the non-A-type poly(A) sites but C was still preferred at the attachment position.

In contrast, the poly(A) tail starting position favoured U over G in most species (**Figure 5**) and, to a certain extent, C over G in plants (**Figure 2**). When sorted by the C/G ratio for the poly(A) tail attachment position of the non-A-type poly(A) sites, the species clearly belonged to one of three groups: animals, dicot plants, or monocot plants (**Figure 3A**). This grouping according to C/G ratio preferences suggests the involvement of the C/G ratio at the attachment position during evolution of the higher organisms. Further research is required to verify whether the observed difference between dicotyledonous and monocotyledonous plants is relatively universal. This knowledge about the non-A-type poly(A) sites is likely novel, as the nucleotide composition of this group of poly(A) sites has not been reported in the literature.

For the poly(A) tail starting position, U was generally preferred over G (**Figure 5**) This information clearly indicates that the poly(A) tail starting position not only predominantly prefers A but also is not random for other nucleotides. In plants (but not in animals), C was generally preferred over G for both the attachment position and the poly(A) tail starting position (**Figure 2**), suggesting the existence of a specific mechanism operating on the preference for C over G at these two positions in plants.

This large-scale analysis of polyadenylation site evolution revealed nucleotide composition features at both the poly(A) tail attachment position and the starting position of the cleavage sites in both the A-type and the non-A-type poly(A) sites of a wide range of species and kingdoms. Although there was a preference for a CA dinucleotide covering the mapped poly(A) sites and an A at the mapped poly(A) tail starting position in some mammals [18], [20], [48], we detected different dinucleotide preferences in different groups of species as well as the independence of CA for adenosine preference at the poly(A) tail starting position in various species. We found that all 29 analyzed species from various kingdoms preferred adenosine at the poly(A) tail starting position, and we proved statistically that the adenosine preference at the poly(A) site starting position was not a sequence alignment artifact during mapping (**Table 3**). The results revealed the diversity among species and the evolutionary pattern among the kingdoms and pointed to the early emergence of a dominant A-type selection of poly(A) sites in a common ancestor of these kingdoms. The upstream canonical A[A/U]UAAA motif has been confirmed to be one of the major polyadenylation signals in animals [18], [25], [30] and can be used to identify poly(A) sites relatively successfully [24], [26], [27]. In the present study, however, we discovered that both the poly(A) tail attachment position and the starting position have strong selection in nucleotide composition in likely all the 29 analyzed species and therefore cannot be randomly determined and must play an important role in fine-tuning the precise position for poly(A) tailing.

When the poly(A) sites were classified as A-type or non-A-type by whether the poly(A) tail starting position was an adenosine or a non-adenosine, the A-type and non-A-type poly(A) sites were different not only at the poly(A) tail starting position but also in terms of some features at the poly(A) tail attachment position. Interesting also is the level of similarity of the G/U ratios at the attachment position between the two groups of poly(A) sites (**Figure 5**). These findings provide further knowledge about poly(A) site selection, are useful for the prediction of the precise mRNA poly(A) sites, and can assist with further investigation into the molecular mechanism of mRNA processing and polyadenylation.

## Methods

### Analysis of Sequences

We analyzed all the completely sequenced genomes and various incomplete but assembled genomes in NCBI GenBank (http://www.ncbi.nlm.nih.gov) and all mRNA sequences of these species from the NCBI core nucleotide sequence database (http://www.ncbi.nlm.nih.gov/nuccore) (**Table S1** for genome and chromosome ID list). The reason we used all or nearly all the mRNAs of the species in GenBank was to minimize the tissue-specific bias of mRNA and to minimize the artificial poly(A) sites created by internal priming during cDNA synthesis.

### Identification of Polyadenylated mRNA and Unique mRNA

In GenBank, not all the species have poly(A) tails in the mRNA sequence sets, because their poly(A) tails are often trimmed off during sequence cleaning and processing before submission to NCBI. The 3′ end of mRNA sequences from NCBI is not always the poly(A) site, because 3′ truncation is possible. To minimize false poly(A) tailed mRNA, we considered an mRNA transcript polyadenylated only if it met the following three criteria: 1) the mRNA sequence upstream of the poly(A) tail must have at least 100 bases and have no N’s; 2) the mRNA has a poly(A) tail at the 3′ end; and 3) the pure poly(A) tail must have at least 12 A’s. In this study, after screening all or most genomes, we focused our comparative characterization on the species with a sufficiently large number of mapped poly(A) sites for quantitative comparison among species. Consequently, 29 species were retained after this screening, namely 2 fungi, 2 protozoan protists, 18 animals, and 7 plants (**Table 1** for list of species and common names, and **Table S1** for genome and chromosome ID list). Fungi and protozoan parasites were included as representatives of their kingdoms in this comparison even though those organisms have a much smaller number of poly(A) sites mapped to their genomes in comparison with the plant and animal species (**Table S3**). We screened the polyadenylated mRNA sequences using the 100–nucleotide region directly in attachment with the poly(A) tail and eliminated the duplicated poly(A) sequences. In this way, each poly(A) site 100–base sequence that remained was unique.

### Mapping and Analysis of Poly(A) Sites

We aligned these 100–nucleotide unique mRNA sequences to the genome sequences of their corresponding species. The alignment was done with zero tolerance for mismatches. The mapping narrowed the polyadenylation site to a single genomic or pre-mRNA nucleotide corresponding to the first A of the mRNA poly(A) tail. A pre-mRNA 100–nucleotide sequence downstream of the poly(A) site was inferred from the mapped region of the genomic sequence. We focused our study on the two nucleotides directly beside the candidate cleavage bond: the poly(A) tail attachment position (or −1 position; the position that is upstream of the cleavage bond), and the starting position (or +1 position; the position that is downstream of the bond). Therefore, for each mapped poly(A) site, we identified the following 201 nucleotides: the upstream 99–nucleotide sequence (without the attachment position), the poly(A) tail attachment nucleotide, the poly(A) tail starting nucleotide, and the downstream 100–nucleotide sequence.

For the purpose of comparing the nucleotide compositions at the poly(A) sites, we also analyzed the mRNA nucleotide composition for the 99 bases (excluding the nucleotide at the attachment position) and 100 bases (including the nucleotide at the attachment position) of mRNA directly upstream of the poly(A) sites. These two upstream segments overlapped and were different by only one nucleotide [the poly(A) tail attachment position]. For the calculation of the random model theoretical percentage of A of the poly(A) tail starting position in **Table 3**, we used the adenosine sequence (i.e., the 100 bases) upstream of that starting position. However, for the comparison of base composition between the poly(A) tail attachment position and the starting position (**Figures 2** **,** **3** **,** **4** **, and** **5**), this 100–base sequence was not very suitable for representing the mRNA base composition in the poly(A) site region, because the attachment position was the last nucleotide of the 100–base sequence but the starting position was not. Therefore, for the estimation of the mRNA base composition in the poly(A) site region in **Figures 2** **to** **5**, we used the 99–base sequence, which is the portion remaining after the attachment position was excluded from the 100 bases. In addition to the analysis of the mapped sites of all mRNAs, we also separately analyzed only the mRNAs that have a pre-mRNA non-adenosine nucleotide replaced by the poly(A) tail. This is because we wanted to investigate the similarity and differences between the two groups of poly(A) sites.

Most of the analyses used sequence data from all mapped locations from each unique mRNA. If some species were particularly rich in A’s immediately after poly(A) sites (usually as a result of multiple-copy genes), we also analyzed unique poly(A) sites by using only one poly(A) site sequence to represent all the poly(A) site regions that are identical in the 100 bases immediately upstream of the poly(A) tail starting position.

This study involved heavy computation (approximately 75 GB of data, and running of programs for about two months) assisted by Perl scripts. Two computer servers (a Linux server and a Windows server) were used to verify each other for the sequence screening and mapping results.

### Random Model Estimation of A-type Poly(A) Site Frequency from mRNA–genome Alignment

The theoretical frequency of A-type poly(A) sites from the alignment in the random model is *p*+*p*(1−*p*) = *p*(2−*p*), where (1−*p*) is the non-A nucleotide content. This means “the percentage of A in mRNA” plus “the frequency of A at the position adjacent to the non-A-type poly(A) site”. If the A nucleotide percentage in mRNA is 30%, the A-type poly(A) site from the alignment will be 30%+[30%(100%−30%)] = 51%, where (100%−30%) is the non-A nucleotide content. The multiple-A or multiple-non-A sequences do not alter the A-type or non-A-type poly(A) site probability in this random model, because both A and non-A have a random chance in this aspect within their nucleotide content ranges. The genomic frequency of adenosine at the poly(A) site is tested against the adenosine frequency of mRNA nucleotide composition using the chi-square test (See **File S1** for details).

## Statistics

The test between the observed nucleotide numbers in the alignment and the numbers in the random model was carried out using the chi-square test. The nucleotide ratio tendency comparison between mRNA and poly(A) sites was carried out by correlation and linear regression analyses using the statistical package of Excel 2010.

## Supporting Information

Table S1 **Genome and chromosome ID list.**
(DOC)

Table S2 **Proportion shares among U, C, and G for the polyadenylation [poly(A)] tail attachment nucleotides for the transcripts that have a pre–messenger RNA non-adenosine nucleotide replaced by the poly(A) tail.**
(DOCX)

Table S3 **Pre–messenger RNA (mRNA) adenosine replaced by the polyadenylation [poly(A)] tail–normalized frequency with internal priming estimation.**
(DOCX)

File S1 **Estimation of the theoretical A-type polyadenylation [poly(A)] site frequency in the random model.**
(DOCX)
