# Supplementary material for: RNA Polyadenylation Sites on the Genomes of Microorganisms, Animals, and Plants
Source: PLoS One. 2013 Nov 18;8(11):e79511. doi: 10.1371/journal.pone.0079511 (PMC3832601; doi:10.1371/journal.pone.0079511)
Supplement: Table S1 — Genome and chromosome ID list. (DOC) [file pone.0079511.s001.doc]

**Table S1.** Genome and chromosome ID list

| Species Name | | chromosome ID | | |
| --- | --- | --- | --- | --- |
| Anopheles_gambiae | | >NT_078265| Anopheles gambiae str. PEST chromosome 2L, whole genome shotgun | | |
| Anopheles_gambiae | | >NT_078266|Anopheles gambiae str. PEST chromosome 2R, whole genome shotgun | | |
| Anopheles_gambiae | | >NT_078267| Anopheles gambiae str. PEST chromosome 3L, whole genome shotgun | | |
| Anopheles_gambiae | | >NT_078268| Anopheles gambiae str. PEST chromosome 3R, whole genome shotgun | | |
| Anopheles_gambiae | | >NC_004818| Anopheles gambiae str. PEST chromosome X, whole genome shotgun | | |
| Anopheles_gambiae | | >NC_002084| Anopheles gambiae mitochondrion, complete genome. | | |
| Apis_mellifera | | >gi|110825701|ref|NC_007070.2|NC_007070 Apis mellifera linkage group 1, reference assembly (based on Amel_4.0), whole genome shotgun sequence | | |
| Apis_mellifera | | >gi|110825711|ref|NC_007079.2|NC_007079 Apis mellifera linkage group 10, reference assembly (based on Amel_4.0), whole genome shotgun sequence | | |
| Apis_mellifera | | >gi|110825732|ref|NC_007080.2|NC_007080 Apis mellifera linkage group 11, reference assembly (based on Amel_4.0), whole genome shotgun sequence | | |
| Apis_mellifera | | >gi|110825763|ref|NC_007083.2|NC_007083 Apis mellifera linkage group 14, reference assembly (based on Amel_4.0), whole genome shotgun sequence | | |
| Apis_mellifera | | >gi|110825764|ref|NC_007084.2|NC_007084 Apis mellifera linkage group 15, reference assembly (based on Amel_4.0), whole genome shotgun sequence | | |
| Apis_mellifera | | >gi|110825765|ref|NC_007085.2|NC_007085 Apis mellifera linkage group 16, reference assembly (based on Amel_4.0), whole genome shotgun sequence | | |
| Apis_mellifera | | >gi|110825766|ref|NC_007071.2|NC_007071 Apis mellifera linkage group 2, reference assembly (based on Amel_4.0), whole genome shotgun sequence | | |
| Apis_mellifera | | >gi|110825767|ref|NC_007072.2|NC_007072 Apis mellifera linkage group 3, reference assembly (based on Amel_4.0), whole genome shotgun sequence | | |
| Apis_mellifera | | >gi|110825768|ref|NC_007073.2|NC_007073 Apis mellifera linkage group 4, reference assembly (based on Amel_4.0), whole genome shotgun sequence | | |
| Apis_mellifera | | >gi|110825769|ref|NC_007074.2|NC_007074 Apis mellifera linkage group 5, reference assembly (based on Amel_4.0), whole genome shotgun sequence | | |
| Apis_mellifera | | >gi|110825770|ref|NC_007075.2|NC_007075 Apis mellifera linkage group 6, reference assembly (based on Amel_4.0), whole genome shotgun sequence | | |
| Apis_mellifera | | >gi|110825771|ref|NC_007076.2|NC_007076 Apis mellifera linkage group 7, reference assembly (based on Amel_4.0), whole genome shotgun sequence | | |
| Apis_mellifera | | >gi|110825772|ref|NC_007077.2|NC_007077 Apis mellifera linkage group 8, reference assembly (based on Amel_4.0), whole genome shotgun sequence | | |
| Apis_mellifera | | >gi|110825774|ref|NC_007078.2|NC_007078 Apis mellifera linkage group 9, reference assembly (based on Amel_4.0), whole genome shotgun sequence | | |
| Arabidopsis_thaliana | | >ref|NC_003070.9|:1-30427671 Arabidopsis thaliana chromosome 1, complete sequence | | |
| Arabidopsis_thaliana | | >ref|NC_003071.7|:1-19698289 Arabidopsis thaliana chromosome 2, complete sequence | | |
| Arabidopsis_thaliana | | >ref|NC_003074.8|:1-23459830 Arabidopsis thaliana chromosome 3, complete sequence | | |
| Arabidopsis_thaliana | | >ref|NC_003075.7|:1-18585056 Arabidopsis thaliana chromosome 4, complete sequence | | |
| Arabidopsis_thaliana | | >ref|NC_003076.8|:1-26975502 Arabidopsis thaliana chromosome 5, complete sequence | | |
| Aspergillus_nidulans | | >Aspergillus nidulans FGSC A4 scaffold_27, whole genome shotgun | | |
| Aspergillus_nidulans | | >Aspergillus nidulans FGSC A4 chromosome VIII scaffold_16, whole | | |
| Aspergillus_nidulans | | >Aspergillus nidulans FGSC A4 chromosome IV scaffold_15, whole | | |
| Aspergillus_nidulans | | >Aspergillus nidulans FGSC A4 chromosome VII scaffold_14, whole | | |
| Aspergillus_nidulans | | >Aspergillus nidulans FGSC A4 chromosome III scaffold_13, whole | | |
| Aspergillus_nidulans | | >Aspergillus nidulans FGSC A4 scaffold_12, whole genome shotgun | | |
| Aspergillus_nidulans | | >Aspergillus nidulans FGSC A4 chromosome II scaffold_11, whole | | |
| Aspergillus_nidulans | | >Aspergillus nidulans FGSC A4 chromosome IV scaffold_10, whole | | |
| Aspergillus_nidulans | | >Aspergillus nidulans FGSC A4 chromosome IV scaffold_9, whole genome | | |
| Aspergillus_nidulans | | >Aspergillus nidulans FGSC A4 chromosome I scaffold_8, whole genome | | |
| Aspergillus_nidulans | | >Aspergillus nidulans FGSC A4 chromosome I scaffold_7, whole genome | | |
| Aspergillus_nidulans | | >Aspergillus nidulans FGSC A4 chromosome V scaffold_6, whole genome | | |
| Aspergillus_nidulans | | >Aspergillus nidulans FGSC A4 chromosome III scaffold_5, whole | | |
| Aspergillus_nidulans | | >Aspergillus nidulans FGSC A4 chromosome II scaffold_4, whole genome | | |
| Aspergillus_nidulans | | >Aspergillus nidulans FGSC A4 chromosome VI scaffold_3, whole genome | | |
| Aspergillus_nidulans | | >Aspergillus nidulans FGSC A4 chromosome VII scaffold_2, whole | | |
| Aspergillus_nidulans | | >Aspergillus nidulans FGSC A4 chromosome VIII scaffold_1, whole | | |
| Bos_taurus | | >gi|257481614|tpg|GK000001.2| TPA: Bos taurus chromosome 1, whole genome shotgun sequence | | |
| Bos_taurus | | >gi|257481335|tpg|GK000010.2| TPA: Bos taurus chromosome 10, whole genome shotgun sequence | | |
| Bos_taurus | | >gi|257481612|tpg|GK000003.2| TPA: Bos taurus chromosome 3, whole genome shotgun sequence | | |
| Bos_taurus | | >gi|257481341|tpg|GK000004.2| TPA: Bos taurus chromosome 4, whole genome shotgun sequence | | |
| Bos_taurus | | >gi|257481340|tpg|GK000005.2| TPA: Bos taurus chromosome 5, whole genome shotgun sequence | | |
| Bos_taurus | | >gi|257481339|tpg|GK000006.2| TPA: Bos taurus chromosome 6, whole genome shotgun sequence | | |
| Bos_taurus | | >gi|257481338|tpg|GK000007.2| TPA: Bos taurus chromosome 7, whole genome shotgun sequence | | |
| Bos_taurus | | >gi|257481337|tpg|GK000008.2| TPA: Bos taurus chromosome 8, whole genome shotgun sequence | | |
| Bos_taurus | | >gi|257481336|tpg|GK000009.2| TPA: Bos taurus chromosome 9, whole genome shotgun sequence | | |
| Bos_taurus | |  | | |
| Brachypodium_distachyon | | >gi|289163340|gb|CM000880.1| Brachypodium distachyon strain Bd21 chromosome 1, whole genome shotgun sequence | | |
| Brachypodium_distachyon | | >gi|289163332|gb|GG745432.1| Brachypodium distachyon strain Bd21 unplaced genomic scaffold BRADIscaffold_10, whole genome shotgun sequence | | |
| Brachypodium_distachyon | | >gi|289163331|gb|GG745433.1| Brachypodium distachyon strain Bd21 unplaced genomic scaffold BRADIscaffold_11, whole genome shotgun sequence | | |
| Brachypodium_distachyon | | >gi|289163339|gb|CM000881.1| Brachypodium distachyon strain Bd21 chromosome 2, whole genome shotgun sequence | | |
| Brachypodium_distachyon | | >gi|289163338|gb|CM000882.1| Brachypodium distachyon strain Bd21 chromosome 3, whole genome shotgun sequence | | |
| Brachypodium_distachyon | | >gi|289163337|gb|CM000883.1| Brachypodium distachyon strain Bd21 chromosome 4, whole genome shotgun sequence | | |
| Brachypodium_distachyon | | >gi|289163336|gb|CM000884.1| Brachypodium distachyon strain Bd21 chromosome 5, whole genome shotgun sequence | | |
| Brachypodium_distachyon | | >gi|289163335|gb|GG745429.1| Brachypodium distachyon strain Bd21 unplaced genomic scaffold BRADIscaffold_7, whole genome shotgun sequence | | |
| Brachypodium_distachyon | | >gi|289163334|gb|GG745430.1| Brachypodium distachyon strain Bd21 unplaced genomic scaffold BRADIscaffold_8, whole genome shotgun sequence | | |
| Brachypodium_distachyon | | >gi|289163333|gb|GG745431.1| Brachypodium distachyon strain Bd21 unplaced genomic scaffold BRADIscaffold_9, whole genome shotgun sequence | | |
| Caenorhabditis_elegans | | >gi|67514031|emb|BX284601.3| Caenorhabditis elegans chromosome I, complete sequence | | |
| Caenorhabditis_elegans | | >gi|67514032|emb|BX284602.3| Caenorhabditis elegans chromosome II, complete sequence | | |
| Caenorhabditis_elegans | | >gi|67514029|emb|BX284603.2| Caenorhabditis elegans chromosome III, complete sequence | | |
| Caenorhabditis_elegans | | >gi|67514030|emb|BX284604.2| Caenorhabditis elegans chromosome IV, complete sequence | | |
| Caenorhabditis_elegans | | >gi|67514033|emb|BX284605.3| Caenorhabditis elegans chromosome V, complete sequence | | |
| Caenorhabditis_elegans | | >gi|67424289|emb|BX284606.3| Caenorhabditis elegans chromosome X, complete sequence | | |
| Callithrix_jacchus | | >gi|290467399|ref|NC_013905.1| Callithrix jacchus chromosome 10, Callithrix jacchus-3.2, whole genome shotgun sequence | | |
| Callithrix_jacchus | | >gi|290467398|ref|NC_013906.1| Callithrix jacchus chromosome 11, Callithrix jacchus-3.2, whole genome shotgun sequence | | |
| Callithrix_jacchus | | >gi|290467397|ref|NC_013907.1| Callithrix jacchus chromosome 12, Callithrix jacchus-3.2, whole genome shotgun sequence | | |
| Callithrix_jacchus | | >gi|290467396|ref|NC_013908.1| Callithrix jacchus chromosome 13, Callithrix jacchus-3.2, whole genome shotgun sequence | | |
| Callithrix_jacchus | | >gi|290467395|ref|NC_013909.1| Callithrix jacchus chromosome 14, Callithrix jacchus-3.2, whole genome shotgun sequence | | |
| Callithrix_jacchus | | >gi|290467394|ref|NC_013910.1| Callithrix jacchus chromosome 15, Callithrix jacchus-3.2, whole genome shotgun sequence | | |
| Callithrix_jacchus | | >gi|290467393|ref|NC_013911.1| Callithrix jacchus chromosome 16, Callithrix jacchus-3.2, whole genome shotgun sequence | | |
| Callithrix_jacchus | | >gi|290467392|ref|NC_013912.1| Callithrix jacchus chromosome 17, Callithrix jacchus-3.2, whole genome shotgun sequence | | |
| Callithrix_jacchus | | >gi|290467391|ref|NC_013913.1| Callithrix jacchus chromosome 18, Callithrix jacchus-3.2, whole genome shotgun sequence | | |
| Callithrix_jacchus | | >gi|290467390|ref|NC_013914.1| Callithrix jacchus chromosome 19, Callithrix jacchus-3.2, whole genome shotgun sequence | | |
| Callithrix_jacchus | | >gi|290467389|ref|NC_013915.1| Callithrix jacchus chromosome 20, Callithrix jacchus-3.2, whole genome shotgun sequence | | |
| Callithrix_jacchus | | >gi|290467388|ref|NC_013916.1| Callithrix jacchus chromosome 21, Callithrix jacchus-3.2, whole genome shotgun sequence | | |
| Callithrix_jacchus | | >gi|290467387|ref|NC_013917.1| Callithrix jacchus chromosome 22, Callithrix jacchus-3.2, whole genome shotgun sequence | | |
| Callithrix_jacchus | | >gi|290467405|ref|NC_013899.1| Callithrix jacchus chromosome 4, Callithrix jacchus-3.2, whole genome shotgun sequence | | |
| Callithrix_jacchus | | >gi|290467404|ref|NC_013900.1| Callithrix jacchus chromosome 5, Callithrix jacchus-3.2, whole genome shotgun sequence | | |
| Callithrix_jacchus | | >gi|290467403|ref|NC_013901.1| Callithrix jacchus chromosome 6, Callithrix jacchus-3.2, whole genome shotgun sequence | | |
| Callithrix_jacchus | | >gi|290467402|ref|NC_013902.1| Callithrix jacchus chromosome 7, Callithrix jacchus-3.2, whole genome shotgun sequence | | |
| Callithrix_jacchus | | >gi|290467401|ref|NC_013903.1| Callithrix jacchus chromosome 8, Callithrix jacchus-3.2, whole genome shotgun sequence | | |
| Callithrix_jacchus | | >gi|290467400|ref|NC_013904.1| Callithrix jacchus chromosome 9, Callithrix jacchus-3.2, whole genome shotgun sequence | | |
| Callithrix_jacchus | | >gi|290467386|ref|NC_013918.1| Callithrix jacchus chromosome X, Callithrix jacchus-3.2, whole genome shotgun sequence | | |
| Callithrix_jacchus | | >gi|290467385|ref|NC_013919.1| Callithrix jacchus chromosome Y, Callithrix jacchus-3.2, whole genome shotgun sequence | | |
| Callithrix_jacchus | | >gi|289734502|ref|NW_003187718.1| Callithrix jacchus chromosome Y unlocalized genomic scaffold, Callithrix jacchus-3.2 CJAY_random_049, whole genome shotgun sequence | | |
| Callithrix_jacchus | | >gi|289722669|ref|NW_003199274.1| Callithrix jacchus unplaced genomic scaffold, Callithrix jacchus-3.2 CJAUN_random_11556, whole genome shotgun sequence | | |
| Canis_lupus_familiaris | | >gi|70724219|gb|CM000001.2| Canis familiaris chromosome 1, whole genome shotgun sequence | | |
| Canis_lupus_familiaris | | >gi|70724210|gb|CM000010.2| Canis familiaris chromosome 10, whole genome shotgun sequence | | |
| Canis_lupus_familiaris | | >gi|70724218|gb|CM000002.2| Canis familiaris chromosome 2, whole genome shotgun sequence | | |
| Canis_lupus_familiaris | | >gi|70724216|gb|CM000004.2| Canis familiaris chromosome 4, whole genome shotgun sequence | | |
| Canis_lupus_familiaris | | >gi|70724215|gb|CM000005.2| Canis familiaris chromosome 5, whole genome shotgun sequence | | |
| Canis_lupus_familiaris | | >gi|70724214|gb|CM000006.2| Canis familiaris chromosome 6, whole genome shotgun sequence | | |
| Canis_lupus_familiaris | | >gi|70724213|gb|CM000007.2| Canis familiaris chromosome 7, whole genome shotgun sequence | | |
| Canis_lupus_familiaris | | >gi|70724212|gb|CM000008.2| Canis familiaris chromosome 8, whole genome shotgun sequence | | |
| Canis_lupus_familiaris | | >gi|70724211|gb|CM000009.2| Canis familiaris chromosome 9, whole genome shotgun sequence | | |
| Canis_lupus_familiaris | | >gi|70724181|gb|CM000039.2| Canis familiaris chromosome X, whole genome shotgun sequence | | |
| Ciona_intestinalis | | >gi|48860548|ref|NC_004447.2| Ciona intestinalis mitochondrion, complete genome | | |
| Ciona_intestinalis | | >gi|193885917|ref|NW_001955232.1|CinUn_WGA226_1 Ciona intestinalis genomic contig, reference assembly (based on Ciona_intestinalis_v1.0 ciona2276) | | |
| Danio_rerio | | >gi|15079186|ref|NC_002333.2| Danio rerio mitochondrion, complete genome | | |
| Danio_rerio | | >gi|258456220|ref|NC_007112.4| Danio rerio strain Tuebingen chromosome 1, Zv8 | | |
| Danio_rerio | | >gi|258456211|ref|NC_007121.4| Danio rerio strain Tuebingen chromosome 10, Zv8 | | |
| Danio_rerio | | >gi|258456210|ref|NC_007122.4| Danio rerio strain Tuebingen chromosome 11, Zv8 | | |
| Danio_rerio | | >gi|258456209|ref|NC_007123.4| Danio rerio strain Tuebingen chromosome 12, Zv8 | | |
| Danio_rerio | | >gi|258456208|ref|NC_007124.4| Danio rerio strain Tuebingen chromosome 13, Zv8 | | |
| Danio_rerio | | >gi|258456207|ref|NC_007125.4| Danio rerio strain Tuebingen chromosome 14, Zv8 | | |
| Danio_rerio | | >gi|258456206|ref|NC_007126.4| Danio rerio strain Tuebingen chromosome 15, Zv8 | | |
| Danio_rerio | | >gi|258456205|ref|NC_007127.4| Danio rerio strain Tuebingen chromosome 16, Zv8 | | |
| Danio_rerio | | >gi|258456204|ref|NC_007128.4| Danio rerio strain Tuebingen chromosome 17, Zv8 | | |
| Danio_rerio | | >gi|258456203|ref|NC_007129.4| Danio rerio strain Tuebingen chromosome 18, Zv8 | | |
| Danio_rerio | | >gi|258456202|ref|NC_007130.4| Danio rerio strain Tuebingen chromosome 19, Zv8 | | |
| Danio_rerio | | >gi|258456219|ref|NC_007113.4| Danio rerio strain Tuebingen chromosome 2, Zv8 | | |
| Danio_rerio | | >gi|258456201|ref|NC_007131.4| Danio rerio strain Tuebingen chromosome 20, Zv8 | | |
| Danio_rerio | | >gi|258456200|ref|NC_007132.4| Danio rerio strain Tuebingen chromosome 21, Zv8 | | |
| Danio_rerio | | >gi|258456199|ref|NC_007133.4| Danio rerio strain Tuebingen chromosome 22, Zv8 | | |
| Danio_rerio | | >gi|258456198|ref|NC_007134.4| Danio rerio strain Tuebingen chromosome 23, Zv8 | | |
| Danio_rerio | | >gi|258456197|ref|NC_007135.4| Danio rerio strain Tuebingen chromosome 24, Zv8 | | |
| Danio_rerio | | >gi|258456196|ref|NC_007136.4| Danio rerio strain Tuebingen chromosome 25, Zv8 | | |
| Danio_rerio | | >gi|258456218|ref|NC_007114.4| Danio rerio strain Tuebingen chromosome 3, Zv8 | | |
| Danio_rerio | | >gi|258456217|ref|NC_007115.4| Danio rerio strain Tuebingen chromosome 4, Zv8 | | |
| Danio_rerio | | >gi|258456216|ref|NC_007116.4| Danio rerio strain Tuebingen chromosome 5, Zv8 | | |
| Danio_rerio | | >gi|258456215|ref|NC_007117.4| Danio rerio strain Tuebingen chromosome 6, Zv8 | | |
| Danio_rerio | | >gi|258456214|ref|NC_007118.4| Danio rerio strain Tuebingen chromosome 7, Zv8 | | |
| Danio_rerio | | >gi|258456213|ref|NC_007119.4| Danio rerio strain Tuebingen chromosome 8, Zv8 | | |
| Danio_rerio | | >gi|258456212|ref|NC_007120.4| Danio rerio strain Tuebingen chromosome 9, Zv8 | | |
| Danio_rerio | | >gi|258425119|ref|NW_003052456.1| Danio rerio strain Tuebingen unplaced genomic scaffold, Zv8_NA4098 | | |
| Drosophila_melanogaster | | >ref|NT_033779.4|:1-23011544 Drosophila melanogaster chromosome 2L, complete sequence | | |
| Drosophila_melanogaster | | >ref|NT_033778.3|:1-21146708 Drosophila melanogaster chromosome 2R, complete sequence | | |
| Drosophila_melanogaster | | >ref|NT_037436.3|:1-24543557 Drosophila melanogaster chromosome 3L, complete sequence | | |
| Drosophila_melanogaster | | >ref|NT_033777.2|:1-27905053 Drosophila melanogaster chromosome 3R, complete sequence | | |
| Drosophila_melanogaster | | >ref|NC_004353.3|:1-1351857 Drosophila melanogaster chromosome 4, complete sequence | | |
| Drosophila_melanogaster | | >ref|NC_004354.3|:1-22422827 Drosophila melanogaster chromosome X, complete sequence | | |
| Equus_caballus | | >gi|194246357|ref|NC_009144.2|NC_009144 Equus caballus chromosome 1, reference assembly (based on EquCab2), whole genome shotgun sequence | | |
| Equus_caballus | | >gi|194246358|ref|NC_009153.2|NC_009153 Equus caballus chromosome 10, reference assembly (based on EquCab2), whole genome shotgun sequence | | |
| Equus_caballus | | >gi|194246359|ref|NC_009154.2|NC_009154 Equus caballus chromosome 11, reference assembly (based on EquCab2), whole genome shotgun sequence | | |
| Equus_caballus | | >gi|194246360|ref|NC_009155.2|NC_009155 Equus caballus chromosome 12, reference assembly (based on EquCab2), whole genome shotgun sequence | | |
| Equus_caballus | | >gi|194246361|ref|NC_009156.2|NC_009156 Equus caballus chromosome 13, reference assembly (based on EquCab2), whole genome shotgun sequence | | |
| Equus_caballus | | >gi|194246362|ref|NC_009157.2|NC_009157 Equus caballus chromosome 14, reference assembly (based on EquCab2), whole genome shotgun sequence | | |
| Equus_caballus | | >gi|194246363|ref|NC_009158.2|NC_009158 Equus caballus chromosome 15, reference assembly (based on EquCab2), whole genome shotgun sequence | | |
| Equus_caballus | | >gi|194246364|ref|NC_009159.2|NC_009159 Equus caballus chromosome 16, reference assembly (based on EquCab2), whole genome shotgun sequence | | |
| Equus_caballus | | >gi|194246365|ref|NC_009160.2|NC_009160 Equus caballus chromosome 17, reference assembly (based on EquCab2), whole genome shotgun sequence | | |
| Equus_caballus | | >gi|194246366|ref|NC_009161.2|NC_009161 Equus caballus chromosome 18, reference assembly (based on EquCab2), whole genome shotgun sequence | | |
| Equus_caballus | | >gi|194246370|ref|NC_009162.2|NC_009162 Equus caballus chromosome 19, reference assembly (based on EquCab2), whole genome shotgun sequence | | |
| Equus_caballus | | >gi|194246371|ref|NC_009145.2|NC_009145 Equus caballus chromosome 2, reference assembly (based on EquCab2), whole genome shotgun sequence | | |
| Equus_caballus | | >gi|194246372|ref|NC_009163.2|NC_009163 Equus caballus chromosome 20, reference assembly (based on EquCab2), whole genome shotgun sequence | | |
| Equus_caballus | | >gi|194246373|ref|NC_009164.2|NC_009164 Equus caballus chromosome 21, reference assembly (based on EquCab2), whole genome shotgun sequence | | |
| Equus_caballus | | >gi|194246374|ref|NC_009165.2|NC_009165 Equus caballus chromosome 22, reference assembly (based on EquCab2), whole genome shotgun sequence | | |
| Equus_caballus | | >gi|194246375|ref|NC_009166.2|NC_009166 Equus caballus chromosome 23, reference assembly (based on EquCab2), whole genome shotgun sequence | | |
| Equus_caballus | | >gi|194246376|ref|NC_009167.2|NC_009167 Equus caballus chromosome 24, reference assembly (based on EquCab2), whole genome shotgun sequence | | |
| Equus_caballus | | >gi|194246377|ref|NC_009168.2|NC_009168 Equus caballus chromosome 25, reference assembly (based on EquCab2), whole genome shotgun sequence | | |
| Equus_caballus | | >gi|194246378|ref|NC_009169.2|NC_009169 Equus caballus chromosome 26, reference assembly (based on EquCab2), whole genome shotgun sequence | | |
| Equus_caballus | | >gi|194246379|ref|NC_009170.2|NC_009170 Equus caballus chromosome 27, reference assembly (based on EquCab2), whole genome shotgun sequence | | |
| Equus_caballus | | >gi|194246380|ref|NC_009171.2|NC_009171 Equus caballus chromosome 28, reference assembly (based on EquCab2), whole genome shotgun sequence | | |
| Equus_caballus | | >gi|194246381|ref|NC_009172.2|NC_009172 Equus caballus chromosome 29, reference assembly (based on EquCab2), whole genome shotgun sequence | | |
| Equus_caballus | | >gi|194246382|ref|NC_009146.2|NC_009146 Equus caballus chromosome 3, reference assembly (based on EquCab2), whole genome shotgun sequence | | |
| Equus_caballus | | >gi|194246383|ref|NC_009173.2|NC_009173 Equus caballus chromosome 30, reference assembly (based on EquCab2), whole genome shotgun sequence | | |
| Equus_caballus | | >gi|194246384|ref|NC_009174.2|NC_009174 Equus caballus chromosome 31, reference assembly (based on EquCab2), whole genome shotgun sequence | | |
| Equus_caballus | | >gi|194246385|ref|NC_009147.2|NC_009147 Equus caballus chromosome 4, reference assembly (based on EquCab2), whole genome shotgun sequence | | |
| Equus_caballus | | >gi|194246386|ref|NC_009148.2|NC_009148 Equus caballus chromosome 5, reference assembly (based on EquCab2), whole genome shotgun sequence | | |
| Equus_caballus | | >gi|194246387|ref|NC_009149.2|NC_009149 Equus caballus chromosome 6, reference assembly (based on EquCab2), whole genome shotgun sequence | | |
| Equus_caballus | | >gi|194246388|ref|NC_009150.2|NC_009150 Equus caballus chromosome 7, reference assembly (based on EquCab2), whole genome shotgun sequence | | |
| Equus_caballus | | >gi|194246389|ref|NC_009151.2|NC_009151 Equus caballus chromosome 8, reference assembly (based on EquCab2), whole genome shotgun sequence | | |
| Equus_caballus | | >gi|194246401|ref|NC_009152.2|NC_009152 Equus caballus chromosome 9, reference assembly (based on EquCab2), whole genome shotgun sequence | | |
| Equus_caballus | | >gi|194246402|ref|NC_009175.2|NC_009175 Equus caballus chromosome X, reference assembly (based on EquCab2), whole genome shotgun sequence | | |
| Felis_catus | | >gi|224998174|gb|CM000695.1| Felis catus breed mixed chromosome A1, whole genome shotgun sequence | | |
| Felis_catus | | >gi|224998172|gb|CM000697.1| Felis catus breed mixed chromosome A3, whole genome shotgun sequence | | |
| Felis_catus | | >gi|224998171|gb|CM000698.1| Felis catus breed mixed chromosome B1, whole genome shotgun sequence | | |
| Felis_catus | | >gi|224998170|gb|CM000699.1| Felis catus breed mixed chromosome B2, whole genome shotgun sequence | | |
| Felis_catus | | >gi|224998169|gb|CM000700.1| Felis catus breed mixed chromosome B3, whole genome shotgun sequence | | |
| Felis_catus | | >gi|224998168|gb|CM000701.1| Felis catus breed mixed chromosome B4, whole genome shotgun sequence | | |
| Felis_catus | | >gi|224998167|gb|CM000702.1| Felis catus breed mixed chromosome C1, whole genome shotgun sequence | | |
| Felis_catus | | >gi|224998166|gb|CM000703.1| Felis catus breed mixed chromosome C2, whole genome shotgun sequence | | |
| Felis_catus | | >gi|224998161|gb|CM000708.1| Felis catus breed mixed chromosome E1, whole genome shotgun sequence | | |
| Felis_catus | | >gi|224998158|gb|CM000711.1| Felis catus breed mixed chromosome F1, whole genome shotgun sequence | | |
| Felis_catus | | >gi|224998156|gb|CM000713.1| Felis catus breed mixed chromosome X, whole genome shotgun sequence | | |
| Fungi_Neurospora_crassa_uid132 | | >gi|194294104|gb|EU815636.1| Neurospora crass chromosome LGVII translocation T(AR173) breakpoint sequence | | |
| Fungi_Phytophthora_infestans | | >gi|48249219|gb|AY561505.1| Phytophthora infestans transposon hAT-like transposable element DodoPi-5, partial sequence | | |
| Gallus_gallus | | >gi|117306184|gb|CM000093.2| Gallus gallus chromosome 1, whole genome shotgun sequence | | |
| Gallus_gallus | | >gi|117306152|gb|CM000102.2| Gallus gallus chromosome 10, whole genome shotgun sequence | | |
| Gallus_gallus | | >gi|117306183|gb|CM000094.2| Gallus gallus chromosome 2, whole genome shotgun sequence | | |
| Gallus_gallus | | >gi|117306159|gb|CM000095.2| Gallus gallus chromosome 3, whole genome shotgun sequence | | |
| Gallus_gallus | | >gi|117306158|gb|CM000096.2| Gallus gallus chromosome 4, whole genome shotgun sequence | | |
| Gallus_gallus | | >gi|117306157|gb|CM000097.2| Gallus gallus chromosome 5, whole genome shotgun sequence | | |
| Gallus_gallus | | >gi|117306156|gb|CM000098.2| Gallus gallus chromosome 6, whole genome shotgun sequence | | |
| Gallus_gallus | | >gi|117306155|gb|CM000099.2| Gallus gallus chromosome 7, whole genome shotgun sequence | | |
| Gallus_gallus | | >gi|117306154|gb|CM000100.2| Gallus gallus chromosome 8, whole genome shotgun sequence | | |
| Gallus_gallus | | >gi|117306153|gb|CM000101.2| Gallus gallus chromosome 9, whole genome shotgun sequence | | |
| Gallus_gallus | | >gi|117306007|gb|CM000121.2| Gallus gallus chromosome W, whole genome shotgun sequence | | |
| Gallus_gallus | | >gi|117306003|gb|CM000122.2| Gallus gallus chromosome Z, whole genome shotgun sequence | | |
| Homo__sapiens | | >ref|NT_008705.16|:1-39094935 Homo sapiens chromosome 10 genomic contig, GRCh37 reference primary assembly | | |
| Homo__sapiens | | >ref|NT_033985.7|:1-4072029 Homo sapiens chromosome 10 genomic contig, GRCh37 reference primary assembly | | |
| Homo__sapiens | | >ref|NT_031847.8|:1-952205 Homo sapiens chromosome 10 genomic contig, GRCh37 reference primary assembly | | |
| Homo__sapiens | | >ref|NT_077570.1|:1-263307 Homo sapiens chromosome 10 genomic contig, GRCh37 reference primary assembly | | |
| Homo__sapiens | | >ref|NT_077571.1|:1-163231 Homo sapiens chromosome 10 genomic contig, GRCh37 reference primary assembly | | |
| Homo__sapiens | | >ref|NT_030772.10|:1-989829 Homo sapiens chromosome 10 genomic contig, GRCh37 reference primary assembly | | |
| Homo__sapiens | | >ref|NT_030059.13|:1-79420533 Homo sapiens chromosome 10 genomic contig, GRCh37 reference primary assembly | | |
| Homo__sapiens | | >ref|NT_008818.16|:1-6758678 Homo sapiens chromosome 10 genomic contig, GRCh37 reference primary assembly | | |
| Homo__sapiens | | >ref|NT_009237.18|:1-50723853 Homo sapiens chromosome 11 genomic contig, GRCh37 reference primary assembly | | |
| Homo__sapiens | | >ref|NT_035158.2|:1-503352 Homo sapiens chromosome 11 genomic contig, GRCh37 reference primary assembly | | |
| Homo__sapiens | | >ref|NT_167190.1|:1-41593379 Homo sapiens chromosome 11 genomic contig, GRCh37 reference primary assembly | | |
| Homo__sapiens | | >ref|NT_033899.8|:1-38508932 Homo sapiens chromosome 11 genomic contig, GRCh37 reference primary assembly | | |
| Homo__sapiens | | >ref|NT_009759.16|:1-7129876 Homo sapiens chromosome 12 genomic contig, GRCh37 reference primary assembly | | |
| Homo__sapiens | | >ref|NT_009714.17|:1-27616818 Homo sapiens chromosome 12 genomic contig, GRCh37 reference primary assembly | | |
| Homo__sapiens | | >ref|NT_029419.12|:1-71516776 Homo sapiens chromosome 12 genomic contig, GRCh37 reference primary assembly | | |
| Homo__sapiens | | >ref|NT_009775.17|:1-13107153 Homo sapiens chromosome 12 genomic contig, GRCh37 reference primary assembly | | |
| Homo__sapiens | | >ref|NT_009755.19|:1-10126369 Homo sapiens chromosome 12 genomic contig, GRCh37 reference primary assembly | | |
| Homo__sapiens | | >ref|NT_024477.14|:1-1034903 Homo sapiens chromosome 12 genomic contig, GRCh37 reference primary assembly | | |
| Homo__sapiens | | >ref|NT_024524.14|:1-67740324 Homo sapiens chromosome 13 genomic contig, GRCh37 reference primary assembly | | |
| Homo__sapiens | | >ref|NT_009952.14|:1-25443670 Homo sapiens chromosome 13 genomic contig, GRCh37 reference primary assembly | | |
| Homo__sapiens | | >ref|NT_027140.6|:1-1821999 Homo sapiens chromosome 13 genomic contig, GRCh37 reference primary assembly | | |
| Homo__sapiens | | >ref|NT_077627.3|:1-213955 Homo sapiens chromosome 13 genomic contig, GRCh37 reference primary assembly | | |
| Homo__sapiens | | >ref|NT_024498.12|:1-369930 Homo sapiens chromosome 13 genomic contig, GRCh37 reference primary assembly | | |
| Homo__sapiens | | >ref|NT_026437.12|:1-88289540 Homo sapiens chromosome 14 genomic contig, GRCh37 reference primary assembly | | |
| Homo__sapiens | | >ref|NT_037852.6|:1-2212114 Homo sapiens chromosome 15 genomic contig, GRCh37 reference primary assembly | | |
| Homo__sapiens | | >ref|NT_077631.1|:1-334079 Homo sapiens chromosome 15 genomic contig, GRCh37 reference primary assembly | | |
| Homo__sapiens | | >ref|NT_078094.2|:1-868660 Homo sapiens chromosome 15 genomic contig, GRCh37 reference primary assembly | | |
| Homo__sapiens | | >ref|NT_026446.14|:1-5594590 Homo sapiens chromosome 15 genomic contig, GRCh37 reference primary assembly | | |
| Homo__sapiens | | >ref|NT_010194.17|:1-53620202 Homo sapiens chromosome 15 genomic contig, GRCh37 reference primary assembly | | |
| Homo__sapiens | | >ref|NT_077661.3|:1-2104828 Homo sapiens chromosome 15 genomic contig, GRCh37 reference primary assembly | | |
| Homo__sapiens | | >ref|NT_010274.17|:1-17486919 Homo sapiens chromosome 15 genomic contig, GRCh37 reference primary assembly | | |
| Homo__sapiens | | >ref|NT_010393.16|:1-33963150 Homo sapiens chromosome 16 genomic contig, GRCh37 reference primary assembly | | |
| Homo__sapiens | | >ref|NT_024773.11|:1-1112651 Homo sapiens chromosome 16 genomic contig, GRCh37 reference primary assembly | | |
| Homo__sapiens | | >ref|NT_010498.15|:1-42003582 Homo sapiens chromosome 16 genomic contig, GRCh37 reference primary assembly | | |
| Homo__sapiens | | >ref|NT_010542.15|:1-1855370 Homo sapiens chromosome 16 genomic contig, GRCh37 reference primary assembly | | |
| Homo__sapiens | | >ref|NT_024972.8|:1-296626 Homo sapiens chromosome 17 genomic contig, GRCh37 reference primary assembly | | |
| Homo__sapiens | | >ref|NT_010718.16|:1-21169982 Homo sapiens chromosome 17 genomic contig, GRCh37 reference primary assembly | | |
| Homo__sapiens | | >ref|NT_024862.14|:1-596398 Homo sapiens chromosome 17 genomic contig, GRCh37 reference primary assembly | | |
| Homo__sapiens | | >ref|NT_010799.15|:1-9412842 Homo sapiens chromosome 17 genomic contig, GRCh37 reference primary assembly | | |
| Homo__sapiens | | >ref|NT_010783.15|:1-44983201 Homo sapiens chromosome 17 genomic contig, GRCh37 reference primary assembly | | |
| Homo__sapiens | | >ref|NT_010663.15|:1-1436161 Homo sapiens chromosome 17 genomic contig, GRCh37 reference primary assembly | | |
| Homo__sapiens | | >ref|NT_010859.14|:1-15400898 Homo sapiens chromosome 18 genomic contig, GRCh37 reference primary assembly | | |
| Homo__sapiens | | >ref|NT_010966.14|:1-33548238 Homo sapiens chromosome 18 genomic contig, GRCh37 reference primary assembly | | |
| Homo__sapiens | | >ref|NT_025028.14|:1-25808112 Homo sapiens chromosome 18 genomic contig, GRCh37 reference primary assembly | | |
| Homo__sapiens | | >ref|NT_011255.14|:1-7286004 Homo sapiens chromosome 19 genomic contig, GRCh37 reference primary assembly | | |
| Homo__sapiens | | >ref|NT_077812.2|:1-1291194 Homo sapiens chromosome 19 genomic contig, GRCh37 reference primary assembly | | |
| Homo__sapiens | | >ref|NT_011295.11|:1-15894584 Homo sapiens chromosome 19 genomic contig, GRCh37 reference primary assembly | | |
| Homo__sapiens | | >ref|NT_011109.16|:1-31387201 Homo sapiens chromosome 19 genomic contig, GRCh37 reference primary assembly | | |
| Homo__sapiens | | >ref|NT_077402.2|:1-257719 Homo sapiens chromosome 1 genomic contig, GRCh37 reference primary assembly | | |
| Homo__sapiens | | >ref|NT_113797.1|:1-126477 Homo sapiens chromosome 1 genomic contig, GRCh37 reference primary assembly | | |
| Homo__sapiens | | >ref|NT_079485.4|:1-224781 Homo sapiens chromosome 1 genomic contig, GRCh37 reference primary assembly | | |
| Homo__sapiens | | >ref|NT_079497.3|:1-78698 Homo sapiens chromosome 1 genomic contig, GRCh37 reference primary assembly | | |
| Homo__sapiens | | >ref|NT_077933.2|:1-347932 Homo sapiens chromosome 1 genomic contig, GRCh37 reference primary assembly | | |
| Homo__sapiens | | >ref|NT_167185.1|:1-3353625 Homo sapiens chromosome 1 genomic contig, GRCh37 reference primary assembly | | |
| Homo__sapiens | | >ref|NT_113799.1|:1-185320 Homo sapiens chromosome 1 genomic contig, GRCh37 reference primary assembly | | |
| Homo__sapiens | | >ref|NT_078067.3|:1-376183 Homo sapiens chromosome 9 genomic contig, GRCh37 reference primary assembly | | |
| Homo__sapiens | | >ref|NT_086602.2|:1-259514 Homo sapiens chromosome 1 genomic contig, GRCh37 reference primary assembly | | |
| Homo__sapiens | | >ref|NT_167186.1|:1-42425989 Homo sapiens chromosome 1 genomic contig, GRCh37 reference primary assembly | | |
| Homo__sapiens | | >ref|NT_032968.8|:1-182411 Homo sapiens chromosome 1 genomic contig, GRCh37 reference primary assembly | | |
| Homo__sapiens | | >ref|NT_077912.1|:1-153649 Homo sapiens chromosome 1 genomic contig, GRCh37 reference primary assembly | | |
| Homo__sapiens | | >ref|NT_004350.19|:1-3323900 Homo sapiens chromosome 1 genomic contig, GRCh37 reference primary assembly | | |
| Homo__sapiens | | >ref|NT_021937.19|:1-9224644 Homo sapiens chromosome 1 genomic contig, GRCh37 reference primary assembly | | |
| Homo__sapiens | | >ref|NT_004610.19|:1-16558170 Homo sapiens chromosome 1 genomic contig, GRCh37 reference primary assembly | | |
| Homo__sapiens | | >ref|NT_032977.9|:1-90908613 Homo sapiens chromosome 1 genomic contig, GRCh37 reference primary assembly | | |
| Homo__sapiens | | >ref|NT_077389.3|:1-398739 Homo sapiens chromosome 1 genomic contig, GRCh37 reference primary assembly | | |
| Homo__sapiens | | >ref|NT_113793.2|:1-432327 Homo sapiens chromosome 1 genomic contig, GRCh37 reference primary assembly | | |
| Homo__sapiens | | >ref|NT_113796.2|:1-426764 Homo sapiens chromosome 1 genomic contig, GRCh37 reference primary assembly | | |
| Homo__sapiens | | >ref|NT_011387.8|:1-26259569 Homo sapiens chromosome 20 genomic contig, GRCh37 reference primary assembly | | |
| Homo__sapiens | | >ref|NT_025215.4|:1-234339 Homo sapiens chromosome 20 genomic contig, GRCh37 reference primary assembly | | |
| Homo__sapiens | | >ref|NT_011362.10|:1-31409461 Homo sapiens chromosome 20 genomic contig, GRCh37 reference primary assembly | | |
| Homo__sapiens | | >ref|NT_011333.6|:1-1702151 Homo sapiens chromosome 20 genomic contig, GRCh37 reference primary assembly | | |
| Homo__sapiens | | >ref|NT_113952.1|:1-184355 Homo sapiens chromosome 21 genomic contig, GRCh37 reference primary assembly | | |
| Homo__sapiens | | >ref|NT_113954.1|:1-129889 Homo sapiens chromosome 21 genomic contig, GRCh37 reference primary assembly | | |
| Homo__sapiens | | >ref|NT_113958.2|:1-209483 Homo sapiens chromosome 21 genomic contig, GRCh37 reference primary assembly | | |
| Homo__sapiens | | >ref|NT_113953.1|:1-131056 Homo sapiens chromosome 21 genomic contig, GRCh37 reference primary assembly | | |
| Homo__sapiens | | >ref|NT_113955.2|:1-281920 Homo sapiens chromosome 21 genomic contig, GRCh37 reference primary assembly | | |
| Homo__sapiens | | >ref|NT_029490.4|:1-490233 Homo sapiens chromosome 21 genomic contig, GRCh37 reference primary assembly | | |
| Homo__sapiens | | >ref|NT_011512.11|:1-28617430 Homo sapiens chromosome 21 genomic contig, GRCh37 reference primary assembly | | |
| Homo__sapiens | | >ref|NT_011515.12|:1-5114336 Homo sapiens chromosome 21 genomic contig, GRCh37 reference primary assembly | | |
| Homo__sapiens | | >ref|NT_028395.3|:1-647850 Homo sapiens chromosome 22 genomic contig, GRCh37 reference primary assembly | | |
| Homo__sapiens | | >ref|NT_011519.10|:1-3661581 Homo sapiens chromosome 22 genomic contig, GRCh37 reference primary assembly | | |
| Homo__sapiens | | >ref|NT_011520.12|:1-29755346 Homo sapiens chromosome 22 genomic contig, GRCh37 reference primary assembly | | |
| Homo__sapiens | | >ref|NT_011526.7|:1-829789 Homo sapiens chromosome 22 genomic contig, GRCh37 reference primary assembly | | |
| Homo__sapiens | | >ref|NT_022221.13|:1-3519312 Homo sapiens chromosome 2 genomic contig, GRCh37 reference primary assembly | | |
| Homo__sapiens | | >ref|NT_005403.17|:1-84213159 Homo sapiens chromosome 2 genomic contig, GRCh37 reference primary assembly | | |
| Homo__sapiens | | >ref|NT_005120.16|:1-5748237 Homo sapiens chromosome 2 genomic contig, GRCh37 reference primary assembly | | |
| Homo__sapiens | | >ref|NT_022173.11|:1-952154 Homo sapiens chromosome 2 genomic contig, GRCh37 reference primary assembly | | |
| Homo__sapiens | | >ref|NT_005416.13|:1-2380241 Homo sapiens chromosome 2 genomic contig, GRCh37 reference primary assembly | | |
| Homo__sapiens | | >ref|NT_022139.13|:1-1439476 Homo sapiens chromosome 2 genomic contig, GRCh37 reference primary assembly | | |
| Homo__sapiens | | >ref|NT_005334.16|:1-11160936 Homo sapiens chromosome 2 genomic contig, GRCh37 reference primary assembly | | |
| Homo__sapiens | | >ref|NT_015926.15|:1-4823389 Homo sapiens chromosome 2 genomic contig, GRCh37 reference primary assembly | | |
| Homo__sapiens | | >ref|NT_022184.15|:1-68452323 Homo sapiens chromosome 2 genomic contig, GRCh37 reference primary assembly | | |
| Homo__sapiens | | >ref|NT_032994.6|:1-714667 Homo sapiens chromosome 2 genomic contig, GRCh37 reference primary assembly | | |
| Homo__sapiens | | >ref|NT_034508.2|:1-731068 Homo sapiens chromosome 2 genomic contig, GRCh37 reference primary assembly | | |
| Homo__sapiens | | >ref|NT_022171.15|:1-14783166 Homo sapiens chromosome 2 genomic contig, GRCh37 reference primary assembly | | |
| Homo__sapiens | | >ref|NT_022135.16|:1-39439245 Homo sapiens chromosome 2 genomic contig, GRCh37 reference primary assembly | | |
| Homo__sapiens | | >ref|NT_022517.18|:1-66110270 Homo sapiens chromosome 3 genomic contig, GRCh37 reference primary assembly | | |
| Homo__sapiens | | >ref|NT_022459.15|:1-24234584 Homo sapiens chromosome 3 genomic contig, GRCh37 reference primary assembly | | |
| Homo__sapiens | | >ref|NT_005612.16|:1-100537107 Homo sapiens chromosome 3 genomic contig, GRCh37 reference primary assembly | | |
| Homo__sapiens | | >ref|NT_029928.13|:1-3915179 Homo sapiens chromosome 3 genomic contig, GRCh37 reference primary assembly | | |
| Homo__sapiens | | >ref|NT_037622.5|:1-1413146 Homo sapiens chromosome 4 genomic contig, GRCh37 reference primary assembly | | |
| Homo__sapiens | | >ref|NT_016354.19|:1-115591997 Homo sapiens chromosome 4 genomic contig, GRCh37 reference primary assembly | | |
| Homo__sapiens | | >ref|NT_006051.18|:1-7320557 Homo sapiens chromosome 4 genomic contig, GRCh37 reference primary assembly | | |
| Homo__sapiens | | >ref|NT_006316.16|:1-23002714 Homo sapiens chromosome 4 genomic contig, GRCh37 reference primary assembly | | |
| Homo__sapiens | | >ref|NT_022794.10|:1-997221 Homo sapiens chromosome 4 genomic contig, GRCh37 reference primary assembly | | |
| Homo__sapiens | | >ref|NT_016297.16|:1-7455758 Homo sapiens chromosome 4 genomic contig, GRCh37 reference primary assembly | | |
| Homo__sapiens | | >ref|NT_006238.11|:1-9041845 Homo sapiens chromosome 4 genomic contig, GRCh37 reference primary assembly | | |
| Homo__sapiens | | >ref|NT_037645.2|:1-171176 Homo sapiens chromosome 4 genomic contig, GRCh37 reference primary assembly | | |
| Homo__sapiens | | >ref|NT_022853.15|:1-7079216 Homo sapiens chromosome 4 genomic contig, GRCh37 reference primary assembly | | |
| Homo__sapiens | | >ref|NT_022778.16|:1-15638046 Homo sapiens chromosome 4 genomic contig, GRCh37 reference primary assembly | | |
| Homo__sapiens | | >ref|NT_006576.16|:1-46395641 Homo sapiens chromosome 5 genomic contig, GRCh37 reference primary assembly | | |
| Homo__sapiens | | >ref|NT_006713.15|:1-42230487 Homo sapiens chromosome 5 genomic contig, GRCh37 reference primary assembly | | |
| Homo__sapiens | | >ref|NT_034772.6|:1-47100945 Homo sapiens chromosome 5 genomic contig, GRCh37 reference primary assembly | | |
| Homo__sapiens | | >ref|NT_029289.11|:1-16301654 Homo sapiens chromosome 5 genomic contig, GRCh37 reference primary assembly | | |
| Homo__sapiens | | >ref|NT_023133.13|:1-25716533 Homo sapiens chromosome 5 genomic contig, GRCh37 reference primary assembly | | |
| Homo__sapiens | | >ref|NT_007592.15|:1-58720166 Homo sapiens chromosome 6 genomic contig, GRCh37 reference primary assembly | | |
| Homo__sapiens | | >ref|NT_007299.13|:1-33800377 Homo sapiens chromosome 6 genomic contig, GRCh37 reference primary assembly | | |
| Homo__sapiens | | >ref|NT_025741.15|:1-75224524 Homo sapiens chromosome 6 genomic contig, GRCh37 reference primary assembly | | |
| Homo__sapiens | | >ref|NT_007819.17|:1-50360631 Homo sapiens chromosome 7 genomic contig, GRCh37 reference primary assembly | | |
| Homo__sapiens | | >ref|NT_033968.6|:1-7643700 Homo sapiens chromosome 7 genomic contig, GRCh37 reference primary assembly | | |
| Homo__sapiens | | >ref|NT_023603.5|:1-256182 Homo sapiens chromosome 7 genomic contig, GRCh37 reference primary assembly | | |
| Homo__sapiens | | >ref|NT_077528.2|:1-556644 Homo sapiens chromosome 7 genomic contig, GRCh37 reference primary assembly | | |
| Homo__sapiens | | >ref|NT_007933.15|:1-77412220 Homo sapiens chromosome 7 genomic contig, GRCh37 reference primary assembly | | |
| Homo__sapiens | | >ref|NT_007914.15|:1-14866257 Homo sapiens chromosome 7 genomic contig, GRCh37 reference primary assembly | | |
| Homo__sapiens | | >ref|NT_007741.14|:1-4758029 Homo sapiens chromosome 7 genomic contig, GRCh37 reference primary assembly | | |
| Homo__sapiens | | >ref|NT_023736.17|:1-7464649 Homo sapiens chromosome 8 genomic contig, GRCh37 reference primary assembly | | |
| Homo__sapiens | | >ref|NT_077531.4|:1-4567205 Homo sapiens chromosome 8 genomic contig, GRCh37 reference primary assembly | | |
| Homo__sapiens | | >ref|NT_167187.1|:1-31697033 Homo sapiens chromosome 8 genomic contig, GRCh37 reference primary assembly | | |
| Homo__sapiens | | >ref|NT_023678.16|:1-1291612 Homo sapiens chromosome 8 genomic contig, GRCh37 reference primary assembly | | |
| Homo__sapiens | | >ref|NT_008183.19|:1-38440852 Homo sapiens chromosome 8 genomic contig, GRCh37 reference primary assembly | | |
| Homo__sapiens | | >ref|NT_008046.16|:1-58606137 Homo sapiens chromosome 8 genomic contig, GRCh37 reference primary assembly | | |
| Homo__sapiens | | >ref|NT_037704.5|:1-871434 Homo sapiens chromosome 8 genomic contig, GRCh37 reference primary assembly | | |
| Homo__sapiens | | >ref|NT_008413.18|:1-39964796 Homo sapiens chromosome 9 genomic contig, GRCh37 reference primary assembly | | |
| Homo__sapiens | | >ref|NT_086755.3|:1-499094 Homo sapiens chromosome 9 genomic contig, GRCh37 reference primary assembly | | |
| Homo__sapiens | | >ref|NT_078078.3|:1-157546 Homo sapiens chromosome 9 genomic contig, GRCh37 reference primary assembly | | |
| Homo__sapiens | | >ref|NT_078052.4|:1-450681 Homo sapiens chromosome 9 genomic contig, GRCh37 reference primary assembly | | |
| Homo__sapiens | | >ref|NT_167189.1|:1-1139474 Homo sapiens chromosome 9 genomic contig, GRCh37 reference primary assembly | | |
| Homo__sapiens | | >ref|NT_079533.1|:1-158462 Homo sapiens chromosome 9 genomic contig, GRCh37 reference primary assembly | | |
| Homo__sapiens | | >ref|NT_078066.4|:1-471702 Homo sapiens chromosome 9 genomic contig, GRCh37 reference primary assembly | | |
| Homo__sapiens | | >ref|NT_078067.3|:1-376183 Homo sapiens chromosome 9 genomic contig, GRCh37 reference primary assembly | | |
| Homo__sapiens | | >ref|NT_078068.1|:1-174765 Homo sapiens chromosome 9 genomic contig, GRCh37 reference primary assembly | | |
| Homo__sapiens | | >ref|NT_078070.3|:1-1229783 Homo sapiens chromosome 9 genomic contig, GRCh37 reference primary assembly | | |
| Homo__sapiens | | >ref|NT_113816.1|:1-187806 Homo sapiens chromosome 9 genomic contig, GRCh37 reference primary assembly | | |
| Homo__sapiens | | >ref|NT_078049.4|:1-401038 Homo sapiens chromosome 9 genomic contig, GRCh37 reference primary assembly | | |
| Homo__sapiens | | >ref|NT_113817.1|:1-178933 Homo sapiens chromosome 9 genomic contig, GRCh37 reference primary assembly | | |
| Homo__sapiens | | >ref|NT_008470.19|:1-62237592 Homo sapiens chromosome 9 genomic contig, GRCh37 reference primary assembly | | |
| Homo__sapiens | | >ref|NT_035014.4|:1-3818133 Homo sapiens chromosome 9 genomic contig, GRCh37 reference primary assembly | | |
| Homo__sapiens | | >ref|NT_019501.13|:1-2075804 Homo sapiens chromosome 9 genomic contig, GRCh37 reference primary assembly | | |
| Homo__sapiens | | >ref|NT_024000.16|:1-1936434 Homo sapiens chromosome 9 genomic contig, GRCh37 reference primary assembly | | |
| Homo__sapiens | | >ref|NT_078041.2|:1-464507 Homo sapiens chromosome 9 genomic contig, GRCh37 reference primary assembly | | |
| Homo__sapiens | | >ref|NT_113812.2|:1-375452 Homo sapiens chromosome 9 genomic contig, GRCh37 reference primary assembly | | |
| Homo__sapiens | | >ref|NT_078043.5|:1-1198162 Homo sapiens chromosome 9 genomic contig, GRCh37 reference primary assembly | | |
| Homo__sapiens | | >ref|NT_078055.4|:1-549743 Homo sapiens chromosome 9 genomic contig, GRCh37 reference primary assembly | | |
| Homo__sapiens | | >ref|NT_167188.1|:1-1936505 Homo sapiens chromosome 9 genomic contig, GRCh37 reference primary assembly | | |
| Homo__sapiens | | >ref|NT_078053.4|:1-465318 Homo sapiens chromosome 9 genomic contig, GRCh37 reference primary assembly | | |
| Homo__sapiens | | >ref|NT_113813.2|:1-595518 Homo sapiens chromosome 9 genomic contig, GRCh37 reference primary assembly | | |
| Homo__sapiens | | >ref|NT_167191.1|:1-34821 Homo sapiens chromosome X genomic contig, GRCh37 reference primary assembly | | |
| Homo__sapiens | | >ref|NT_011638.13|:1-2371741 Homo sapiens chromosome X genomic contig, GRCh37 reference primary assembly | | |
| Homo__sapiens | | >ref|NT_011630.14|:1-6136098 Homo sapiens chromosome X genomic contig, GRCh37 reference primary assembly | | |
| Homo__sapiens | | >ref|NT_011669.17|:1-14971680 Homo sapiens chromosome X genomic contig, GRCh37 reference primary assembly | | |
| Homo__sapiens | | >ref|NT_011651.17|:1-36813976 Homo sapiens chromosome X genomic contig, GRCh37 reference primary assembly | | |
| Homo__sapiens | | >ref|NT_028405.12|:1-2114622 Homo sapiens chromosome X genomic contig, GRCh37 reference primary assembly | | |
| Homo__sapiens | | >ref|NT_011786.16|:1-27775034 Homo sapiens chromosome X genomic contig, GRCh37 reference primary assembly | | |
| Homo__sapiens | | >ref|NT_011681.16|:1-5474738 Homo sapiens chromosome X genomic contig, GRCh37 reference primary assembly | | |
| Homo__sapiens | | >ref|NT_167198.1|:1-6178498 Homo sapiens chromosome X genomic contig, GRCh37 reference primary assembly | | |
| Homo__sapiens | | >ref|NT_167192.1|:1-86563 Homo sapiens chromosome X genomic contig, GRCh37 reference primary assembly | | |
| Homo__sapiens | | >ref|NT_167193.1|:1-766173 Homo sapiens chromosome X genomic contig, GRCh37 reference primary assembly | | |
| Homo__sapiens | | >ref|NT_167194.1|:1-36556 Homo sapiens chromosome X genomic contig, GRCh37 reference primary assembly | | |
| Homo__sapiens | | >ref|NT_167195.1|:1-80121 Homo sapiens chromosome X genomic contig, GRCh37 reference primary assembly | | |
| Homo__sapiens | | >ref|NT_167196.1|:1-754004 Homo sapiens chromosome X genomic contig, GRCh37 reference primary assembly | | |
| Homo__sapiens | | >ref|NT_167197.1|:1-34980018 Homo sapiens chromosome X genomic contig, GRCh37 reference primary assembly | | |
| Homo__sapiens | | >ref|NT_079573.4|:1-12094741 Homo sapiens chromosome X genomic contig, GRCh37 reference primary assembly | | |
| Homo__sapiens | | >ref|NT_086939.3|:1-681176 Homo sapiens chromosome X genomic contig, GRCh37 reference primary assembly | | |
| Homo__sapiens | | >ref|NT_167199.1|:1-34821 Homo sapiens chromosome Y genomic contig, GRCh37 reference primary assembly | | |
| Homo__sapiens | | >ref|NT_011878.9|:1-813231 Homo sapiens chromosome Y genomic contig, GRCh37 reference primary assembly | | |
| Homo__sapiens | | >ref|NT_087001.1|:1-39401 Homo sapiens chromosome Y genomic contig, GRCh37 reference primary assembly | | |
| Homo__sapiens | | >ref|NT_113819.1|:1-554624 Homo sapiens chromosome Y genomic contig, GRCh37 reference primary assembly | | |
| Homo__sapiens | | >ref|NT_011875.12|:1-10102850 Homo sapiens chromosome Y genomic contig, GRCh37 reference primary assembly | | |
| Homo__sapiens | | >ref|NT_011903.12|:1-4867933 Homo sapiens chromosome Y genomic contig, GRCh37 reference primary assembly | | |
| Homo__sapiens | | >ref|NT_025975.2|:1-98295 Homo sapiens chromosome Y genomic contig, GRCh37 reference primary assembly | | |
| Homo__sapiens | | >ref|NT_091573.1|:1-66393 Homo sapiens chromosome Y genomic contig, GRCh37 reference primary assembly | | |
| Homo__sapiens | | >ref|NT_167206.1|:1-329517 Homo sapiens chromosome Y genomic contig, GRCh37 reference primary assembly | | |
| Homo__sapiens | | >ref|NT_167200.1|:1-86563 Homo sapiens chromosome Y genomic contig, GRCh37 reference primary assembly | | |
| Homo__sapiens | | >ref|NT_167201.1|:1-766173 Homo sapiens chromosome Y genomic contig, GRCh37 reference primary assembly | | |
| Homo__sapiens | | >ref|NT_167202.1|:1-36556 Homo sapiens chromosome Y genomic contig, GRCh37 reference primary assembly | | |
| Homo__sapiens | | >ref|NT_167203.1|:1-80121 Homo sapiens chromosome Y genomic contig, GRCh37 reference primary assembly | | |
| Homo__sapiens | | >ref|NT_167204.1|:1-754004 Homo sapiens chromosome Y genomic contig, GRCh37 reference primary assembly | | |
| Homo__sapiens | | >ref|NT_167205.1|:1-581282 Homo sapiens chromosome Y genomic contig, GRCh37 reference primary assembly | | |
| Homo__sapiens | | >ref|NT_011896.9|:1-6265435 Homo sapiens chromosome Y genomic contig, GRCh37 reference primary assembly | | |
| Homo__sapiens | | >ref|NT_086998.1|:1-276367 Homo sapiens chromosome Y genomic contig, GRCh37 reference primary assembly | | |
| Macaca_mulatta | | >gi|49146236|ref|NC_005943.1| Macaca mulatta mitochondrion, complete genome | | |
| Macaca_mulatta | | >gi|109156578|ref|NC_007858.1| Macaca mulatta chromosome 1, Mmul_051212 chromosome, whole genome shotgun sequence | | |
| Macaca_mulatta | | >gi|109156579|ref|NC_007867.1| Macaca mulatta chromosome 10, Mmul_051212 chromosome, whole genome shotgun sequence | | |
| Macaca_mulatta | | >gi|109156580|ref|NC_007868.1| Macaca mulatta chromosome 11, Mmul_051212 chromosome, whole genome shotgun sequence | | |
| Macaca_mulatta | | >gi|109156645|ref|NC_007869.1| Macaca mulatta chromosome 12, Mmul_051212 chromosome, whole genome shotgun sequence | | |
| Macaca_mulatta | | >gi|109156646|ref|NC_007870.1| Macaca mulatta chromosome 13, Mmul_051212 chromosome, whole genome shotgun sequence | | |
| Macaca_mulatta | | >gi|109156648|ref|NC_007871.1| Macaca mulatta chromosome 14, Mmul_051212 chromosome, whole genome shotgun sequence | | |
| Macaca_mulatta | | >gi|109156649|ref|NC_007872.1| Macaca mulatta chromosome 15, Mmul_051212 chromosome, whole genome shotgun sequence | | |
| Macaca_mulatta | | >gi|109156650|ref|NC_007873.1| Macaca mulatta chromosome 16, Mmul_051212 chromosome, whole genome shotgun sequence | | |
| Macaca_mulatta | | >gi|109156884|ref|NC_007874.1| Macaca mulatta chromosome 17, Mmul_051212 chromosome, whole genome shotgun sequence | | |
| Macaca_mulatta | | >gi|109156885|ref|NC_007875.1| Macaca mulatta chromosome 18, Mmul_051212 chromosome, whole genome shotgun sequence | | |
| Macaca_mulatta | | >gi|109156886|ref|NC_007876.1| Macaca mulatta chromosome 19, Mmul_051212 chromosome, whole genome shotgun sequence | | |
| Macaca_mulatta | | >gi|109156887|ref|NC_007859.1| Macaca mulatta chromosome 2, Mmul_051212 chromosome, whole genome shotgun sequence | | |
| Macaca_mulatta | | >gi|109156888|ref|NC_007877.1| Macaca mulatta chromosome 20, Mmul_051212 chromosome, whole genome shotgun sequence | | |
| Macaca_mulatta | | >gi|109156890|ref|NC_007860.1| Macaca mulatta chromosome 3, Mmul_051212 chromosome, whole genome shotgun sequence | | |
| Macaca_mulatta | | >gi|109156893|ref|NC_007861.1| Macaca mulatta chromosome 4, Mmul_051212 chromosome, whole genome shotgun sequence | | |
| Macaca_mulatta | | >gi|109156895|ref|NC_007862.1| Macaca mulatta chromosome 5, Mmul_051212 chromosome, whole genome shotgun sequence | | |
| Macaca_mulatta | | >gi|109157119|ref|NC_007863.1| Macaca mulatta chromosome 6, Mmul_051212 chromosome, whole genome shotgun sequence | | |
| Macaca_mulatta | | >gi|109158192|ref|NC_007864.1| Macaca mulatta chromosome 7, Mmul_051212 chromosome, whole genome shotgun sequence | | |
| Macaca_mulatta | | >gi|109158193|ref|NC_007865.1| Macaca mulatta chromosome 8, Mmul_051212 chromosome, whole genome shotgun sequence | | |
| Macaca_mulatta | | >gi|109158194|ref|NC_007866.1| Macaca mulatta chromosome 9, Mmul_051212 chromosome, whole genome shotgun sequence | | |
| Macaca_mulatta | | >gi|109158195|ref|NC_007878.1| Macaca mulatta chromosome X, Mmul_051212 chromosome, whole genome shotgun sequence | | |
| Macaca_mulatta | | >gi|90704326|ref|NW_001219154.1| Macaca mulatta chromosome X unlocalized genomic scaffold, Mmul_051212, whole genome shotgun sequence | | |
| Macaca_mulatta | | >gi|90780474|ref|NW_001211473.1| Macaca mulatta unplaced genomic scaffold, Mmul_051212, whole genome shotgun sequence | | |
| | | Medicago_truncatula | >gi|357521745|ref|NC_016407.1| Medicago truncatula chromosome 1 |  | | --- | --- | --- | | Medicago_truncatula | >gi|357521756|ref|NC_016408.1| Medicago truncatula chromosome 2 |  | | Medicago_truncatula | >gi|357521765|ref|NC_016409.1| Medicago truncatula chromosome 3 |  | | Medicago_truncatula | >gi|357521768|ref|NC_016410.1| Medicago truncatula chromosome 4 |  | | Medicago_truncatula | >gi|357521778|ref|NC_016411.1| Medicago truncatula chromosome 5 |  | | Medicago_truncatula | >gi|357521773|ref|NC_016412.1| Medicago truncatula chromosome 6 |  | | Medicago_truncatula | >gi|357521775|ref|NC_016413.1| Medicago truncatula chromosome 7 |  | | Medicago_truncatula_Chrs | >gi|357521776|ref|NC_016414.1| Medicago truncatula chromosome 8 | | | | --- | --- | --- | --- | --- | --- | --- | --- | --- | --- | --- | --- | --- | --- | --- | --- | --- | --- | --- | --- | --- | --- | --- | --- | --- | | | | | Medicago_truncatula | >gi|357521745|ref|NC_016407.1| Medicago truncatula chromosome 1 |  | | --- | --- | --- | | Medicago_truncatula | >gi|357521756|ref|NC_016408.1| Medicago truncatula chromosome 2 |  | | Medicago_truncatula | >gi|357521765|ref|NC_016409.1| Medicago truncatula chromosome 3 |  | | Medicago_truncatula | >gi|357521768|ref|NC_016410.1| Medicago truncatula chromosome 4 |  | | Medicago_truncatula | >gi|357521778|ref|NC_016411.1| Medicago truncatula chromosome 5 |  | | Medicago_truncatula | >gi|357521773|ref|NC_016412.1| Medicago truncatula chromosome 6 |  | | Medicago_truncatula | >gi|357521775|ref|NC_016413.1| Medicago truncatula chromosome 7 |  | | Medicago_truncatula_Chrs | >gi|357521776|ref|NC_016414.1| Medicago truncatula chromosome 8 | | | | --- | --- | --- | --- | --- | --- | --- | --- | --- | --- | --- | --- | --- | --- | --- | --- | --- | --- | --- | --- | --- | --- | --- | --- | --- | | | |
| Monodelphis_domestica | | >gi|126361933|ref|NC_008801.1|NC_008801 Monodelphis domestica chromosome 1, reference assembly (based on MonDom5), whole genome shotgun sequence | | |
| Monodelphis_domestica | | >gi|126362075|ref|NC_008802.1|NC_008802 Monodelphis domestica chromosome 2, reference assembly (based on MonDom5), whole genome shotgun sequence | | |
| Monodelphis_domestica | | >gi|126362809|ref|NC_008803.1|NC_008803 Monodelphis domestica chromosome 3, reference assembly (based on MonDom5), whole genome shotgun sequence | | |
| Monodelphis_domestica | | >gi|126362810|ref|NC_008804.1|NC_008804 Monodelphis domestica chromosome 4, reference assembly (based on MonDom5), whole genome shotgun sequence | | |
| Monodelphis_domestica | | >gi|126362941|ref|NC_008805.1|NC_008805 Monodelphis domestica chromosome 5, reference assembly (based on MonDom5), whole genome shotgun sequence | | |
| Monodelphis_domestica | | >gi|126362942|ref|NC_008806.1|NC_008806 Monodelphis domestica chromosome 6, reference assembly (based on MonDom5), whole genome shotgun sequence | | |
| Monodelphis_domestica | | >gi|126362943|ref|NC_008807.1|NC_008807 Monodelphis domestica chromosome 7, reference assembly (based on MonDom5), whole genome shotgun sequence | | |
| Monodelphis_domestica | | >gi|126362944|ref|NC_008808.1|NC_008808 Monodelphis domestica chromosome 8, reference assembly (based on MonDom5), whole genome shotgun sequence | | |
| Monodelphis_domestica | | >gi|126362945|ref|NC_008809.1|NC_008809 Monodelphis domestica chromosome X, reference assembly (based on MonDom5), whole genome shotgun sequence | | |
| Mus_musculus | | >ref|NT_039169.7|Mm1_39209_37:1-19423349 Mus musculus chromosome 1 genomic contig, strain C57BL/6J | | |
| Mus_musculus | | >ref|NT_039170.7|Mm1_39210_37:1-52628781 Mus musculus chromosome 1 genomic contig, strain C57BL/6J | | |
| Mus_musculus | | >ref|NT_039173.7|Mm1_39213_37:1-10225548 Mus musculus chromosome 1 genomic contig, strain C57BL/6J | | |
| Mus_musculus | | >ref|NT_078297.6|Mm1_78362_37:1-70622195 Mus musculus chromosome 1 genomic contig, strain C57BL/6J | | |
| Mus_musculus | | >ref|NT_039185.7|Mm1_39225_37:1-26822937 Mus musculus chromosome 1 genomic contig, strain C57BL/6J | | |
| Mus_musculus | | >ref|NT_039189.7|Mm1_39229_37:1-8497847 Mus musculus chromosome 1 genomic contig, strain C57BL/6J | | |
| Mus_musculus | | >ref|NT_039190.7|Mm1_39230_37:1-3319775 Mus musculus chromosome 1 genomic contig, strain C57BL/6J | | |
| Mus_musculus | | >ref|NT_039202.7|Mm2_39242_37:1-19347252 Mus musculus chromosome 2 genomic contig, strain C57BL/6J | | |
| Mus_musculus | | >ref|NT_039206.7|Mm2_39246_37:1-36511446 Mus musculus chromosome 2 genomic contig, strain C57BL/6J | | |
| Mus_musculus | | >ref|NT_039207.7|Mm2_39247_37:1-116366104 Mus musculus chromosome 2 genomic contig, strain C57BL/6J | | |
| Mus_musculus | | >ref|NT_166284.1|Mm2_163458_37:1-617724 Mus musculus chromosome 2 genomic contig, strain C57BL/6J | | |
| Mus_musculus | | >ref|NT_078355.6|Mm2_78420_37:1-1356266 Mus musculus chromosome 2 genomic contig, strain C57BL/6J | | |
| Mus_musculus | | >ref|NT_039212.6|Mm2_39252_37:1-4299295 Mus musculus chromosome 2 genomic contig, strain C57BL/6J | | |
| Mus_musculus | | >ref|NT_078380.6|Mm3_78445_37:1-12610690 Mus musculus chromosome 3 genomic contig, strain C57BL/6J | | |
| Mus_musculus | | >ref|NT_162143.3|Mm3_159849_37:1-24788849 Mus musculus chromosome 3 genomic contig, strain C57BL/6J | | |
| Mus_musculus | | >ref|NT_039229.7|Mm3_39269_37:1-10113108 Mus musculus chromosome 3 genomic contig, strain C57BL/6J | | |
| Mus_musculus | | >ref|NT_039240.7|Mm3_39280_37:1-80458789 Mus musculus chromosome 3 genomic contig, strain C57BL/6J | | |
| Mus_musculus | | >ref|NT_166285.1|Mm3_163459_37:1-28473347 Mus musculus chromosome 3 genomic contig, strain C57BL/6J | | |
| Mus_musculus | | >gi|74229896|gb|CM000224.2| Mus musculus chromosome 16, whole genome shotgun sequence | | |
| Mus_musculus | | >gi|74229895|gb|CM000225.2| Mus musculus chromosome 17, whole genome shotgun sequence | | |
| Mus_musculus | | >gi|74229894|gb|CM000226.2| Mus musculus chromosome 18, whole genome shotgun sequence | | |
| Mus_musculus | | >gi|71913465|gb|CM000227.1| Mus musculus chromosome 19, whole genome shotgun sequence | | |
| Mus_musculus | | >gi|74229906|gb|CM000214.2| Mus musculus chromosome 6, whole genome shotgun sequence | | |
| Mus_musculus | | >gi|74229905|gb|CM000215.2| Mus musculus chromosome 7, whole genome shotgun sequence | | |
| Mus_musculus | | >gi|74229893|gb|CM000228.2| Mus musculus chromosome X, whole genome shotgun sequence | | |
| Mus_musculus | | >gi|71913463|gb|CM000229.1| Mus musculus chromosome Y, whole genome shotgun sequence | | |
| Neurospora crassa | | gi|164429762|ref|NW_001849831.1| , etc. 210 contigs, whole genome shotgun sequence | | |
| Ornithorhynchus_anatinus | | >gi|149712631|ref|NC_009094.1|NC_009094 Ornithorhynchus anatinus chromosome 1, reference assembly (based on Ornithorhynchus_anatinus-5.0.1), whole genome shotgun sequence | | |
| Ornithorhynchus_anatinus | | >gi|149714148|ref|NC_009103.1|NC_009103 Ornithorhynchus anatinus chromosome 10, reference assembly (based on Ornithorhynchus_anatinus-5.0.1), whole genome shotgun sequence | | |
| Ornithorhynchus_anatinus | | >gi|149715145|ref|NC_009104.1|NC_009104 Ornithorhynchus anatinus chromosome 11, reference assembly (based on Ornithorhynchus_anatinus-5.0.1), whole genome shotgun sequence | | |
| Ornithorhynchus_anatinus | | >gi|149716377|ref|NC_009105.1|NC_009105 Ornithorhynchus anatinus chromosome 12, reference assembly (based on Ornithorhynchus_anatinus-5.0.1), whole genome shotgun sequence | | |
| Ornithorhynchus_anatinus | | >gi|149716935|ref|NC_009107.1|NC_009107 Ornithorhynchus anatinus chromosome 14, reference assembly (based on Ornithorhynchus_anatinus-5.0.1), whole genome shotgun sequence | | |
| Ornithorhynchus_anatinus | | >gi|149717714|ref|NC_009108.1|NC_009108 Ornithorhynchus anatinus chromosome 15, reference assembly (based on Ornithorhynchus_anatinus-5.0.1), whole genome shotgun sequence | | |
| Ornithorhynchus_anatinus | | >gi|149717939|ref|NC_009110.1|NC_009110 Ornithorhynchus anatinus chromosome 17, reference assembly (based on Ornithorhynchus_anatinus-5.0.1), whole genome shotgun sequence | | |
| Ornithorhynchus_anatinus | | >gi|149719225|ref|NC_009111.1|NC_009111 Ornithorhynchus anatinus chromosome 18, reference assembly (based on Ornithorhynchus_anatinus-5.0.1), whole genome shotgun sequence | | |
| Ornithorhynchus_anatinus | | >gi|149721824|ref|NC_009095.1|NC_009095 Ornithorhynchus anatinus chromosome 2, reference assembly (based on Ornithorhynchus_anatinus-5.0.1), whole genome shotgun sequence | | |
| Ornithorhynchus_anatinus | | >gi|149722085|ref|NC_009112.1|NC_009112 Ornithorhynchus anatinus chromosome 20, reference assembly (based on Ornithorhynchus_anatinus-5.0.1), whole genome shotgun sequence | | |
| Ornithorhynchus_anatinus | | >gi|149725021|ref|NC_009096.1|NC_009096 Ornithorhynchus anatinus chromosome 3, reference assembly (based on Ornithorhynchus_anatinus-5.0.1), whole genome shotgun sequence | | |
| Ornithorhynchus_anatinus | | >gi|149727112|ref|NC_009097.1|NC_009097 Ornithorhynchus anatinus chromosome 4, reference assembly (based on Ornithorhynchus_anatinus-5.0.1), whole genome shotgun sequence | | |
| Ornithorhynchus_anatinus | | >gi|149728214|ref|NC_009098.1|NC_009098 Ornithorhynchus anatinus chromosome 5, reference assembly (based on Ornithorhynchus_anatinus-5.0.1), whole genome shotgun sequence | | |
| Ornithorhynchus_anatinus | | >gi|149729612|ref|NC_009099.1|NC_009099 Ornithorhynchus anatinus chromosome 6, reference assembly (based on Ornithorhynchus_anatinus-5.0.1), whole genome shotgun sequence | | |
| Ornithorhynchus_anatinus | | >gi|149731469|ref|NC_009100.1|NC_009100 Ornithorhynchus anatinus chromosome 7, reference assembly (based on Ornithorhynchus_anatinus-5.0.1), whole genome shotgun sequence | | |
| Ornithorhynchus_anatinus | | >gi|149737093|ref|NC_009114.1|NC_009114 Ornithorhynchus anatinus chromosome X1, reference assembly (based on Ornithorhynchus_anatinus-5.0.1), whole genome shotgun sequence | | |
| Ornithorhynchus_anatinus | | >gi|149737330|ref|NC_009115.1|NC_009115 Ornithorhynchus anatinus chromosome X2, reference assembly (based on Ornithorhynchus_anatinus-5.0.1), whole genome shotgun sequence | | |
| Ornithorhynchus_anatinus | | >gi|149737646|ref|NC_009116.1|NC_009116 Ornithorhynchus anatinus chromosome X3, reference assembly (based on Ornithorhynchus_anatinus-5.0.1), whole genome shotgun sequence | | |
| Ornithorhynchus_anatinus | | >gi|149742078|ref|NC_009118.1|NC_009118 Ornithorhynchus anatinus chromosome X5, reference assembly (based on Ornithorhynchus_anatinus-5.0.1), whole genome shotgun sequence | | |
| Oryctolagus_cuniculus | | >gi|5835526|ref|NC_001913.1| Oryctolagus cuniculus mitochondrion, complete genome | | |
| Oryctolagus_cuniculus | | >gi|283562148|ref|NC_013669.1| Oryctolagus cuniculus breed Thorbecke inbred chromosome 1, OryCun2.0, whole genome shotgun sequence | | |
| Oryctolagus_cuniculus | | >gi|283562139|ref|NC_013678.1| Oryctolagus cuniculus breed Thorbecke inbred chromosome 10, OryCun2.0, whole genome shotgun sequence | | |
| Oryctolagus_cuniculus | | >gi|283562138|ref|NC_013679.1| Oryctolagus cuniculus breed Thorbecke inbred chromosome 11, OryCun2.0, whole genome shotgun sequence | | |
| Oryctolagus_cuniculus | | >gi|283562137|ref|NC_013680.1| Oryctolagus cuniculus breed Thorbecke inbred chromosome 12, OryCun2.0, whole genome shotgun sequence | | |
| Oryctolagus_cuniculus | | >gi|283562136|ref|NC_013681.1| Oryctolagus cuniculus breed Thorbecke inbred chromosome 13, OryCun2.0, whole genome shotgun sequence | | |
| Oryctolagus_cuniculus | | >gi|283562135|ref|NC_013682.1| Oryctolagus cuniculus breed Thorbecke inbred chromosome 14, OryCun2.0, whole genome shotgun sequence | | |
| Oryctolagus_cuniculus | | >gi|283562134|ref|NC_013683.1| Oryctolagus cuniculus breed Thorbecke inbred chromosome 15, OryCun2.0, whole genome shotgun sequence | | |
| Oryctolagus_cuniculus | | >gi|283562133|ref|NC_013684.1| Oryctolagus cuniculus breed Thorbecke inbred chromosome 16, OryCun2.0, whole genome shotgun sequence | | |
| Oryctolagus_cuniculus | | >gi|283562132|ref|NC_013685.1| Oryctolagus cuniculus breed Thorbecke inbred chromosome 17, OryCun2.0, whole genome shotgun sequence | | |
| Oryctolagus_cuniculus | | >gi|283562131|ref|NC_013686.1| Oryctolagus cuniculus breed Thorbecke inbred chromosome 18, OryCun2.0, whole genome shotgun sequence | | |
| Oryctolagus_cuniculus | | >gi|283562130|ref|NC_013687.1| Oryctolagus cuniculus breed Thorbecke inbred chromosome 19, OryCun2.0, whole genome shotgun sequence | | |
| Oryctolagus_cuniculus | | >gi|283562147|ref|NC_013670.1| Oryctolagus cuniculus breed Thorbecke inbred chromosome 2, OryCun2.0, whole genome shotgun sequence | | |
| Oryctolagus_cuniculus | | >gi|283562129|ref|NC_013688.1| Oryctolagus cuniculus breed Thorbecke inbred chromosome 20, OryCun2.0, whole genome shotgun sequence | | |
| Oryctolagus_cuniculus | | >gi|283562128|ref|NC_013689.1| Oryctolagus cuniculus breed Thorbecke inbred chromosome 21, OryCun2.0, whole genome shotgun sequence | | |
| Oryctolagus_cuniculus | | >gi|283562146|ref|NC_013671.1| Oryctolagus cuniculus breed Thorbecke inbred chromosome 3, OryCun2.0, whole genome shotgun sequence | | |
| Oryctolagus_cuniculus | | >gi|283562145|ref|NC_013672.1| Oryctolagus cuniculus breed Thorbecke inbred chromosome 4, OryCun2.0, whole genome shotgun sequence | | |
| Oryctolagus_cuniculus | | >gi|283562144|ref|NC_013673.1| Oryctolagus cuniculus breed Thorbecke inbred chromosome 5, OryCun2.0, whole genome shotgun sequence | | |
| Oryctolagus_cuniculus | | >gi|283562143|ref|NC_013674.1| Oryctolagus cuniculus breed Thorbecke inbred chromosome 6, OryCun2.0, whole genome shotgun sequence | | |
| Oryctolagus_cuniculus | | >gi|283562142|ref|NC_013675.1| Oryctolagus cuniculus breed Thorbecke inbred chromosome 7, OryCun2.0, whole genome shotgun sequence | | |
| Oryctolagus_cuniculus | | >gi|283562141|ref|NC_013676.1| Oryctolagus cuniculus breed Thorbecke inbred chromosome 8, OryCun2.0, whole genome shotgun sequence | | |
| Oryctolagus_cuniculus | | >gi|283562140|ref|NC_013677.1| Oryctolagus cuniculus breed Thorbecke inbred chromosome 9, OryCun2.0, whole genome shotgun sequence | | |
| Oryctolagus_cuniculus | | >gi|283562127|ref|NC_013690.1| Oryctolagus cuniculus breed Thorbecke inbred chromosome X, OryCun2.0, whole genome shotgun sequence | | |
| Oryctolagus_cuniculus | | >gi|283554652|ref|NW_003162542.1| Oryctolagus cuniculus breed Thorbecke inbred unplaced genomic scaffold, OryCun2.0 chrUn3219, whole genome shotgun sequence | | |
| Oryza_sativa_japonica | | >ref|NC_008394.1|:1-43261740 Oryza sativa (japonica cultivar-group) genomic DNA, chromosome 1 | | |
| Oryza_sativa_japonica | | >ref|NC_008403.1|:1-22685906 Oryza sativa (japonica cultivar-group) genomic DNA, chromosome 10 | | |
| Oryza_sativa_japonica | | >ref|NC_008404.1|:1-28386948 Oryza sativa (japonica cultivar-group) genomic DNA, chromosome 11 | | |
| Oryza_sativa_japonica | | >ref|NC_008405.1|:1-27566993 Oryza sativa (japonica cultivar-group) genomic DNA, chromosome 12 | | |
| Oryza_sativa_japonica | | >ref|NC_008395.1|:1-35954743 Oryza sativa (japonica cultivar-group) genomic DNA, chromosome 2 | | |
| Oryza_sativa_japonica | | >ref|NC_008396.1|:1-36192742 Oryza sativa (japonica cultivar-group) genomic DNA, chromosome 3 | | |
| Oryza_sativa_japonica | | >ref|NC_008397.1|:1-35498469 Oryza sativa (japonica cultivar-group) genomic DNA, chromosome 4 | | |
| Oryza_sativa_japonica | | >ref|NC_008398.1|:1-29737217 Oryza sativa (japonica cultivar-group) genomic DNA, chromosome 5 | | |
| Oryza_sativa_japonica | | >ref|NC_008399.1|:1-30731886 Oryza sativa (japonica cultivar-group) genomic DNA, chromosome 6 | | |
| Oryza_sativa_japonica | | >ref|NC_008400.1|:1-29644043 Oryza sativa (japonica cultivar-group) genomic DNA, chromosome 7 | | |
| Oryza_sativa_japonica | | >ref|NC_008401.1|:1-28434780 Oryza sativa (japonica cultivar-group) genomic DNA, chromosome 8 | | |
| Oryza_sativa_japonica | | >ref|NC_008402.1|:1-22696651 Oryza sativa (japonica cultivar-group) genomic DNA, chromosome 9 | | |
| Pan_troglodytes | | >gi|114795050|ref|NC_006468.2|NC_006468 Pan troglodytes chromosome 1, reference assembly (based on Pan_troglodytes-2.1) | | |
| Pan_troglodytes | | >gi|114795051|ref|NC_006477.2|NC_006477 Pan troglodytes chromosome 10, reference assembly (based on Pan_troglodytes-2.1) | | |
| Pan_troglodytes | | >gi|114795052|ref|NC_006478.2|NC_006478 Pan troglodytes chromosome 11, reference assembly (based on Pan_troglodytes-2.1) | | |
| Pan_troglodytes | | >gi|114795053|ref|NC_006479.2|NC_006479 Pan troglodytes chromosome 12, reference assembly (based on Pan_troglodytes-2.1) | | |
| Pan_troglodytes | | >gi|114795054|ref|NC_006480.2|NC_006480 Pan troglodytes chromosome 13, reference assembly (based on Pan_troglodytes-2.1) | | |
| Pan_troglodytes | | >gi|114795055|ref|NC_006481.2|NC_006481 Pan troglodytes chromosome 14, reference assembly (based on Pan_troglodytes-2.1) | | |
| Pan_troglodytes | | >gi|114795056|ref|NC_006482.2|NC_006482 Pan troglodytes chromosome 15, reference assembly (based on Pan_troglodytes-2.1) | | |
| Pan_troglodytes | | >gi|114795057|ref|NC_006483.2|NC_006483 Pan troglodytes chromosome 16, reference assembly (based on Pan_troglodytes-2.1) | | |
| Pan_troglodytes | | >gi|114795065|ref|NC_006484.2|NC_006484 Pan troglodytes chromosome 17, reference assembly (based on Pan_troglodytes-2.1) | | |
| Pan_troglodytes | | >gi|114795066|ref|NC_006485.2|NC_006485 Pan troglodytes chromosome 18, reference assembly (based on Pan_troglodytes-2.1) | | |
| Pan_troglodytes | | >gi|114795187|ref|NC_006486.2|NC_006486 Pan troglodytes chromosome 19, reference assembly (based on Pan_troglodytes-2.1) | | |
| Pan_troglodytes | | >gi|114795211|ref|NC_006487.2|NC_006487 Pan troglodytes chromosome 20, reference assembly (based on Pan_troglodytes-2.1) | | |
| Pan_troglodytes | | >gi|114795212|ref|NC_006488.2|NC_006488 Pan troglodytes chromosome 21, reference assembly (based on Pan_troglodytes-2.1) | | |
| Pan_troglodytes | | >gi|114795213|ref|NC_006489.2|NC_006489 Pan troglodytes chromosome 22, reference assembly (based on Pan_troglodytes-2.1) | | |
| Pan_troglodytes | | >gi|114795440|ref|NC_006469.2|NC_006469 Pan troglodytes chromosome 2A, reference assembly (based on Pan_troglodytes-2.1) | | |
| Pan_troglodytes | | >gi|114796131|ref|NC_006470.2|NC_006470 Pan troglodytes chromosome 2B, reference assembly (based on Pan_troglodytes-2.1) | | |
| Pan_troglodytes | | >gi|114796132|ref|NC_006490.2|NC_006490 Pan troglodytes chromosome 3, reference assembly (based on Pan_troglodytes-2.1) | | |
| Pan_troglodytes | | >gi|114796133|ref|NC_006471.2|NC_006471 Pan troglodytes chromosome 4, reference assembly (based on Pan_troglodytes-2.1) | | |
| Pan_troglodytes | | >gi|114796134|ref|NC_006472.2|NC_006472 Pan troglodytes chromosome 5, reference assembly (based on Pan_troglodytes-2.1) | | |
| Pan_troglodytes | | >gi|114796135|ref|NC_006473.2|NC_006473 Pan troglodytes chromosome 6, reference assembly (based on Pan_troglodytes-2.1) | | |
| Pan_troglodytes | | >gi|114796136|ref|NC_006474.2|NC_006474 Pan troglodytes chromosome 7, reference assembly (based on Pan_troglodytes-2.1) | | |
| Pan_troglodytes | | >gi|114796137|ref|NC_006475.2|NC_006475 Pan troglodytes chromosome 8, reference assembly (based on Pan_troglodytes-2.1) | | |
| Pan_troglodytes | | >gi|114796138|ref|NC_006476.2|NC_006476 Pan troglodytes chromosome 9, reference assembly (based on Pan_troglodytes-2.1) | | |
| Pan_troglodytes | | >gi|114796139|ref|NC_006491.2|NC_006491 Pan troglodytes chromosome X, reference assembly (based on Pan_troglodytes-2.1) | | |
| Pan_troglodytes | | >gi|114796141|ref|NC_006492.2|NC_006492 Pan troglodytes chromosome Y, reference assembly (based on Pan_troglodytes-2.1) | | |
| Plasmodium_falciparum | | >gi|254922366|gb|AE014185.2| Plasmodium falciparum 3D7 chromosome 10, complete sequence | | |
| Pongo_abelii | | >gi|5835834|ref|NC_002083.1| Pongo abelii mitochondrion, complete genome | | |
| Pongo_abelii | | >gi|241864942|ref|NC_012591.1| Pongo abelii chromosome 1, P_pygmaeus_2.0.2 | | |
| Pongo_abelii | | >gi|241864932|ref|NC_012601.1| Pongo abelii chromosome 10, P_pygmaeus_2.0.2 | | |
| Pongo_abelii | | >gi|241864931|ref|NC_012602.1| Pongo abelii chromosome 11, P_pygmaeus_2.0.2 | | |
| Pongo_abelii | | >gi|241864930|ref|NC_012603.1| Pongo abelii chromosome 12, P_pygmaeus_2.0.2 | | |
| Pongo_abelii | | >gi|241864929|ref|NC_012604.1| Pongo abelii chromosome 13, P_pygmaeus_2.0.2 | | |
| Pongo_abelii | | >gi|241864928|ref|NC_012605.1| Pongo abelii chromosome 14, P_pygmaeus_2.0.2 | | |
| Pongo_abelii | | >gi|241864927|ref|NC_012606.1| Pongo abelii chromosome 15, P_pygmaeus_2.0.2 | | |
| Pongo_abelii | | >gi|241864926|ref|NC_012607.1| Pongo abelii chromosome 16, P_pygmaeus_2.0.2 | | |
| Pongo_abelii | | >gi|241864925|ref|NC_012608.1| Pongo abelii chromosome 17, P_pygmaeus_2.0.2 | | |
| Pongo_abelii | | >gi|241864924|ref|NC_012609.1| Pongo abelii chromosome 18, P_pygmaeus_2.0.2 | | |
| Pongo_abelii | | >gi|241864923|ref|NC_012610.1| Pongo abelii chromosome 19, P_pygmaeus_2.0.2 | | |
| Pongo_abelii | | >gi|241864922|ref|NC_012611.1| Pongo abelii chromosome 20, P_pygmaeus_2.0.2 | | |
| Pongo_abelii | | >gi|241864921|ref|NC_012612.1| Pongo abelii chromosome 21, P_pygmaeus_2.0.2 | | |
| Pongo_abelii | | >gi|241864895|ref|NC_012613.1| Pongo abelii chromosome 22, P_pygmaeus_2.0.2 | | |
| Pongo_abelii | | >gi|241864941|ref|NC_012592.1| Pongo abelii chromosome 2A, P_pygmaeus_2.0.2 | | |
| Pongo_abelii | | >gi|241864940|ref|NC_012593.1| Pongo abelii chromosome 2B, P_pygmaeus_2.0.2 | | |
| Pongo_abelii | | >gi|241864939|ref|NC_012594.1| Pongo abelii chromosome 3, P_pygmaeus_2.0.2 | | |
| Pongo_abelii | | >gi|241864938|ref|NC_012595.1| Pongo abelii chromosome 4, P_pygmaeus_2.0.2 | | |
| Pongo_abelii | | >gi|241864937|ref|NC_012596.1| Pongo abelii chromosome 5, P_pygmaeus_2.0.2 | | |
| Pongo_abelii | | >gi|241864936|ref|NC_012597.1| Pongo abelii chromosome 6, P_pygmaeus_2.0.2 | | |
| Pongo_abelii | | >gi|241864935|ref|NC_012598.1| Pongo abelii chromosome 7, P_pygmaeus_2.0.2 | | |
| Pongo_abelii | | >gi|241864934|ref|NC_012599.1| Pongo abelii chromosome 8, P_pygmaeus_2.0.2 | | |
| Pongo_abelii | | >gi|241864933|ref|NC_012600.1| Pongo abelii chromosome 9, P_pygmaeus_2.0.2 | | |
| Pongo_abelii | | >gi|241864894|ref|NC_012614.1| Pongo abelii chromosome X, P_pygmaeus_2.0.2 | | |
| Pongo_abelii | | >gi|240988231|ref|NW_002972004.1| Pongo abelii chromosome X unlocalized genomic scaffold, P_pygmaeus_2.0.2 | | |
| Pongo_abelii | | >gi|240968845|ref|NW_002973204.1| Pongo abelii unplaced genomic scaffold, P_pygmaeus_2.0.2 | | |
| Populus_trichocarpa | | >gi|156627641|gb|AC209224.1| Populus trichocarpa chromosome POP064-N07, complete sequence | | |
| Populus_trichocarpa | | >gi|88853762|gb|AC182672.2| Populus trichocarpa chromosome Pop1-25F8, complete sequence | | |
| Populus_trichocarpa | | >gi|116047841|gb|CM000337.1| Populus trichocarpa linkage group I chromosome, whole genome shotgun sequence | | |
| Populus_trichocarpa | | >gi|116047832|gb|CM000346.1| Populus trichocarpa linkage group X chromosome, whole genome shotgun sequence | | |
| Populus_trichocarpa | | >gi|116047831|gb|CM000347.1| Populus trichocarpa linkage group XI chromosome, whole genome shotgun sequence | | |
| Populus_trichocarpa | | >gi|116047830|gb|CM000348.1| Populus trichocarpa linkage group XII chromosome, whole genome shotgun sequence | | |
| Populus_trichocarpa | | >gi|116047829|gb|CM000349.1| Populus trichocarpa linkage group XIII chromosome, whole genome shotgun sequence | | |
| Populus_trichocarpa | | >gi|116047828|gb|CM000350.1| Populus trichocarpa linkage group XIV chromosome, whole genome shotgun sequence | | |
| Populus_trichocarpa | | >gi|116047827|gb|CM000351.1| Populus trichocarpa linkage group XV chromosome, whole genome shotgun sequence | | |
| Populus_trichocarpa | | >gi|116047826|gb|CM000352.1| Populus trichocarpa linkage group XVI chromosome, whole genome shotgun sequence | | |
| Populus_trichocarpa | | >gi|116047825|gb|CM000353.1| Populus trichocarpa linkage group XVII chromosome, whole genome shotgun sequence | | |
| Populus_trichocarpa | | >gi|116047824|gb|CM000354.1| Populus trichocarpa linkage group XVIII chromosome, whole genome shotgun sequence | | |
| Populus_trichocarpa | | >gi|116047823|gb|CM000355.1| Populus trichocarpa linkage group XIX chromosome, whole genome shotgun sequence | | |
| Populus_trichocarpa | | >gi|116047839|gb|CM000339.1| Populus trichocarpa linkage group III chromosome, whole genome shotgun sequence | | |
| Populus_trichocarpa | | >gi|116047838|gb|CM000340.1| Populus trichocarpa linkage group IV chromosome, whole genome shotgun sequence | | |
| Populus_trichocarpa | | >gi|116047837|gb|CM000341.1| Populus trichocarpa linkage group V chromosome, whole genome shotgun sequence | | |
| Populus_trichocarpa | | >gi|116047836|gb|CM000342.1| Populus trichocarpa linkage group VI chromosome, whole genome shotgun sequence | | |
| Populus_trichocarpa | | >gi|116047835|gb|CM000343.1| Populus trichocarpa linkage group VII chromosome, whole genome shotgun sequence | | |
| Populus_trichocarpa | | >gi|116047834|gb|CM000344.1| Populus trichocarpa linkage group VIII chromosome, whole genome shotgun sequence | | |
| Populus_trichocarpa | | >gi|116047833|gb|CM000345.1| Populus trichocarpa linkage group IX chromosome, whole genome shotgun sequence | | |
| Rattus__norvegicus | | >gi|109644432|ref|AC_000069.1| Rattus norvegicus chromosome 1, alternate assembly Rn_Celera, whole genome shotgun sequence | | |
| Rattus__norvegicus | | >gi|109649548|ref|AC_000078.1| Rattus norvegicus chromosome 10, alternate assembly Rn_Celera, whole genome shotgun sequence | | |
| Rattus__norvegicus | | >gi|109649549|ref|AC_000079.1| Rattus norvegicus chromosome 11, alternate assembly Rn_Celera, whole genome shotgun sequence | | |
| Rattus__norvegicus | | >gi|109649550|ref|AC_000080.1| Rattus norvegicus chromosome 12, alternate assembly Rn_Celera, whole genome shotgun sequence | | |
| Rattus__norvegicus | | >gi|109649551|ref|AC_000081.1| Rattus norvegicus chromosome 13, alternate assembly Rn_Celera, whole genome shotgun sequence | | |
| Rattus__norvegicus | | >gi|109649552|ref|AC_000082.1| Rattus norvegicus chromosome 14, alternate assembly Rn_Celera, whole genome shotgun sequence | | |
| Rattus__norvegicus | | >gi|109649553|ref|AC_000083.1| Rattus norvegicus chromosome 15, alternate assembly Rn_Celera, whole genome shotgun sequence | | |
| Rattus__norvegicus | | >gi|109649554|ref|AC_000084.1| Rattus norvegicus chromosome 16, alternate assembly Rn_Celera, whole genome shotgun sequence | | |
| Rattus__norvegicus | | >gi|109649555|ref|AC_000085.1| Rattus norvegicus chromosome 17, alternate assembly Rn_Celera, whole genome shotgun sequence | | |
| Rattus__norvegicus | | >gi|109649556|ref|AC_000086.1| Rattus norvegicus chromosome 18, alternate assembly Rn_Celera, whole genome shotgun sequence | | |
| Rattus__norvegicus | | >gi|109649557|ref|AC_000087.1| Rattus norvegicus chromosome 19, alternate assembly Rn_Celera, whole genome shotgun sequence | | |
| Rattus__norvegicus | | >gi|109649558|ref|AC_000070.1| Rattus norvegicus chromosome 2, alternate assembly Rn_Celera, whole genome shotgun sequence | | |
| Rattus__norvegicus | | >gi|109649721|ref|AC_000088.1| Rattus norvegicus chromosome 20, alternate assembly Rn_Celera, whole genome shotgun sequence | | |
| Rattus__norvegicus | | >gi|109649722|ref|AC_000071.1| Rattus norvegicus chromosome 3, alternate assembly Rn_Celera, whole genome shotgun sequence | | |
| Rattus__norvegicus | | >gi|109652486|ref|AC_000072.1| Rattus norvegicus chromosome 4, alternate assembly Rn_Celera, whole genome shotgun sequence | | |
| Rattus__norvegicus | | >gi|109657628|ref|AC_000073.1| Rattus norvegicus chromosome 5, alternate assembly Rn_Celera, whole genome shotgun sequence | | |
| Rattus__norvegicus | | >gi|109658137|ref|AC_000074.1| Rattus norvegicus chromosome 6, alternate assembly Rn_Celera, whole genome shotgun sequence | | |
| Rattus__norvegicus | | >gi|109658138|ref|AC_000075.1| Rattus norvegicus chromosome 7, alternate assembly Rn_Celera, whole genome shotgun sequence | | |
| Rattus__norvegicus | | >gi|109658147|ref|AC_000076.1| Rattus norvegicus chromosome 8, alternate assembly Rn_Celera, whole genome shotgun sequence | | |
| Rattus__norvegicus | | >gi|109658148|ref|AC_000077.1| Rattus norvegicus chromosome 9, alternate assembly Rn_Celera, whole genome shotgun sequence | | |
| Rattus__norvegicus | | >gi|109658149|ref|AC_000089.1| Rattus norvegicus chromosome X, alternate assembly Rn_Celera, whole genome shotgun sequence | | |
| Rattus__norvegicus | | >gi|109522708|ref|NW_001091847.1| Rattus norvegicus unplaced genomic scaffold, alternate assembly Rn_Celera CRA_213000034259910, whole genome shotgun sequence | | |
| Rattus__norvegicus | | >gi|110189714|ref|NC_001665.2| Rattus norvegicus strain BN/SsNHsdMCW mitochondrion, complete genome | | |
| Rattus__norvegicus | | >gi|62750345|ref|NC_005100.2| Rattus norvegicus strain BN/SsNHsdMCW chromosome 1, RGSC_v3.4 | | |
| Rattus__norvegicus | | >gi|62750810|ref|NC_005109.2| Rattus norvegicus strain BN/SsNHsdMCW chromosome 10, RGSC_v3.4 | | |
| Rattus__norvegicus | | >gi|62750811|ref|NC_005110.2| Rattus norvegicus strain BN/SsNHsdMCW chromosome 11, RGSC_v3.4 | | |
| Rattus__norvegicus | | >gi|62750812|ref|NC_005111.2| Rattus norvegicus strain BN/SsNHsdMCW chromosome 12, RGSC_v3.4 | | |
| Rattus__norvegicus | | >gi|62750813|ref|NC_005112.2| Rattus norvegicus strain BN/SsNHsdMCW chromosome 13, RGSC_v3.4 | | |
| Rattus__norvegicus | | >gi|62750814|ref|NC_005113.2| Rattus norvegicus strain BN/SsNHsdMCW chromosome 14, RGSC_v3.4 | | |
| Rattus__norvegicus | | >gi|62750815|ref|NC_005114.2| Rattus norvegicus strain BN/SsNHsdMCW chromosome 15, RGSC_v3.4 | | |
| Rattus__norvegicus | | >gi|62750816|ref|NC_005115.2| Rattus norvegicus strain BN/SsNHsdMCW chromosome 16, RGSC_v3.4 | | |
| Rattus__norvegicus | | >gi|62750817|ref|NC_005116.2| Rattus norvegicus strain BN/SsNHsdMCW chromosome 17, RGSC_v3.4 | | |
| Rattus__norvegicus | | >gi|62750818|ref|NC_005117.2| Rattus norvegicus strain BN/SsNHsdMCW chromosome 18, RGSC_v3.4 | | |
| Rattus__norvegicus | | >gi|62750819|ref|NC_005118.2| Rattus norvegicus strain BN/SsNHsdMCW chromosome 19, RGSC_v3.4 | | |
| Rattus__norvegicus | | >gi|62750359|ref|NC_005101.2| Rattus norvegicus strain BN/SsNHsdMCW chromosome 2, RGSC_v3.4 | | |
| Rattus__norvegicus | | >gi|62750820|ref|NC_005119.2| Rattus norvegicus strain BN/SsNHsdMCW chromosome 20, RGSC_v3.4 | | |
| Rattus__norvegicus | | >gi|62750360|ref|NC_005102.2| Rattus norvegicus strain BN/SsNHsdMCW chromosome 3, RGSC_v3.4 | | |
| Rattus__norvegicus | | >gi|62750804|ref|NC_005103.2| Rattus norvegicus strain BN/SsNHsdMCW chromosome 4, RGSC_v3.4 | | |
| Rattus__norvegicus | | >gi|62750805|ref|NC_005104.2| Rattus norvegicus strain BN/SsNHsdMCW chromosome 5, RGSC_v3.4 | | |
| Rattus__norvegicus | | >gi|62750806|ref|NC_005105.2| Rattus norvegicus strain BN/SsNHsdMCW chromosome 6, RGSC_v3.4 | | |
| Rattus__norvegicus | | >gi|62750807|ref|NC_005106.2| Rattus norvegicus strain BN/SsNHsdMCW chromosome 7, RGSC_v3.4 | | |
| Rattus__norvegicus | | >gi|62750808|ref|NC_005107.2| Rattus norvegicus strain BN/SsNHsdMCW chromosome 8, RGSC_v3.4 | | |
| Rattus__norvegicus | | >gi|62750809|ref|NC_005108.2| Rattus norvegicus strain BN/SsNHsdMCW chromosome 9, RGSC_v3.4 | | |
| Rattus__norvegicus | | >gi|62750821|ref|NC_005120.2| Rattus norvegicus strain BN/SsNHsdMCW chromosome X, RGSC_v3.4 | | |
| Rattus__norvegicus | | >gi|34881901|ref|NW_048060.1| Rattus norvegicus strain BN/SsNHsdMCW chromosome X unlocalized genomic scaffold, RGSC_v3.4 | | |
| Rattus__norvegicus | | >gi|34882973|ref|NW_047942.1| Rattus norvegicus strain BN/SsNHsdMCW unplaced genomic scaffold, RGSC_v3.4 | | |
| Saccharomyces_cerevisiae_uid128 | | >Saccharomyces cerevisiae S288c chromosome I, complete sequence. | | |
| Saccharomyces_cerevisiae_uid128 | | >Saccharomyces cerevisiae S288c chromosome II, complete sequence. | | |
| Saccharomyces_cerevisiae_uid128 | | >Saccharomyces cerevisiae S288c chromosome III, complete sequence. | | |
| Saccharomyces_cerevisiae_uid128 | | >Saccharomyces cerevisiae S288c chromosome IV, complete sequence. | | |
| Saccharomyces_cerevisiae_uid128 | | >Saccharomyces cerevisiae S288c chromosome V, complete sequence. | | |
| Saccharomyces_cerevisiae_uid128 | | >Saccharomyces cerevisiae S288c chromosome VI, complete sequence. | | |
| Saccharomyces_cerevisiae_uid128 | | >Saccharomyces cerevisiae S288c chromosome VII, complete sequence. | | |
| Saccharomyces_cerevisiae_uid128 | | >Saccharomyces cerevisiae S288c chromosome VIII, complete sequence. | | |
| Saccharomyces_cerevisiae_uid128 | | >Saccharomyces cerevisiae S288c chromosome IX, complete sequence. | | |
| Saccharomyces_cerevisiae_uid128 | | >Saccharomyces cerevisiae S288c chromosome X, complete sequence. | | |
| Saccharomyces_cerevisiae_uid128 | | >Saccharomyces cerevisiae S288c chromosome XI, complete sequence. | | |
| Saccharomyces_cerevisiae_uid128 | | >Saccharomyces cerevisiae S288c chromosome XII, complete sequence. | | |
| Saccharomyces_cerevisiae_uid128 | | >Saccharomyces cerevisiae S288c chromosome XIII, complete sequence. | | |
| Saccharomyces_cerevisiae_uid128 | | >Saccharomyces cerevisiae S288c chromosome XIV, complete sequence. | | |
| Saccharomyces_cerevisiae_uid128 | | >Saccharomyces cerevisiae S288c chromosome XV, complete sequence. | | |
| Saccharomyces_cerevisiae_uid128 | | >Saccharomyces cerevisiae S288c chromosome XVI, complete sequence. | | |
| Saccharomyces_cerevisiae_uid128 | | >Saccharomyces cerevisiae S288c mitochondrion, complete genome. | | |
| Schizosaccharomyces_pombe | | >Schizosaccharomyces pombe mitochondrion, complete genome. | | |
| Schizosaccharomyces_pombe | | >Schizosaccharomyces pombe 972h- chromosome III, complete sequence. | | |
| Schizosaccharomyces_pombe | | >Schizosaccharomyces pombe 972h- chromosome II, complete sequence. | | |
| Schizosaccharomyces_pombe | | >Schizosaccharomyces pombe 972h- chromosome I, complete sequence. | | |
| Solanum_lycopersicum | | >gi|290755767|gb|AC238920.11| and GI for 1352 clones (not listed). | | |
| Solanum_lycopersicum | | gi|322718807|gb|CM001064.1| Solanum lycopersicum chromosome 1, whole genome shotgun sequence | | |
| Solanum_lycopersicum | | gi|322718806|gb|CM001065.1| Solanum lycopersicum chromosome 2, whole genome shotgun sequence  gi|322718806|gb|CM001065.1| Solanum lycopersicum chromosome 2, whole genome shotgun sequence | | |
| Solanum_lycopersicum | | gi|322718806|gb|CM001065.1| Solanum lycopersicum chromosome 2, whole genome shotgun sequence | | |
| Solanum_lycopersicum | | gi|322718804|gb|CM001067.1| Solanum lycopersicum chromosome 4, whole genome shotgun sequence | | |
| Solanum_lycopersicum | | gi|322718803|gb|CM001068.1| Solanum lycopersicum chromosome 5, whole genome shotgun sequence | | |
| Solanum_lycopersicum | | gi|322718802|gb|CM001069.1| Solanum lycopersicum chromosome 6, whole genome shotgun sequence (and chr 8.) | | |
| Solanum_lycopersicum | | gi|322718801|gb|CM001070.1| Solanum lycopersicum chromosome 7, whole genome shotgun sequence  gi|322718799|gb|CM001072.1| Solanum lycopersicum chromosome 9, whole genome shotgun sequence  gi|322718798|gb|CM001073.1| Solanum lycopersicum chromosome 10, whole genome shotgun sequence; and Chr 12  gi|322718797|gb|CM001074.1| Solanum lycopersicum chromosome 11, whole genome shotgun sequence |  | |
| Solanum_tuberosum | >chr01 PGSC_DM_v4.03_pseudomolecules.fasta.zip | | |  |
| Solanum_tuberosum | >chr02 | | |  |
| Solanum_tuberosum | >chr03 | | |  |
| Solanum_tuberosum | >chr04 | | |  |
| Solanum_tuberosum | >chr05 | | |  |
| Solanum_tuberosum | >chr06 | | |  |
| Solanum_tuberosum | >chr07 | | |  |
| Solanum_tuberosum | >chr08 | | |  |
| Solanum_tuberosum | >chr09 | | |  |
| Solanum_tuberosum | >chr10 | | |  |
| Solanum_tuberosum | >chr11 | | |  |
| Solanum_tuberosum | >chr12 | | |  |
| Solanum_phureja_DM | | >PGSC0003DMO000068169 | | |
| Solanum_tuberosum_RH_bacs | | >gi|197252128|gb|AC232062.1| Solanum tuberosum strain Diploid genotype RH89-039-16 chromosome 1 clone RH084F08, *** SEQUENCING IN PROGRESS ***, 15 unordered pieces. | | |
| Sorghum_bicolor | | >gi|242042636|ref|NC_012870.1| Sorghum bicolor chromosome 1, whole genome shotgun sequence | | |
| Sorghum_bicolor | | >gi|242097191|ref|NC_012879.1| Sorghum bicolor chromosome 10, whole genome shotgun sequence | | |
| Sorghum_bicolor | | >gi|242060088|ref|NC_012872.1| Sorghum bicolor chromosome 3, whole genome shotgun sequence | | |
| Sorghum_bicolor | | >gi|242067135|ref|NC_012873.1| Sorghum bicolor chromosome 4, whole genome shotgun sequence | | |
| Sorghum_bicolor | | >gi|242072114|ref|NC_012874.1| Sorghum bicolor chromosome 5, whole genome shotgun sequence | | |
| Sorghum_bicolor | | >gi|242077817|ref|NC_012875.1| Sorghum bicolor chromosome 6, whole genome shotgun sequence | | |
| Sorghum_bicolor | | >gi|242082450|ref|NC_012876.1| Sorghum bicolor chromosome 7, whole genome shotgun sequence | | |
| Sorghum_bicolor | | >gi|242086505|ref|NC_012877.1| Sorghum bicolor chromosome 8, whole genome shotgun sequence | | |
| Sorghum_bicolor | | >gi|242091636|ref|NC_012878.1| Sorghum bicolor chromosome 9, whole genome shotgun sequence | | |
| Sus_scrofa | | >gi|5835862|ref|NC_000845.1| Sus scrofa mitochondrion, complete genome | | |
| Sus_scrofa | | >gi|298162961|ref|NC_010443.2| Sus scrofa breed mixed chromosome 1, Sscrofa9.2 | | |
| Sus_scrofa | | >gi|298162952|ref|NC_010452.1| Sus scrofa breed mixed chromosome 10, Sscrofa9.2 | | |
| Sus_scrofa | | >gi|298162951|ref|NC_010453.2| Sus scrofa breed mixed chromosome 11, Sscrofa9.2 | | |
| Sus_scrofa | | >gi|298162950|ref|NC_010454.1| Sus scrofa breed mixed chromosome 12, Sscrofa9.2 | | |
| Sus_scrofa | | >gi|298162949|ref|NC_010455.2| Sus scrofa breed mixed chromosome 13, Sscrofa9.2 | | |
| Sus_scrofa | | >gi|298162948|ref|NC_010456.2| Sus scrofa breed mixed chromosome 14, Sscrofa9.2 | | |
| Sus_scrofa | | >gi|298162947|ref|NC_010457.2| Sus scrofa breed mixed chromosome 15, Sscrofa9.2 | | |
| Sus_scrofa | | >gi|298162946|ref|NC_010458.1| Sus scrofa breed mixed chromosome 16, Sscrofa9.2 | | |
| Sus_scrofa | | >gi|298162945|ref|NC_010459.2| Sus scrofa breed mixed chromosome 17, Sscrofa9.2 | | |
| Sus_scrofa | | >gi|298162944|ref|NC_010460.1| Sus scrofa breed mixed chromosome 18, Sscrofa9.2 | | |
| Sus_scrofa | | >gi|298162960|ref|NC_010444.1| Sus scrofa breed mixed chromosome 2, Sscrofa9.2 | | |
| Sus_scrofa | | >gi|298162959|ref|NC_010445.1| Sus scrofa breed mixed chromosome 3, Sscrofa9.2 | | |
| Sus_scrofa | | >gi|298162958|ref|NC_010446.2| Sus scrofa breed mixed chromosome 4, Sscrofa9.2 | | |
| Sus_scrofa | | >gi|298162957|ref|NC_010447.2| Sus scrofa breed mixed chromosome 5, Sscrofa9.2 | | |
| Sus_scrofa | | >gi|298162956|ref|NC_010448.1| Sus scrofa breed mixed chromosome 6, Sscrofa9.2 | | |
| Sus_scrofa | | >gi|298162955|ref|NC_010449.2| Sus scrofa breed mixed chromosome 7, Sscrofa9.2 | | |
| Sus_scrofa | | >gi|298162954|ref|NC_010450.1| Sus scrofa breed mixed chromosome 8, Sscrofa9.2 | | |
| Sus_scrofa | | >gi|298162953|ref|NC_010451.1| Sus scrofa breed mixed chromosome 9, Sscrofa9.2 | | |
| Sus_scrofa | | >gi|298162943|ref|NC_010461.2| Sus scrofa breed mixed chromosome X, Sscrofa9.2 | | |
| Taeniopygia_guttata | | >gi|224381666|ref|NC_011462.1|NC_011462 Taeniopygia guttata chromosome 1, reference assembly (based on Taeniopygia_guttata-3.2.4), whole genome shotgun sequence | | |
| Taeniopygia_guttata | | >gi|224381667|ref|NC_011474.1|NC_011474 Taeniopygia guttata chromosome 10, reference assembly (based on Taeniopygia_guttata-3.2.4), whole genome shotgun sequence | | |
| Taeniopygia_guttata | | >gi|224381668|ref|NC_011475.1|NC_011475 Taeniopygia guttata chromosome 11, reference assembly (based on Taeniopygia_guttata-3.2.4), whole genome shotgun sequence | | |
| Taeniopygia_guttata | | >gi|224381669|ref|NC_011476.1|NC_011476 Taeniopygia guttata chromosome 12, reference assembly (based on Taeniopygia_guttata-3.2.4), whole genome shotgun sequence | | |
| Taeniopygia_guttata | | >gi|224381670|ref|NC_011477.1|NC_011477 Taeniopygia guttata chromosome 13, reference assembly (based on Taeniopygia_guttata-3.2.4), whole genome shotgun sequence | | |
| Taeniopygia_guttata | | >gi|224381671|ref|NC_011478.1|NC_011478 Taeniopygia guttata chromosome 14, reference assembly (based on Taeniopygia_guttata-3.2.4), whole genome shotgun sequence | | |
| Taeniopygia_guttata | | >gi|224381672|ref|NC_011479.1|NC_011479 Taeniopygia guttata chromosome 15, reference assembly (based on Taeniopygia_guttata-3.2.4), whole genome shotgun sequence | | |
| Taeniopygia_guttata | | >gi|224381673|ref|NC_011480.1|NC_011480 Taeniopygia guttata chromosome 16, reference assembly (based on Taeniopygia_guttata-3.2.4), whole genome shotgun sequence | | |
| Taeniopygia_guttata | | >gi|224381674|ref|NC_011481.1|NC_011481 Taeniopygia guttata chromosome 17, reference assembly (based on Taeniopygia_guttata-3.2.4), whole genome shotgun sequence | | |
| Taeniopygia_guttata | | >gi|224381675|ref|NC_011482.1|NC_011482 Taeniopygia guttata chromosome 18, reference assembly (based on Taeniopygia_guttata-3.2.4), whole genome shotgun sequence | | |
| Taeniopygia_guttata | | >gi|224381676|ref|NC_011483.1|NC_011483 Taeniopygia guttata chromosome 19, reference assembly (based on Taeniopygia_guttata-3.2.4), whole genome shotgun sequence | | |
| Taeniopygia_guttata | | >gi|224381677|ref|NC_011463.1|NC_011463 Taeniopygia guttata chromosome 1A, reference assembly (based on Taeniopygia_guttata-3.2.4), whole genome shotgun sequence | | |
| Taeniopygia_guttata | | >gi|224381678|ref|NC_011464.1|NC_011464 Taeniopygia guttata chromosome 1B, reference assembly (based on Taeniopygia_guttata-3.2.4), whole genome shotgun sequence | | |
| Taeniopygia_guttata | | >gi|224381679|ref|NC_011465.1|NC_011465 Taeniopygia guttata chromosome 2, reference assembly (based on Taeniopygia_guttata-3.2.4), whole genome shotgun sequence | | |
| Taeniopygia_guttata | | >gi|224381680|ref|NC_011484.1|NC_011484 Taeniopygia guttata chromosome 20, reference assembly (based on Taeniopygia_guttata-3.2.4), whole genome shotgun sequence | | |
| Taeniopygia_guttata | | >gi|224381681|ref|NC_011485.1|NC_011485 Taeniopygia guttata chromosome 21, reference assembly (based on Taeniopygia_guttata-3.2.4), whole genome shotgun sequence | | |
| Taeniopygia_guttata | | >gi|224381682|ref|NC_011486.1|NC_011486 Taeniopygia guttata chromosome 22, reference assembly (based on Taeniopygia_guttata-3.2.4), whole genome shotgun sequence | | |
| Taeniopygia_guttata | | >gi|224381683|ref|NC_011487.1|NC_011487 Taeniopygia guttata chromosome 23, reference assembly (based on Taeniopygia_guttata-3.2.4), whole genome shotgun sequence | | |
| Taeniopygia_guttata | | >gi|224381684|ref|NC_011488.1|NC_011488 Taeniopygia guttata chromosome 24, reference assembly (based on Taeniopygia_guttata-3.2.4), whole genome shotgun sequence | | |
| Taeniopygia_guttata | | >gi|224381685|ref|NC_011489.1|NC_011489 Taeniopygia guttata chromosome 25, reference assembly (based on Taeniopygia_guttata-3.2.4), whole genome shotgun sequence | | |
| Taeniopygia_guttata | | >gi|224381686|ref|NC_011490.1|NC_011490 Taeniopygia guttata chromosome 26, reference assembly (based on Taeniopygia_guttata-3.2.4), whole genome shotgun sequence | | |
| Taeniopygia_guttata | | >gi|224381687|ref|NC_011491.1|NC_011491 Taeniopygia guttata chromosome 27, reference assembly (based on Taeniopygia_guttata-3.2.4), whole genome shotgun sequence | | |
| Taeniopygia_guttata | | >gi|224381688|ref|NC_011492.1|NC_011492 Taeniopygia guttata chromosome 28, reference assembly (based on Taeniopygia_guttata-3.2.4), whole genome shotgun sequence | | |
| Taeniopygia_guttata | | >gi|224381689|ref|NC_011466.1|NC_011466 Taeniopygia guttata chromosome 3, reference assembly (based on Taeniopygia_guttata-3.2.4), whole genome shotgun sequence | | |
| Taeniopygia_guttata | | >gi|224381690|ref|NC_011467.1|NC_011467 Taeniopygia guttata chromosome 4, reference assembly (based on Taeniopygia_guttata-3.2.4), whole genome shotgun sequence | | |
| Taeniopygia_guttata | | >gi|224381691|ref|NC_011468.1|NC_011468 Taeniopygia guttata chromosome 4A, reference assembly (based on Taeniopygia_guttata-3.2.4), whole genome shotgun sequence | | |
| Taeniopygia_guttata | | >gi|224381692|ref|NC_011469.1|NC_011469 Taeniopygia guttata chromosome 5, reference assembly (based on Taeniopygia_guttata-3.2.4), whole genome shotgun sequence | | |
| Taeniopygia_guttata | | >gi|224381693|ref|NC_011470.1|NC_011470 Taeniopygia guttata chromosome 6, reference assembly (based on Taeniopygia_guttata-3.2.4), whole genome shotgun sequence | | |
| Taeniopygia_guttata | | >gi|224381694|ref|NC_011471.1|NC_011471 Taeniopygia guttata chromosome 7, reference assembly (based on Taeniopygia_guttata-3.2.4), whole genome shotgun sequence | | |
| Taeniopygia_guttata | | >gi|224381695|ref|NC_011472.1|NC_011472 Taeniopygia guttata chromosome 8, reference assembly (based on Taeniopygia_guttata-3.2.4), whole genome shotgun sequence | | |
| Taeniopygia_guttata | | >gi|224381696|ref|NC_011473.1|NC_011473 Taeniopygia guttata chromosome 9, reference assembly (based on Taeniopygia_guttata-3.2.4), whole genome shotgun sequence | | |
| Taeniopygia_guttata | | >gi|224381697|ref|NC_011494.1|NC_011494 Taeniopygia guttata linkage group 2, reference assembly (based on Taeniopygia_guttata-3.2.4), whole genome shotgun sequence | | |
| Taeniopygia_guttata | | >gi|224381698|ref|NC_011495.1|NC_011495 Taeniopygia guttata linkage group 5, reference assembly (based on Taeniopygia_guttata-3.2.4), whole genome shotgun sequence | | |
| Taeniopygia_guttata | | >gi|224381699|ref|NC_011496.1|NC_011496 Taeniopygia guttata linkage group E22, reference assembly (based on Taeniopygia_guttata-3.2.4), whole genome shotgun sequence | | |
| Taeniopygia_guttata | | >gi|224381700|ref|NC_011493.1|NC_011493 Taeniopygia guttata chromosome Z, reference assembly (based on Taeniopygia_guttata-3.2.4), whole genome shotgun sequence | | |
| Takifugu_rubripes | | >gi|22430444|emb|CAAB01012381.1| Fugu rubripes whole genome shotgun assembly CONTIG_63105, whole genome shotgun sequence | | |
| Tribolium_castaneum | | >gi|189313711|ref|NC_007425.2|NC_007425 Tribolium castaneum linkage group 10, reference assembly (based on Tcas_3.0), whole genome shotgun sequence | | |
| Tribolium_castaneum | | >gi|189313713|ref|NC_007417.2|NC_007417 Tribolium castaneum linkage group 2, reference assembly (based on Tcas_3.0), whole genome shotgun sequence | | |
| Tribolium_castaneum | | >gi|189313714|ref|NC_007418.2|NC_007418 Tribolium castaneum linkage group 3, reference assembly (based on Tcas_3.0), whole genome shotgun sequence | | |
| Tribolium_castaneum | | >gi|189313718|ref|NC_007423.2|NC_007423 Tribolium castaneum linkage group 8, reference assembly (based on Tcas_3.0), whole genome shotgun sequence | | |
| Tribolium_castaneum | | >gi|189313712|ref|NC_007416.2|NC_007416 Tribolium castaneum linkage group 1=X, ref shotgun | | |
| Tribolium_castaneum | | >gi|189313719|ref|NC_007424.2|NC_007424 Tribolium castaneum linkage group 9, ref shotgun | | |
| Tribolium_castaneum | | >gi|91192192|ref|NC_007419.1|NC_007419 Tribolium castaneum linkage group 4, ref assembly shotgun | | |
| Tribolium_castaneum | | >gi|189313716|ref|NC_007421.2|NC_007421 Tribolium castaneum linkage group 6, ref | | |
| Tribolium_castaneum | | >gi|189313717|ref|NC_007422.2|NC_007422 Tribolium castaneum linkage group 7, ref | | |
| Trypanosoma_brucei | | >gi|261325979|emb|FN554964.1| Trypanosoma brucei gambiense DAL972 chromosome 1, complete sequence | | |
| Trypanosoma_cruzi | | >gi|322830686|gb|ADWP01000001.1| Trypanosoma cruzi strain Sylvio X10/1 sylviocontig_195, whole genome shotgun sequence | | |
| Xenopus_Silurana_tropicalis | | >gi|58618664|ref|NC_006839.1| Xenopus (Silurana) tropicalis mitochondrion, complete genome | | |
| Xenopus_Silurana_tropicalis | | str_ref_v4.2_chrUn.fa | | |
| Zea_mays | | >gi|284930231|gb|CM000777.2| Zea mays chromosome 1 | | |
| Zea_mays | | >gi|284930230|gb|CM000778.2| Zea mays chromosome 2 | | |
| Zea_mays | | >gi|284930229|gb|CM000779.1| Zea mays chromosome 3 | | |
| Zea_mays | | >gi|284930228|gb|CM000780.1| Zea mays chromosome 4 | | |
| Zea_mays | | >gi|284930227|gb|CM000781.1| Zea mays chromosome 5 | | |
| Zea_mays | | >gi|284930226|gb|CM000782.1| Zea mays chromosome 6 | | |
| Zea_mays | | >gi|284930225|gb|CM000783.1| Zea mays chromosome 7 | | |
| Zea_mays | | >gi|284930224|gb|CM000784.1| Zea mays chromosome 8 | | |
| Zea_mays | | >gi|284930223|gb|CM000785.1| Zea mays chromosome 9 | | |
| Zea_mays | | >gi|284930222|gb|CM000786.1| Zea mays chromosome 10 | | |
|  | |  | | |
|  | | | | |
