# Supplementary material for: RNA Polyadenylation Sites on the Genomes of Microorganisms, Animals, and Plants
Source: PLoS One. 2013 Nov 18;8(11):e79511. doi: 10.1371/journal.pone.0079511 (PMC3832601; doi:10.1371/journal.pone.0079511)
Supplement: Table S2 — Proportion shares among U, C, and G for the polyadenylation [poly(A)] tail attachment nucleotides for the transcripts that have a pre–messenger RNA non-adenosine nucleotide replaced by the poly(A) tail. (DOCX) [file pone.0079511.s002.docx]

**Table S2.** Proportion shares among U, C, and G for the polyadenylation [poly(A)] tail attachment nucleotides for the transcripts that have a pre–messenger RNA non-adenosine nucleotide replaced by the poly(A) tail.

|  | **Share within non‑A-type sites** | | |  |  |  |
| --- | --- | --- | --- | --- | --- | --- |
| **Species** | **U (%)** | **C (%)** | **G (%)** | **U/C (ratio)** | **U/G (ratio)** | **C/G (ratio)** |
| **Fungi and protozoa** |  |  |  |  |  |  |
| *Neurospora crassa* | 20.0 | 0.0 | 80.0 | NA^a^ | 0.2 | 0 |
| *Plasmodium falciparum* | 0.0 | 0.0 | 0.0 | NA | NA | NA |
| *Schizosaccharomyces pombe* | 0.0 | 0.0 | 0.0 | NA | NA | NA |
| *Trypanosoma cruzi* | 54.2 | 45.8 | 0.0 | 1.2 | NA | NA |
| **Non-mammalian animals** |  |  |  |  |  |  |
| *Apis mellifera* | 76.6 | 5.8 | 17.5 | 13.1 | 4.4 | 0.3 |
| *Caenorhabditis elegans* | 61.6 | 22.1 | 16.3 | 2.8 | 3.8 | 1.4 |
| *Danio rerio* | 36.5 | 31.3 | 32.1 | 1.2 | 1.1 | 1.0 |
| *Drosophila melanogaster* | 48.2 | 33.2 | 18.6 | 1.5 | 2.6 | 1.8 |
| *Gallus gallus* | 24.0 | 41.5 | 34.5 | 0.6 | 0.7 | 1.2 |
| *Taeniopygia guttata* | 23.6 | 40.8 | 35.6 | 0.6 | 0.7 | 1.1 |
| Mean | 45.1 | 29.1 | 25.8 | 1.5 | 1.7 | 1.1 |
| **Mammals—non-primates** |  |  |  |  |  |  |
| *Bos taurus* | 31.2 | 35.3 | 33.5 | 0.9 | 0.9 | 1.1 |
| *Canis lupus familiaris* | 44.4 | 16.7 | 38.9 | 2.7 | 1.1 | 0.4 |
| *Equus caballus* | 18.2 | 45.5 | 36.4 | 0.4 | 0.5 | 1.3 |
| *Mus musculus* | 36.9 | 31.9 | 31.2 | 1.2 | 1.2 | 1.0 |
| *Oryctolagus cuniculus* | 53.5 | 20.9 | 25.6 | 2.6 | 2.1 | 0.8 |
| *Rattus norvegicus* | 44.1 | 27.5 | 28.4 | 1.6 | 1.5 | 1.0 |
| *Sus scrofa* | 30.9 | 39.3 | 29.8 | 0.8 | 1.0 | 1.3 |
| Mean | 37.0 | 31.0 | 32.0 | 1.2 | 1.2 | 1.0 |
| **Mammals—primates** |  |  |  |  |  |  |
| *Callithrix jacchus* | 74.9 | 12.5 | 12.5 | 6.0 | 6.0 | 1.0 |
| *Homo sapiens* | 31.7 | 38.3 | 30.0 | 0.8 | 1.1 | 1.3 |
| *Macaca mulatta* | 24.1 | 48.1 | 27.8 | 0.5 | 0.9 | 1.7 |
| *Pan troglodytes* | 41.1 | 18.2 | 40.7 | 2.3 | 1.0 | 0.4 |
| *Pongo abelii* | 30.6 | 37.3 | 32.1 | 0.8 | 1.0 | 1.2 |
| Mean | 40.5 | 30.9 | 28.6 | 1.3 | 1.4 | 1.1 |
| **Plants** |  |  |  |  |  |  |
| *Arabidopsis thaliana* | 51.2 | 36.4 | 12.4 | 1.4 | 4.1 | 2.9 |
| *Medicago truncatula* | 57.6 | 28.8 | 13.6 | 2.0 | 4.3 | 2.1 |
| *Oryza sativa (japonica)* | 44.1 | 45.2 | 10.8 | 1.0 | 4.1 | 4.2 |
| *Populus trichocarpa* | 43.0 | 45.7 | 11.2 | 0.9 | 3.8 | 4.1 |
| *Solanum tuberosum* | 66.7 | 27.8 | 5.6 | 2.4 | 12.0 | 5.0 |
| *Sorghum bicolor* | 43.4 | 53.4 | 3.2 | 0.8 | 13.5 | 16.7 |
| *Zea mays* | 30.0 | 57.0 | 13.0 | 0.5 | 2.3 | 4.4 |
| Mean | 48.0 | 42.0 | 10.0 | 1.1 | 4.8 | 4.2 |
| **Overall mean** | **39.6** | **34.2** | **26.1** | **1.2** | **1.5** | **1.3** |

^a^NA: not applicable.
