# Supplementary material for: RNA Polyadenylation Sites on the Genomes of Microorganisms, Animals, and Plants
Source: PLoS One. 2013 Nov 18;8(11):e79511. doi: 10.1371/journal.pone.0079511 (PMC3832601; doi:10.1371/journal.pone.0079511)
Supplement: Table S3 — Pre–messenger RNA (mRNA) adenosine replaced by the polyadenylation [poly(A)] tail–normalized frequency with internal priming estimation. (DOCX) [file pone.0079511.s003.docx]

**Table S3.** Pre–messenger RNA (mRNA) adenosine replaced by the polyadenylation [poly(A)] tail—normalized frequency with internal priming estimation^a^.

| **Species** | **Mapped sites (n)** | **12‑A mRNA (n)^b^** | **12‑A (%)** | **Normalized 12‑A (%)^c^** | **Observed A sites (%)** | **Normalized A‑type poly(A) sites (%)^d^** |
| --- | --- | --- | --- | --- | --- | --- |
| **Fungi and parasite protists** |  |  |  |  |  |  |
| *Neurospora crassa* | 38 | 2 | 5.3 | 0.2 | **74** | ***73*** |
| *Plasmodium falciparum* | 42 | 18 | 42.9 | 1.9 | **100** | ***98*** |
| *Schizosaccharomyces pombe* | 26 | 0 | 0.0 | 0.0 | **100** | ***100*** |
| *Trypanosoma cruzi* | 1,523 | 12 | 0.8 | 0.0 | **99** | ***99*** |
| Mean | 407 | 8 | 2.0 | 0.1 | **93** | ***93*** |
| **Non-mammalian animals** |  |  |  |  |  |  |
| *Apis mellifera* | 187 | 48 | 25.7 | 1.1 | **91** | ***90*** |
| *Caenorhabditis elegans* | 392 | 4 | 1.0 | 0.0 | **78** | ***78*** |
| *Danio rerio* | 10,662 | 293 | 2.7 | 0.1 | **89** | ***89*** |
| *Drosophila melanogaster* | 966 | 83 | 8.6 | 0.4 | **97** | ***97*** |
| *Gallus gallus* | 803 | 79 | 9.8 | 0.4 | **82** | ***82*** |
| *Taeniopygia guttata* | 836 | 86 | 10.3 | 0.4 | **79** | ***79*** |
| Mean | 2,308 | 99 | 4.3 | 0.2 | **86** | ***86*** |
| **Mammals** |  |  |  |  |  |  |
| *Bos taurus* | 2,719 | 134 | 4.9 | 0.2 | **83** | ***83*** |
| *Callithrix jacchus* | 118 | 81 | 68.6 | 3.0 | **93** | ***90*** |
| *Canis lupus familiaris* | 125 | 51 | 40.8 | 1.8 | **86** | ***84*** |
| *Equus caballus* | 101 | 33 | 32.7 | 1.4 | **89** | ***88*** |
| *Homo sapiens* | 39,591 | 3,121 | 7.9 | 0.3 | **84** | ***84*** |
| *Macaca mulatta* | 1,152 | 934 | 81.1 | 3.5 | **98** | ***94*** |
| *Mus musculus* | 12,474 | 707 | 5.7 | 0.2 | **87** | ***86*** |
| *Oryctolagus cuniculus* | 316 | 148 | 46.8 | 2.0 | **86** | ***84*** |
| *Pan troglodytes* | 849 | 374 | 44.1 | 1.9 | **97** | ***96*** |
| *Pongo abelii* | 2,036 | 272 | 13.4 | 0.6 | **83** | ***82*** |
| *Rattus norvegicus* | 34,791 | 2,582 | 7.4 | 0.3 | **88** | ***87*** |
| *Sus scrofa* | 12,634 | 3,895 | 30.8 | 1.3 | **89** | ***88*** |
| Mean | 8,909 | 1,028 | 11.5 | 0.5 | **89** | ***87*** |
| **Plants** |  |  |  |  |  |  |
| *Arabidopsis thaliana* | 4,505 | 39 | 0.9 | 0.0 | **76** | ***76*** |
| *Medicago truncatula* | 833 | 3 | 0.4 | 0.0 | **93** | ***93*** |
| *Oryza sativa (japonica)* | 715 | 6 | 0.8 | 0.0 | **87** | ***87*** |
| *Populus trichocarpa* | 1,393 | 15 | 1.1 | 0.0 | **73** | ***73*** |
| *Solanum tuberosum* | 139 | 0 | 0.0 | 0.0 | **87** | ***87*** |
| *Sorghum bicolor* | 1,719 | 1 | 0.1 | 0.0 | **86** | ***86*** |
| *Zea mays* | 21,265 | 15 | 0.1 | 0.0 | **59** | ***59*** |
| Mean | 4,367 | 11 | 0.3 | 0.0 | **80** | ***80*** |
| **Overall mean** | **5,274** | **450** | **17** | **1** | **87** | ***86*** |

^a^Internal priming means the artificial 3′ end with a false poly(A) tail created by oligo (dT) annealing to the multiple-adenosine sequencing inside an mRNA sequence during the conversion from mRNA to complementary DNA prior to DNA sequencing.

^b^The mapped genomic DNA region has a 12‑A sequence immediately after the mapped poly(A) site; therefore, a question can be raised about whether the poly-adenosine sequence of that mRNA sequence in the database represents internal priming or indeed the true poly(A) tail.

^c^*p* = *qx*/3, where *p* is the artificially increased adenosine site frequency due to internal priming, *q* is the percentage of the mRNA that has 12 A’s immediately starting from the mapped poly(A) site on the genomic DNA, and *x* is the percentage of the non‑A-type poly(A) site type (it is 1 − 0.87 = 0.13 according to the overall mean in the table). The “/3” means that the estimated chance for priming at the internal multiple A’s is approximately three times smaller than the chance for priming from the true poly(A) tail, because the poly(A) tail is usually 3 to 10 times longer than the internal multiple A’s. The product of *q* times *x* is used because internal priming can modify the calculated poly(A) site adenosine frequency only when the true poly(A) site is a non-adenosine nucleotide. Internal priming does not change the calculated percentage if the true poly(A) site of that mRNA is an adenosine already.

^d^Normalized A‑type poly(A) site (%) = observed A% − normalized 12‑A%.
