# Supplementary material for: RNA Polyadenylation Sites on the Genomes of Microorganisms, Animals, and Plants
Source: PLoS One. 2013 Nov 18;8(11):e79511. doi: 10.1371/journal.pone.0079511 (PMC3832601; doi:10.1371/journal.pone.0079511)
Supplement: File S1 — Estimation of the theoretical A-type polyadenylation [poly(A)] site frequency in the random model. (DOCX) [file pone.0079511.s004.docx]

**File S1.** Estimation of the theoretical A‑type polyadenylation [poly(A)] site frequency in the random model.

For an A‑type poly(A) site, it is unknown whether this A nucleotide in the precursor mRNA corresponding to the first A in the poly(A) tail is replaced by the poly(A) tail or is simply extended by adding the poly(A) tail. However, the theoretical frequency of the A‑type poly(A) sites is the same regardless of whether this nucleotide is replaced or simply extended if the four nucleotides (A, C, G, and U) are randomly chosen for the poly(A) site. The calculation is explained below.

In the replacement model, if the poly(A) site was totally random in choosing A, C, G, or U, the theoretical percentage of A‑type poly(A) sites would be equal to the average A nucleotide content of the 3′ region. However, if a non‑A-type nucleotide (C, G, or U) was replaced by the poly(A) and if the directly adjacent upstream position is an A nucleotide, this adjacent upstream A will be aligned as the first A of the poly(A) tail. Therefore the theoretical A‑type poly(A) site from the alignment in the random model would be equal to “the percentage of A in mRNA” plus “the frequency of A at the position adjacent to the non‑A poly(A) sites”. If the A nucleotide percentage in mRNA is *p*, the A‑type poly(A) site from the alignment will be *p* + *p*(1 − *p*) = *p*(2 − *p*), where (1 − *p*) is the non‑A nucleotide content. The multiple‑A or multiple-non‑A sequences do not alter the A site or non‑A site probability in this random model, because both A and non‑A have a random chance in this aspect within their nucleotide content ranges.

Similarly, if the nucleotide in precursor mRNA corresponding to the first A of the poly(A) tail is not replaced but instead used as the starting point for adding the poly(A) tail, the theoretical value of the A‑type poly(A) tail in the alignment is still 51.0% in this extending model, but *p*(1 − p) is counted from the downstream A directly adjacent to the poly(A) site.
